# Modular Synthesis of Substituted Lactams via a Deoxygenative Photochemical Alkylation–Cyclization Cascade of Secondary Amides in Flow

**DOI:** 10.1021/jacsau.5c00884

**Published:** 2025-09-08

**Authors:** Damiano Diprima, Thomas Terp Paulsen, Antonio Pulcinella, Stefano Bonciolini, Alexis L. Gabbey, Robin Stuhr, Thomas Bjørnskov Poulsen, Timothy Noël

**Affiliations:** † Flow Chemistry Group, Van’t Hoff Institute for Molecular Sciences(HIMS), 7938 University of Amsterdam, Science Park 904, Amsterdam 1098 XH, The Netherlands; ‡ Department of Chemistry, Aarhus University, Langelandsgade 140, 8000 Aarhus C, Denmark; § Department of Chemistry, University of Toronto, 80 St. George Street, Toronto, Ontario M5S 3H6, Canada; ∥ Department of Chemistry, University of Hamburg, Hamburg 20146, Germany

**Keywords:** photochemistry, lactam synthesis, flow cascade, secondary amide activation, photochemical alkylation, flow chemistry

## Abstract

γ-Lactams are crucial scaffolds in many bioactive compounds and pharmaceutical agents, yet their synthesis featuring diverse γ- and *N*-substitution remains a significant synthetic challenge. Current methods often lack modularity and efficiency, particularly when targeting sterically hindered or highly functionalized analogues. Herein, we report a modular, three-step strategy for the systematic synthesis of γ- and *N*-substituted γ-lactams from readily available primary amines and carboxylic acids. The sequence includes deoxygenative activation of secondary amides using triflic anhydride, a photochemical silane-mediated radical alkylation, and intramolecular cyclization. The alkylation–lactamization cascade proceeds under additive-free, continuous-flow photochemical conditions, enabling rapid reaction times (20 min) and scalable operation. Compared to conventional *N*-alkylation approaches, this method broadens access to sterically hindered analogues and offers a valuable platform for medicinal chemistry applications.

## Graphical abstract

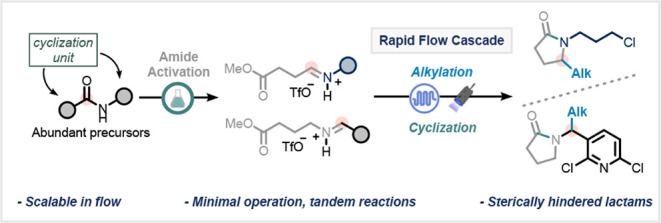

γ-Lactams, a class of five-membered nitrogen-containing heterocycles, are common motifs in many pharmaceuticals and bioactive molecules (Figure 1a).^1–5^ Most marketed γ-lactam-based drugs and natural products exhibit structural diversity enabled by substitution at three distinct positions on the lactam ring (α, β, γ), as well as at the nitrogen atom.^6^ These variations critically influence biological activity and molecular stability. Beyond their therapeutic relevance, γ-lactams are valuable synthetic intermediates, owing to the broad reactivity of the amide functional group.^7–10^

**Figure 1:**
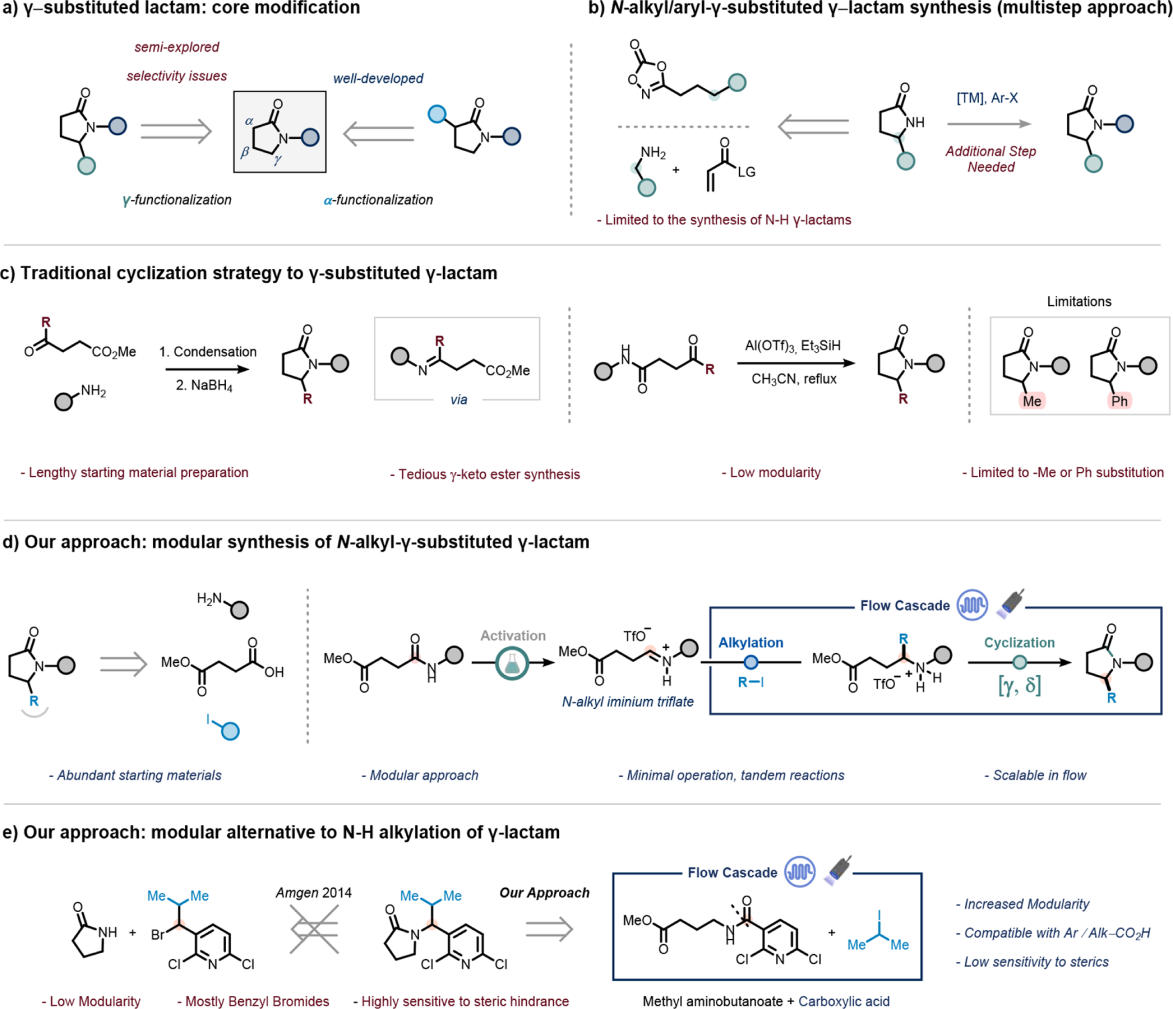
Conventional strategies to access substituted γ-lactam motifs and our alkylation–cyclization flow cascade.

Strategies for synthesizing decorated *N*-substituted γ-lactams generally fall into two categories: direct modification of functionalized five-membered lactam cores or intramolecular cyclization of bifunctional precursors.^6,11–17^ Regarding the former, α-functionalization is well established, leveraging the acidity of the α-proton to enable efficient deprotonation and subsequent C–C bond formation via alkylation or palladium-catalyzed cross-coupling (Figure 1a).^18–20^ These methods offer reliable access to alkyl- and aryl-substituted derivatives. In contrast, γ-functionalization remains more challenging. It typically relies on C–H functionalization via hydrogen atom transfer (HAT),^21–24^ which often suffers from poor regioselectivity, particularly in the presence of *N*-substitution. Under these conditions, exofunctionalization competes with γ-selectivity, complicating the targeted modification (Figure 1a).^25^

Recent advances have enabled the synthesis of γ-substituted γ-lactams from simple and abundant starting materials such as carboxylic acids and amines. Notably, C–H amidation using dioxazolones as nitrenoid precursors and photocatalytic α-functionalization of primary amines have expanded chemical space under mild conditions.^26–32^ However, these approaches do not directly yield *N*-substituted analogues, requiring an additional *N*-alkylation or cross-coupling step to access the desired products (Figure 1b).^33–40^

One of the most intuitive and widely used strategies for constructing γ-lactam rings involves reductive amination between an amine and a γ-keto ester, followed by intramolecular cyclization (Figure 1c, left).^41–43^ A similar reductive cyclization sequence can be also performed using γ-amino acids and ketones.^44–48^ Alternatively, other approaches using γ-keto-amides are described in literature (Figure 1c, right).^49^ While effective, these methods typically require the prior synthesis of highly functionalized intermediates, which limits modularity, an essential feature for medicinal chemistry and library design. This limitation is reflected in the narrow scope of reported substituents at the γ-position, often restricted to methyl or phenyl groups (Figure 1c).^41–43,49^ Despite notable advances, general and modular approaches that enable simultaneous and precise control over both γ- and *N*-substitution remain underdeveloped. Such methods would be highly valuable for efficiently accessing structurally diverse γ-lactams in drug discovery applications.

To address these limitations, we present a streamlined, modular strategy for synthesizing *N*- and γ-substituted γ-lactams from abundant, readily available starting materials. Building on our previous work in α-branched secondary amine synthesis,^50^ this three-step approach begins with the formation of secondary amides from monomethyl succinate and primary amines. These intermediates undergo deoxygenative activation with triflic anhydride to generate *N*-alkyl iminium triflates,^51–53^ which are then subjected to photochemical, silane-mediated radical alkylation with alkyl iodides to afford α-alkylated γ-amino esters.^54,55^

A final intramolecular cyclization yields the desired *N*-alkyl, γ-substituted γ-lactams. Notably, the photochemical alkylative lactamization cascade smoothly occurs within the same photochemical reactor under continuous flow conditions, without the addition of any external additive (Figure 1d).

In addition to addressing the modularity challenges outlined above, our method offers a robust alternative to traditional *N*-alkylation of 2-pyrrolidones, a common yet synthetically constrained route to γ-lactams.^40,56–58^ In this context, researchers at Amgen reported significant limitations with this approach, including high sensitivity to steric hindrance and narrow electrophile scope—restricted primarily to benzyl bromides—thereby requiring multiple steps to access more diverse analogues.^59^ In contrast, our strategy circumvents these constraints by employing abundant aliphatic carboxylic acids and methyl 4-aminobutanoate as the key cyclization unit, enabling direct access to sterically hindered *N*-branched γ-lactams in a single streamlined sequence (Figure 1e).

Moreover, with increasing emphasis on high-throughput experimentation and library synthesis, continuous-flow platforms have emerged as ideal tools for enabling modularity and reaction control.^60–63^

Photochemical transformations, in particular, benefit from the enhanced irradiation efficiency of flow systems.^64^ By implementing our alkylation–lactamization cascade under continuous flow, we reduce reaction times from 48 h in batch to just 20 min. Additionally, the precise control offered by flow allows selective interruption of the cascade, isolating γ-amino ester intermediates in as little as 2 min. Collectively, this method provides a practical, scalable solution for rapidly accessing a wide array of highly substituted γ-lactams from inexpensive, readily available feedstocks.

We started our investigation with the deoxygenative semireduction of secondary amide **1a** to generate the corresponding iminium triflate intermediate **Int-A**. Treatment of **1a** with 2-fluoropyridine (1.2 equiv), triflic anhydride (1.1 equiv), and triethylsilane in acetonitrile (0.25 M) afforded **Int-A** in 96% yield (see Section S7.1).^51^

Building on our previous work involving iminium ion alkylation,^50^ a one-pot protocol was developed in which isopropyl iodide (3.0 equiv), tris­(trimethylsilyl)­silane (TTMS, 2.0 equiv), and acetonitrile (to reach a final concentration of 0.05 M) were directly added to the crude iminium triflate solution. The reaction mixture was then irradiated with 390 nm LED-light for the indicated time (see Section S7.2 for preliminary results and model substrate selection).

Careful optimization of the reaction parameters revealed that extending the irradiation time to 48 h afforded 80% overall yield of a 7:1 mixture of the target γ-lactam (**3**) and the linear alkylated γ-amino ester (**2**). We then examined the impact of reagent stoichiometry on the efficiency of the alkylation–lactamization cascade.

Reducing the equivalents of TTMS and alkyl iodide—previously shown to have minimal influence on alkylation alone—led to diminished lactam formation (Table 1, entries 2 and 3). Specifically, decreasing TTMS from 2.0 to 1.1 equiv reduced the yield of **3** without affecting the overall alkylation efficiency (combined yield of **2** and **3**, entry 2). In contrast, lowering the alkyl iodide loading from 3.0 to 1.5 equiv impaired both alkylation and subsequent cyclization (entry 3).

**Table 1:**
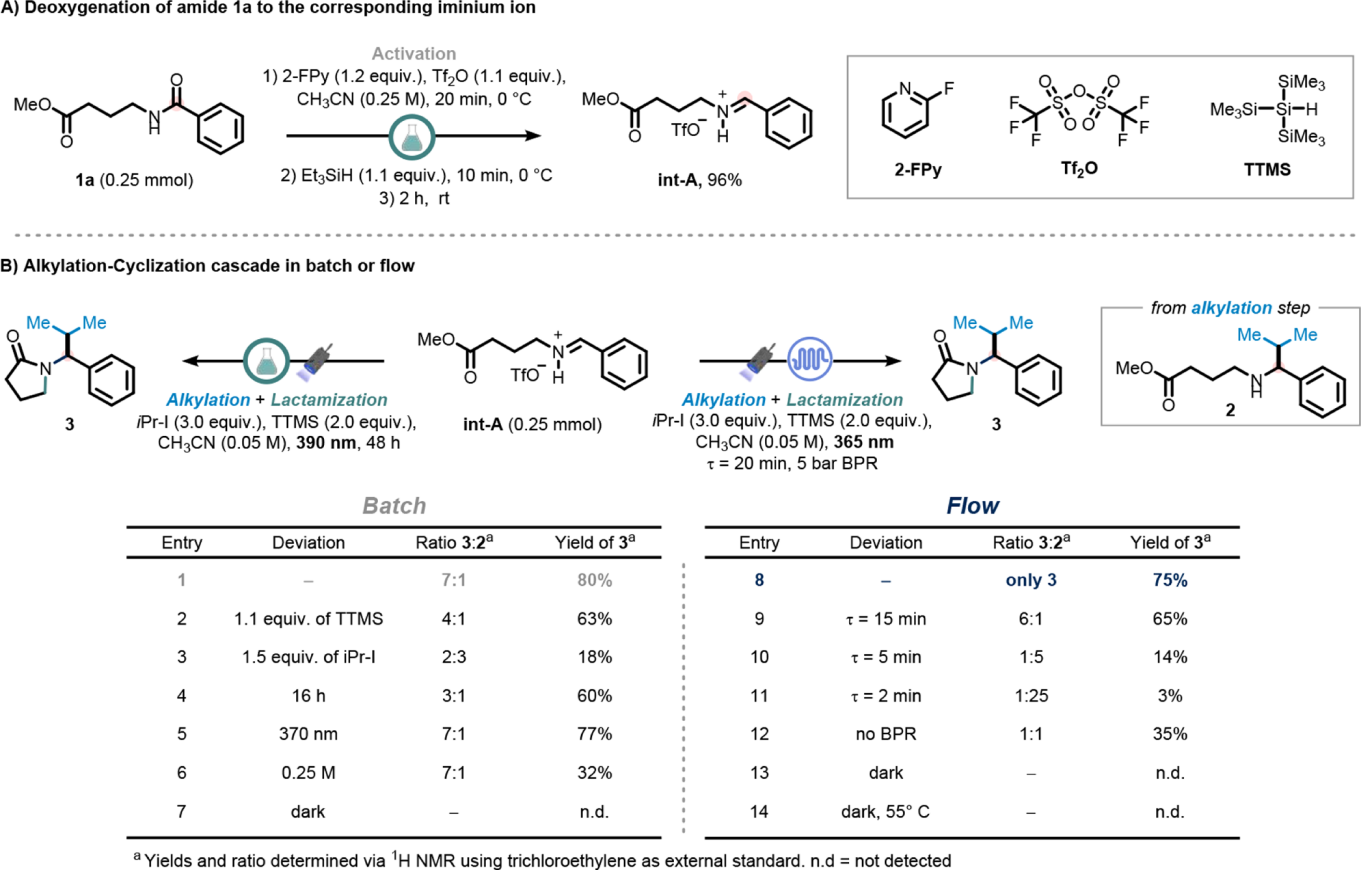
Optimization of the Alkylation–Cyclization Cascade

Shortening the irradiation time to 16 h had little effect on alkylation but reduced the formation of **3**, consistent with a stepwise mechanism (entry 4). Using a higher energy light source (370 nm) resulted in no significant change in outcome (entry 5). Finally, increasing the reaction concentration led to a lower yield of the cyclized product (**3**), likely due to reduced light penetration (entry 6; see Section S7.3).

Due to the impractically long irradiation times and incomplete cyclization observed in batch, we transitioned the reaction to continuous flow using a Signify Eagle Reactor (see Supporting Information, Sections 2.2 and 7.5).^76^ Leveraging a high-intensity chip-on-board LED system (λ = 365 nm, 144 W optical power) and the inherent advantages of flow photochemistry,^77^ the overall reaction time was reduced dramatically—from 48 hours to just 20 minutes (entry 8). Attempts to shorten the residence time further resulted in progressively lower yields of γ-lactam **3** (entries 9–11), underscoring the importance of sufficient irradiation time.

Cyclization efficiency also declined when using a lower-intensity commercial flow reactor, highlighting the critical role of light power in driving the cascade (see Section S7.6).^65^ To ensure consistent flow rates and residence times, a 5 bar back-pressure regulator (BPR) was used to mitigate the effects of gas evolution,^66^ likely due to propane formation from the reduction of isopropyl iodide (entry 12). Finally, control experiments confirmed the photochemical nature of the process: no formation of either the linear intermediate **2** or the lactam **3** was observed in the absence of light or under thermal conditions alone (55 °C, entries 13–14).

With the optimized reaction conditions in hand, we next evaluated the generality of the alkylation–lactamization cascade by systematically varying all three coupling partners—the amine, carboxylic acid, and alkyl iodide—to demonstrate the modularity and versatility of the protocol in synthesizing diverse γ-lactams.

First, we assessed the effect of the amine fragment by subjecting the corresponding amide derived from the coupling with monomethyl succinate, to the standard reaction conditions (Figure 2). Notably, linear primary amines bearing distal phenyl, ester and chloride functional groups were well-tolerated and afforded the desired lactam in good yields with no detectable amount of linear amino ester (**4**, **5**, **6**). In a similar fashion, primary amines with cyclic aliphatic fragments also performed well, yielding the desired lactams in moderate to good yields (**7**, **8**). We then evaluated different alkyl iodides in the alkylation-lactamization cascade, using methyl 4-oxo-4-((3-phenylpropyl)­amino)­butanoate **1b** as model substrate (Figure 2). Primary (**9**, **10**), secondary (**11**, **12**) and tertiary (**13**) alkyl iodides yielded the desired γ-substituted γ-lactam in moderate to good yields. Notably, the incorporation of a terminal alkyne group (**9**) was achieved, providing a handle for downstream diversification via click chemistry^67,68^ or Sonogashira coupling.^69^

**Figure 2:**
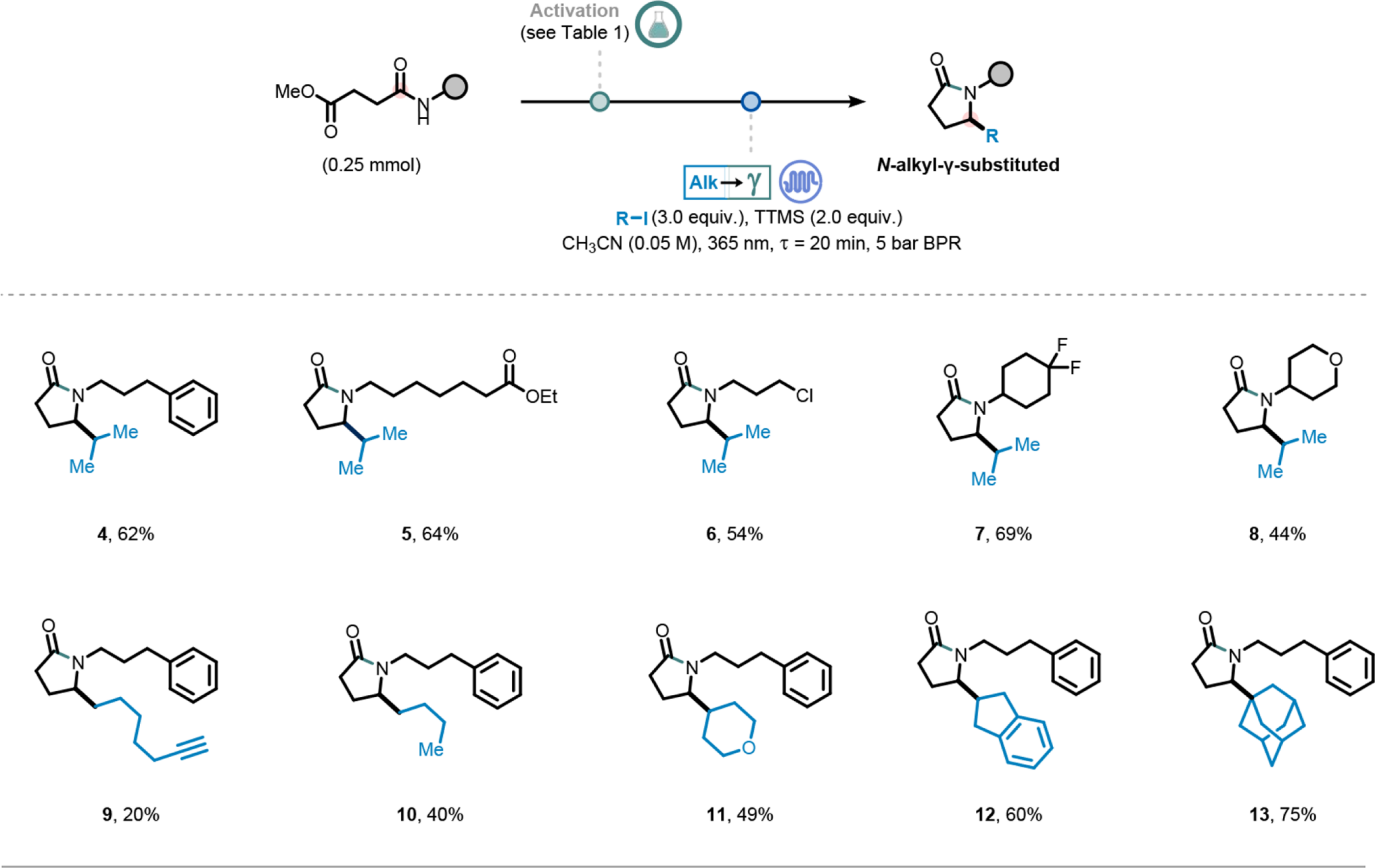
Synthesis of *N*-alkyl-γ-substituted γ-lactams in flow.

We next evaluated the scope of the deoxygenative alkylative lactamization using amides derived from methyl 4-aminobutanoate, focusing on the variation of the carboxylic acid component (Figure 3). This investigation further demonstrates the ability of our cascade process to overcome the steric and modularity limitations associated with traditional *N*-alkylation of 2-pyrrolidones. Aromatic carboxylic acids, including benzoic acid and derivatives bearing bromine, oxadiazole, and secondary sulfonamide substituents, were well tolerated, affording the corresponding γ-lactams in good yields (**3**, **14**–**16**).

**Figure 3:**
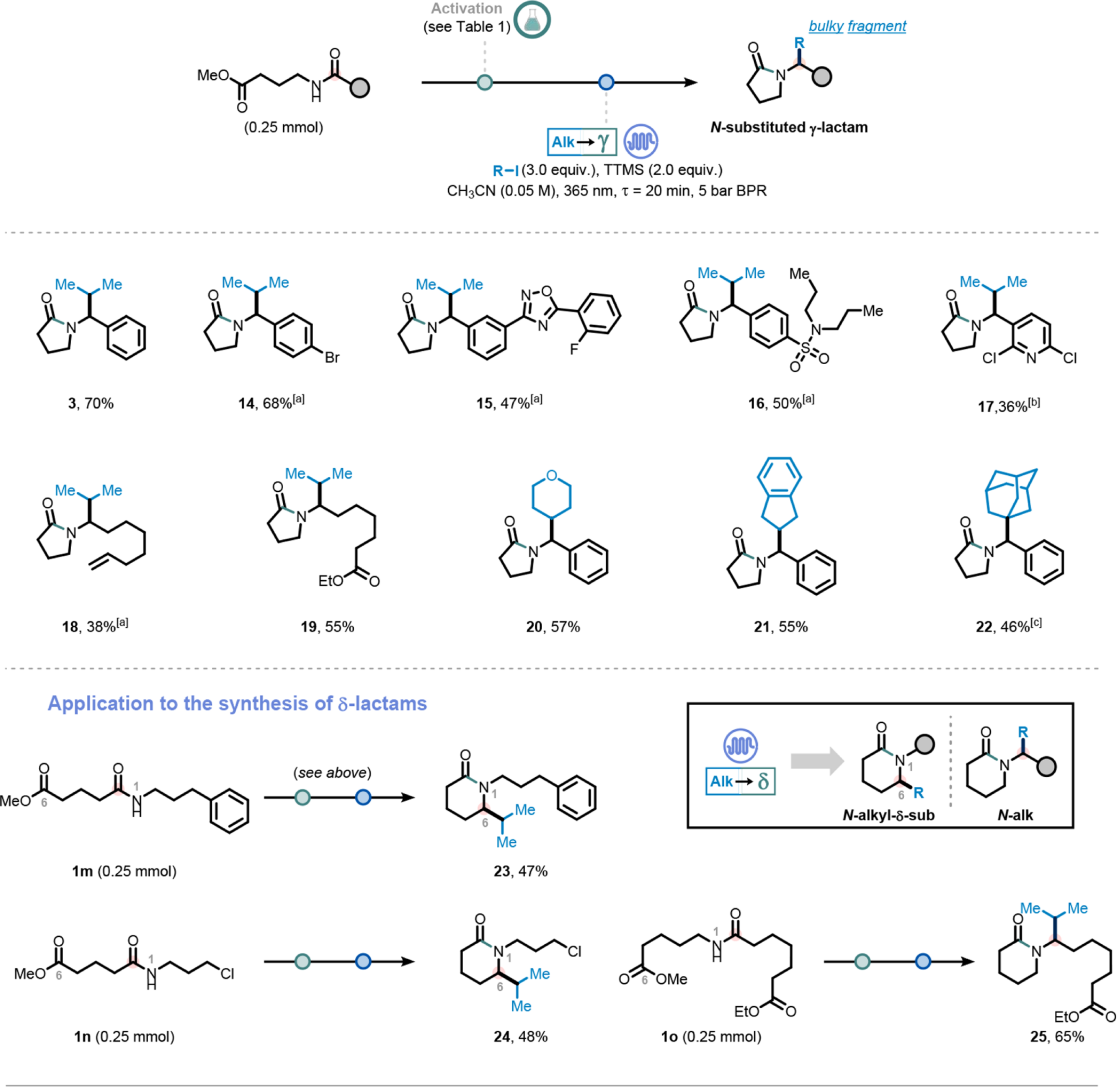
Synthesis of *N*-substituted γ-lactams in flow and explorative access to δ-lactams (R = bulky fragment). ^[a]^The reaction was performed in batch (see Supporting Information). ^[b]^Activation was performed at −78 °C (see Supporting Information). ^[c]^Residence time (i) = 30 min.

Notably, a bis-chlorinated pyridine derivative (**17**) was also compatible, albeit requiring activation at −78 °C. Additionally, two linear aliphatic carboxylic acids bearing respectively a terminal alkene (**18**) and an ethyl ester (**19**) were successfully converted, providing synthetic handles for product diversification. We further evaluated the tolerance to sterically demanding *N*-substituents by employing primary, secondary, and tertiary alkyl iodides. Under optimized conditions, the cascade successfully delivered the corresponding hindered *N*-alkylated γ-lactams in moderate to good yields (**20**–**22**), underscoring the low sensitivity of the protocol to steric hindrance. For comparison, the N–H alkylation protocol reported by Amgen fails with isopropyl electrophiles and is incompatible with secondary and tertiary alkyl residues.

Encouraged by these results, we investigated the applicability of the cascade to alternative lactam ring sizes; it was possible to synthesize both sterically hindered *N*-substituted δ-lactam and *N*-alkyl-δ-substituted δ-lactams in moderate to good yield (**23**–**25**). However, attempts to generate four-membered β-lactams or seven-membered ε-lactams under analogous conditions were unsuccessful, yielding only the corresponding linear amino ester intermediates (see Section S10). Importantly, all γ- and δ-lactams reported herein are, to our knowledge, previously unreported in the literature, highlighting the synthetic utility and novelty of this modular flow-based platform.

To gain insight into the mechanism of the alkylative lactamization cascade, a series of mechanistic experiments were performed. Based on our previous work and relevant literature, we propose that the alkylation step is initiated by light-induced homolysis of the alkyl iodide, generating an alkyl radical that adds to the iminium ion. This results in the formation of an electrophilic aminium radical cation, which undergoes hydrogen atom transfer (HAT) with tris­(trimethylsilyl)­silane (TTMS) to produce the alkylated ammonium triflate. The resulting TTMS radical then engages in halogen atom transfer (XAT) with a second equivalent of alkyl iodide, propagating the radical chain and forming tris­(trimethylsilyl)­silyl iodide (TTMS-I) as a byproduct.^50,54,55,70^

Kinetic studies conducted under continuous-flow conditions revealed that the iminium triflate intermediate (**Int-A**) is rapidly consumed within 2 min, producing the linear γ-amino ester (**2**) in 75% yield, as determined by ^1^H NMR (<5% of **3**). Over the course of 20 min, **2** undergoes complete cyclization to yield the γ-lactam **3**, indicating that the lactamization step occurs sequentially under the same reaction conditions (Figure 4a and see Section S9.1).

**Figure 4:**
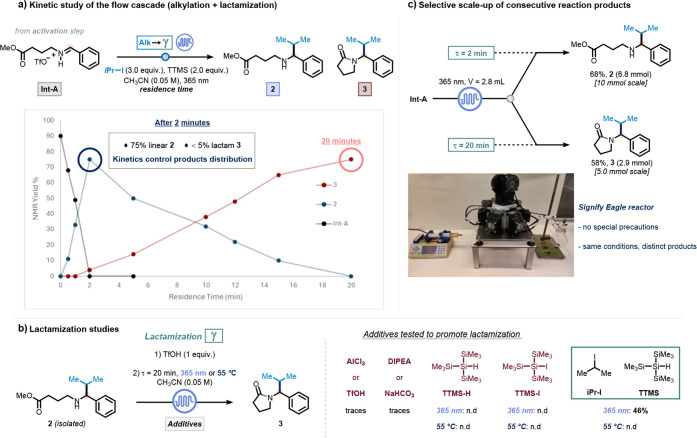
Reaction kinetic of the flow cascade (a), additives screening (b), and selective scale-up (c).

To further elucidate the nature of the lactamization step, we carried out an extensive additive screening, starting from isolated intermediate **2**. Additives derived from the activation step, including 2-fluoropyridine, triethylsilane, and their byproducts, were found to be inactive in promoting cyclization (see Section S9.3.1). Additionally, both Brønsted and Lewis acid-mediated thermal pathways, as well as base-promoted cyclization, were ruled out (see Section S9.2 and Table S8). Neither TTMS nor TTMS-I alone enabled conversion of **2** to **3**, under either thermal or photochemical conditions; in all cases, **2** was recovered quantitatively (Figure 4b and see Section S9.3.2). Strikingly, lactam formation was observed only when TTMS and iPr-I were present under irradiation, suggesting a synergistic role of both TTMS and TTMS-I in promoting cyclization (see Section S9.3.2).

Finally, we demonstrated the scalability and divergent utility of the flow protocol for the selective synthesis of either the linear alkylated γ-amino ester (**2**) or the corresponding *N*-substituted γ-lactam (**3**).^71,72^ The initial triflic anhydride activation was carried out on a 5 or 10 mmol scale in batch, followed by the photochemical step in flow. A short residence time of 2 min enabled the selective isolation of intermediate **2** in 68% yield. Extending the residence time to 20 min under otherwise identical conditions afforded the cyclized product **3** in 58% yield (Figure 4c and see Section S8). These results underscore the modularity, tunability, and preparative potential of the developed flow-based cascade.

In summary, we have developed a modular, continuous-flow cascade for the synthesis of γ- and *N*-substituted γ-lactams from abundant and inexpensive starting materials. This three-step protocol, comprising deoxygenative amide activation, photochemical radical alkylation, and intramolecular cyclization, provides a robust alternative to conventional *N*-alkylation and cyclization strategies. The method addresses key limitations, such as poor modularity, limited electrophile scope, and steric sensitivity. Integration with flow technology enables shorter reaction times, tunable product selectivity, and scalable operation. Collectively, this approach offers a practical and versatile platform for accessing structurally diverse γ-lactams, facilitating their use in drug discovery campaigns.

## Supplementary Material



## Notes

Detailed experimental procedures, including photographs of the experimental setup, mechanistic study experiments, compound characterization data, and copies of NMR spectra (PDF)
