# Supplementary material for: Modular Synthesis of Substituted Lactams via a Deoxygenative Photochemical Alkylation–Cyclization Cascade of Secondary Amides in Flow
Source: JACS Au. 2025 Sep 8;5(9):4584–92. doi: 10.1021/jacsau.5c00884 (PMC12458026; doi:10.1021/jacsau.5c00884)

Supplementary Information

Modular synthesis of substituted lactams via deoxygenative photochemical alkylation-cyclization cascade of secondary amides in flow

*Damiano Diprima,[1,†] Thomas Terp Paulsen,[1,2,†] Antonio Pulcinella,[1,†] Stefano Bonciolini,[1] Alexis L. Gabbey,[1,3] Robin Stuhr,[1] Thomas Bjørnskov Poulsen[2] and Timothy Noël[1,]**

[1] Flow Chemistry Group, Van 't Hoff Institute for Molecular Sciences (HIMS), University of Amsterdam, Science Park 904, 1098 XH Amsterdam, The Netherlands.

[2] Department of Chemistry, Aarhus University, Langelandsgade 140, 8000 Aarhus C, Denmark.

[3] Department of Chemistry, University of Toronto, 80 St. George Street, Toronto, Ontario M5S 3H6, Canada.

[†] These authors contributed equally to this work.

* *Email: t.noel@uva.nl*

Table of Contents

1. General information ... 4

2. Reactor Design ... 5

2.1. Flow equipment ... 5

2.2. Eagle Reactor ... 6

2.3. Vapourtec Reactor ... 7

2.4. 4 or 8 vials photoreactor (UFO reactor) ... 8

3. Chart of starting materials used in the scope ... 9

4. Synthesis of Starting Materials ... 10

4.1. General procedure 1 (GP1) for the preparation of secondary amides from benzoyl chloride 10

4.2. General procedure 2 (GP2) for the preparation of secondary amides from carboxylic acids 10

4.3. General Procedure 3 (GP3) for the preparation of alkyl iodides ... 10

5. General procedure 4 (GP4): Deoxygenative alkylation of amides and lactamization (activation at 0 ˚C) ... 11

6. General procedure 5 (GP5): Deoxygenative alkylation of amides and lactamization (activation at -78 ˚C) ... 12

7. Reaction optimization ... 13

7.1. Iminium ion **Int-A** formation in CH_3CN ... 13

7.2. Preliminary results in batch: effect of the basic work-up and selection of the model substrate 14

7.3. Reaction optimization in batch ... 15

7.4. Test of the generality of the reaction conditions in batch ... 16

7.5. Translation from batch to flow ... 17

7.6. Vapourtec Optimization (Effect of different set-up and light intensity) ... 18

8. Scale up ... 19

9. Mechanistic investigation ... 21

9.1. Reaction kinetics of the flow cascade (alkylation + cyclization) ... 21

9.2. Lactamization screening under thermal conditions in batch ... 22

9.3. Lactamization studies from linear α-branched amine (**2**) ... 23

9.3.1. Influence of the activation conditions mixture on the lactamization of **2-OTf** ... 23

9.3.2. Additives effect on the lactamization of linear α-branched amine (**2**) ... 24

10. Limitations ... 27

11. Characterization data of synthesized compounds ... 28

11.1. Characterization of compounds **1a-1o** (*secondary amides*) ... 28

11.2. Characterization of compounds **3-25** (*lactams*) ... 33

12. References ... 41

13. NMR spectra of starting materials ... 42

14. NMR spectra of products 60

1. General information

All reagents and solvents were used as received without further purification, unless stated otherwise. Reagents and solvents were bought from Sigma Aldrich, TCI, Fluorochem, BLD Pharm and Fisher Scientific and, if applicable, kept under nitrogen atmosphere. Technical solvents were bought from VWR International and Biosolve, and were used as received. Disposable syringes were purchased from Laboratory Glass Specialist. Product isolation was performed manually, using silica (P60, SILICYCLE) or automatically, using Biotage® Isolation Four, with Biotage® SNAP KP-Sil 4 or 10 g flash chromatography cartridges. TLC analysis was performed using Silica on aluminum foils TLC plates (F254, Supelco Sigma-Aldrich™) with visualization under ultraviolet light (254 nm and 365 nm) or appropriate TLC staining (cerium ammonium molybdate or potassium permanganate). ^{1}H (300 MHz or 400 MHz), ^{13}C (101 MHz), ^{19}F NMR (282 MHz), spectra were recorded unless stated otherwise at ambient temperature using a Bruker AV400 III, Bruker AV400 or a Bruker AV300. ^{1}H NMR spectra are reported in parts per million (ppm) downfield relative to $CDCl_3$ (7.26 ppm) and all ^{13}C NMR spectra are reported in ppm relative to $CDCl_3$ (77.16 ppm) unless stated otherwise. The following abbreviations have been adopted to describe the multiplicity: bs (broad singlet), s (singlet), d (doublet), t (triplet), q (quartet), p (pentet), h (hextet), hept (heptet), m (multiplet), dd (double doublet), td (triple doublet), tt (triplet of triplets). Coupling constants (*J*) are reported in hertz (Hz). NMR data were processed using the MestReNova 14.3.0 software package. Known products were characterized through comparison with the corresponding ^{1}H NMR and ^{13}C NMR from literature. High resolution mass spectra (HRMS) were collected on an AccuTOF LC, JMS-T100LP Mass spectrometer (JEOL, Japen.)

The names of all products were generated using the PerkinElmer ChemBioDraw Ultra v.12.0.2 software package.

For the photochemical batch experiments, a 3D-printed (PLA) reactor internally coated with aluminum foil and equipped with a specific 3D-printed (PLA) lid serving as vials holder and lamp holder was used (see section 2.4 for details).
For the photochemical flow experiments a photoreactor produced by Signify was used (see section 2.2 for details).

2. Reactor Design

2.1. Flow equipment

Syringe pump (Chemyx Fusion 200)

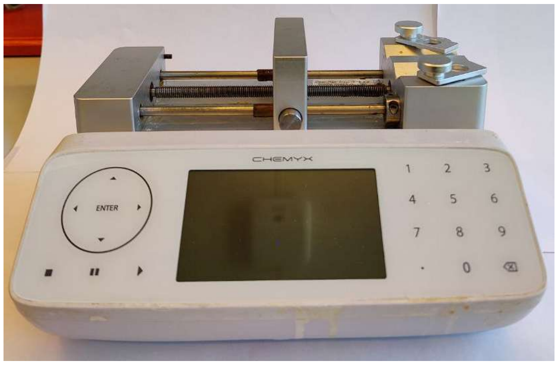

Gastight syringe SGE Luer Lock 10 mL

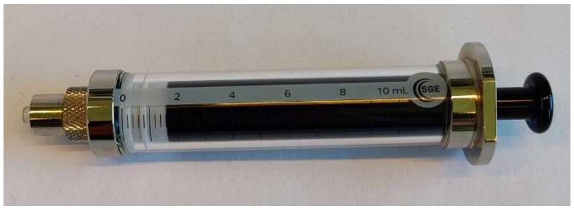

Shut off valve

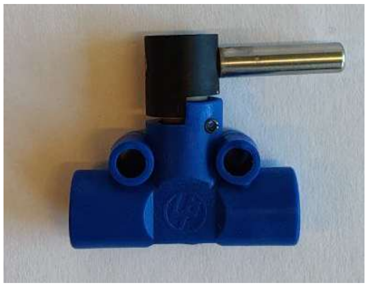

BPR cartridge

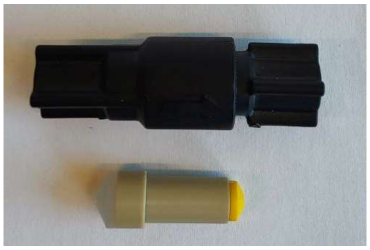

Check valve

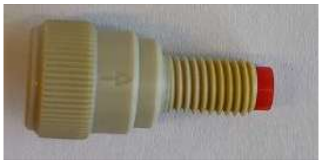

2.2. Eagle Reactor

A Signify photochemical reactor is used, consisting of a base assembly with six 365 nm UV-A chip-on-board light modules. Each module comprises a heath sink air cooled using individual fans. The reaction chamber is further cooled using an additional fan blowing air through a metal pipe used to coil the PFA capillary reactor. The air cooling and the LED intensity can be independently controlled and modified. The six LED modules (365 nm, max. 144 W combined optical output power) are positioned in hexagonal form around an aluminum cylinder support (80 mm height, 75 mm diameter), which has the reactor coil wrapped around (PFA capillary tubing: 1.6 mm OD, 0.8 mm ID, 2.8 mL volume).

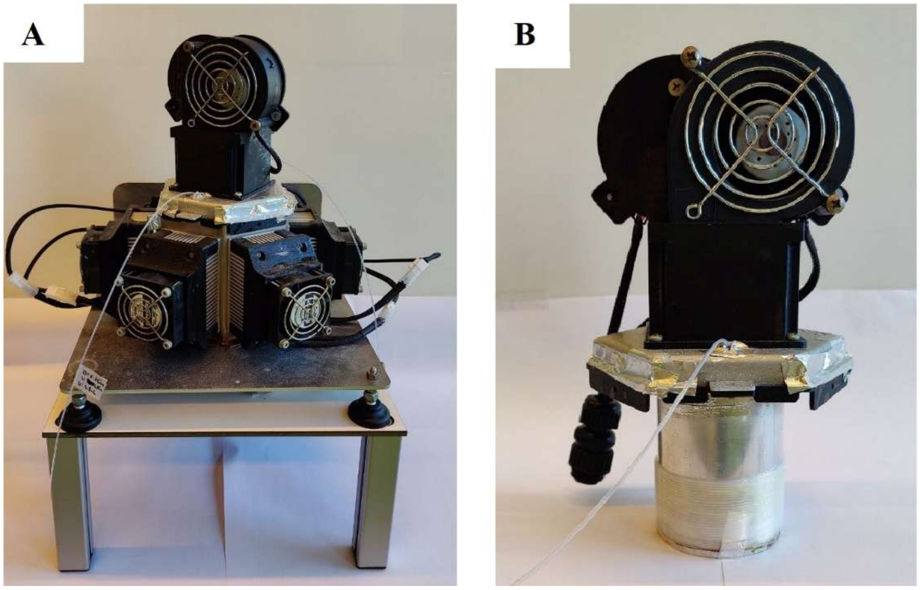

Supplementary Figure 1: Signify's (Eagle) photoreactor 365 nm (144 W)

2.3. Vapourtec Reactor

A Vapourtec UV-150 photochemical reactor is used, equipped with the 365 nm 150 W (input power) High power LED. The reaction chamber was cooled using compressed air to keep the temperature around 25 °C. The reactor coil FEP capillary tubing: (1.6 mm OD, 0.8 mm ID, 3.0 mL volume)

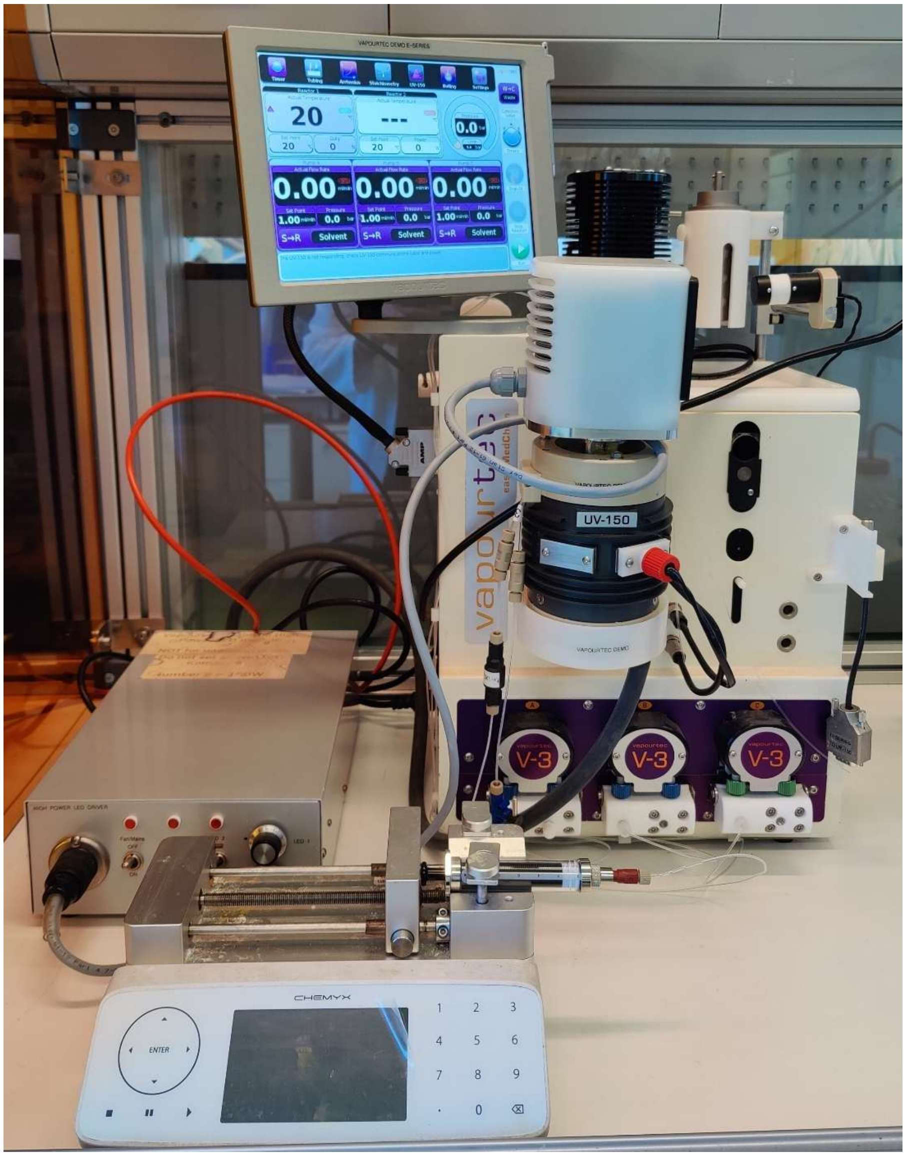

Supplementary Figure 2: Vapourtec set-up for flow experiments.

2.4. 4 or 8 vials photoreactor (UFO reactor)

Reactions were irradiating simultaneously using the photoreactor described below. A 52W Kessil PR160L-390 nm or 370 nm was used as LED lamp, while the temperature was maintained around 35-40 °C via a fan positioned under the reactor. The assembled set-up was placed behind UV-light shielding amber acrylic for all duration of the reaction.[1]

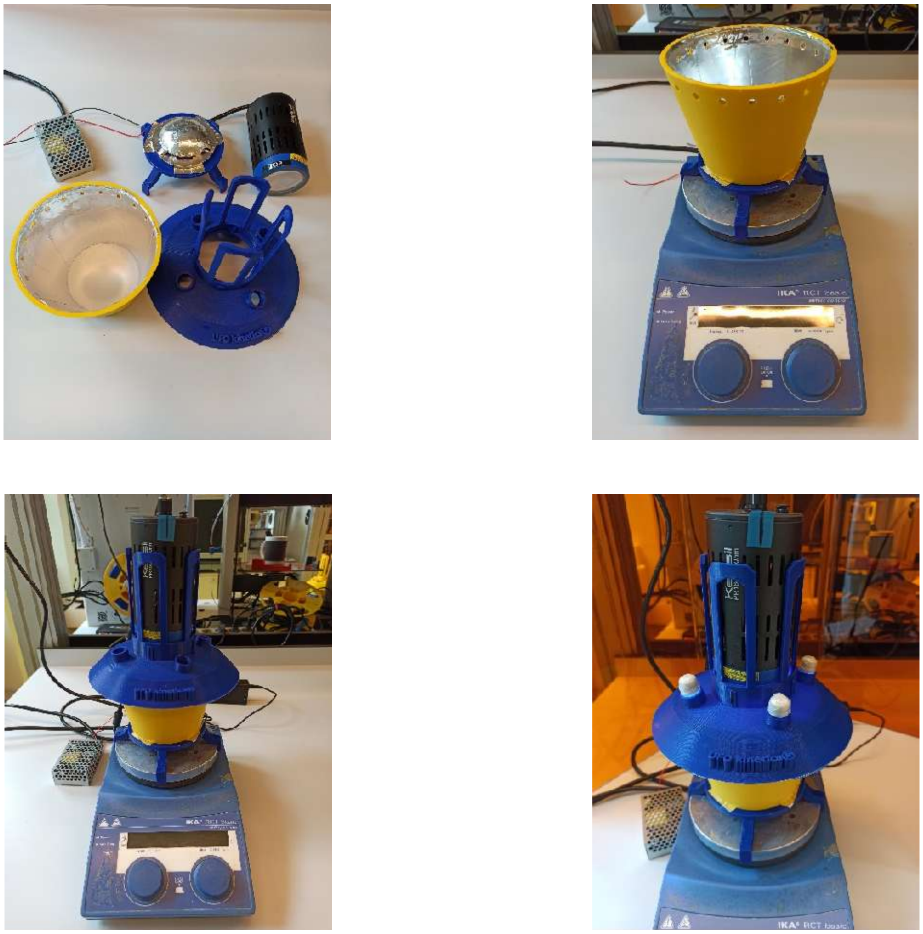

Supplementary Figure 3: Photoreactor used for the optimization and scope.

3. Chart of starting materials used in the scope

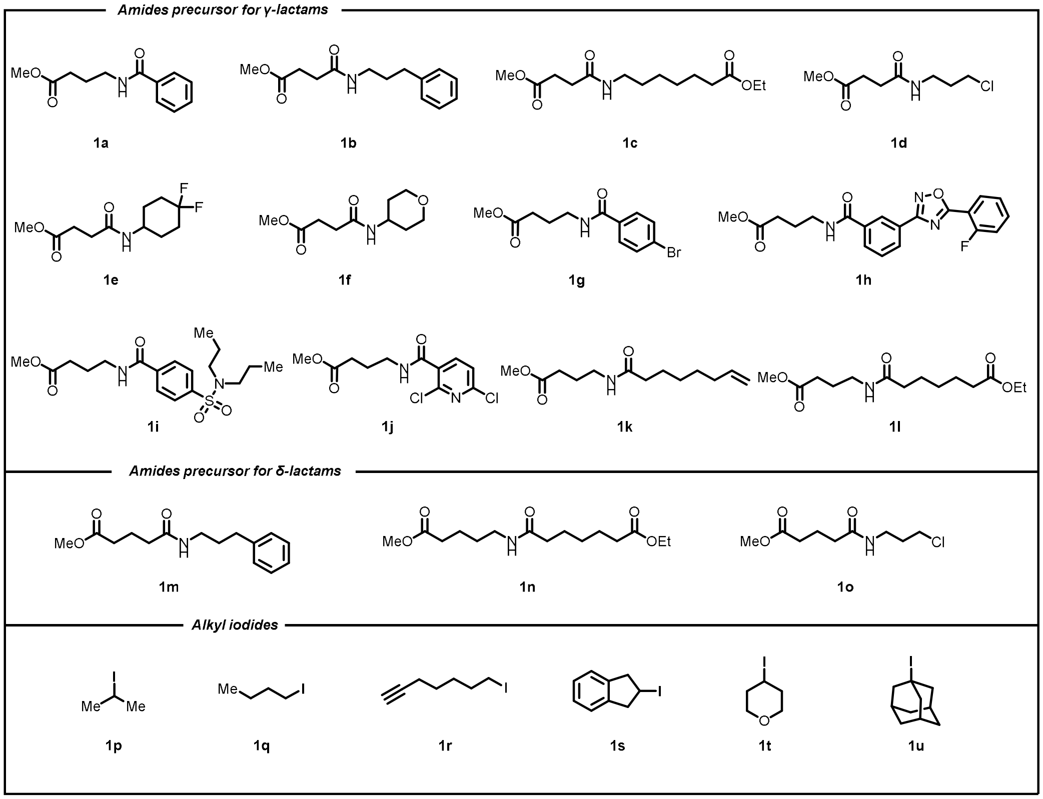

4. Synthesis of Starting Materials

4.1. General procedure 1 (GP1) for the preparation of secondary amides from benzoyl chloride

To an oven-dried round-bottom flask equipped with a septum and a stirring-bar was added the amine (1.1 equiv.) and dry CH_2Cl_2 (0.2 M), under N_2 atmosphere. The solution was cooled to 0 °C and triethylamine (1.5 equiv.) was added. Then, benzoyl chloride (1.5-30 mmol, 1.0 equiv.) was slowly added dropwise via a syringe and the reaction was then warmed up to room temperature and stirred overnight. The reaction mixture was quenched by addition of a saturated aqueous Na_2CO_3 solution until pH~10-11 and then diluted with CH_2Cl_2. The mixture was transferred to a separation funnel and extracted with CH_2Cl_2 (2x). The combined organic layers were washed with 1 M HCl (2x) and aq. NaCl sat. (1x). The final organic layers were dried over Na_2SO_4, filtered and the solvent was removed under reduced pressure to afford the desired secondary amide. For all newly reported secondary amides see section 11.1 for characterization data.

4.2. General procedure 2 (GP2) for the preparation of secondary amides from carboxylic acids

To a stirring solution of carboxylic acid (1.5-20 mmol, 1.0 equiv.) in dry CH_2Cl_2 (0.2 M), oxalyl chloride (1.2 equiv.) and few drops of dry DMF were added dropwise at 0 °C under N_2 atmosphere. Then, the reaction mixture was stirred for 2 h at room temperature. Upon completion, the solvent was removed under reduced pressure to afford the crude carbonyl chloride. The residue was dissolved in dry CH_2Cl_2 (0.2 M), and the amine (1.1 equiv.) was added, followed by triethylamine (2 equiv.) at 0 °C under N_2 atmosphere. Then, the reaction mixture was stirred overnight at room temperature. The reaction mixture was quenched by addition of a saturated aqueous Na_2CO_3 solution until pH~10-11 and then diluted with CH_2Cl_2. The mixture was transferred to a separation funnel and extracted with CH_2Cl_2 (2x). The combined organic layers were washed with 1 M HCl (2x) and aq. NaCl sat. (1x). The final organic layers were dried over Na_2SO_4, filtered and the solvent was removed under reduced pressure to afford the desired secondary amide. For all newly reported secondary amides see section 11.1 for characterization data.

Note: For amide **1j** containing a pyridine functionality the HCl washes were omitted.

4.3. General Procedure 3 (GP3) for the preparation of alkyl iodides

The alkyl iodides used in the scope were synthesized adapting a procedure reported in the literature.[2]

To an oven-dried round-bottom flask equipped with a septum and a stirring-bar was added the alcohol (1.0 equiv.), PPh_3 (1.2 equiv.) and imidazole (1.2 equiv.) under N_2 atmosphere. CH_2Cl_2 (0.1 M) was added, and the reaction was cooled to 0 °C. I_2 (1.2 equiv.) was added portion-wise. The reaction was stirred overnight at room temperature and then diluted with a saturated aqueous $Na_2S_2O_3$ solution. The layers were separated, and the aqueous layer was extracted with CH_2Cl_2 (x3). The combined organic layers were dried over Na_2SO_4, filtered and the solvent was removed under reduced pressure. Purification by flash column chromatography on silica gel afforded the desired alkyl iodide. Alkyl iodides **1p**, **1q**, **1t** and **1u** are commercially available.

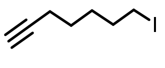

7-Iodohept-1-yne **(1r).** Prepared according to **GP3** from hept-6-yn-1-ol (503 µL, 4.00 mmol, 1.0 equiv.). Purified via flash column chromatography on silica gel (Heptane) to afford the product as a colorless oil (826 mg, 91%).

^{1}H NMR (300 MHz, $CDCl_3$) δ 3.19 (t, *J* = 7.0 Hz, 2H), 2.26 – 2.16 (m, 2H), 1.95 (t, *J* = 2.7 Hz, 1H), 1.91 – 1.80 (m, 2H), 1.63 – 1.46 (m, 4H).

The obtained data are in accordance with the literature.[3]

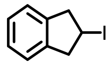

2-Iodo-2,3-dihydro-1H-indene **(1s).** Prepared according to **GP3** from 2,3-dihydro-1H-inden-2-ol (268 mg, 2.00 mmol, 1.0 equiv.). Purified via flash column chromatography on silica gel (Heptane) to afford the product as a colorless oil (332 mg, 68%).

^{1}H NMR (300 MHz, $CDCl_3$) δ 7.29 – 7.17 (m, 4H), 4.70 (tt, *J* = 6.4, 5.1 Hz, 1H), 3.58 – 3.28 (m, 4H).

The obtained data are in accordance with the literature.[4]

5. General procedure 4 (GP4): Deoxygenative alkylation of amides and lactamization (activation at 0 ˚C)

In a typical experiment, to an oven-dried 7 mL vial equipped with a stirring bar was added the corresponding amide (0.25 mmol, 1.0 equiv.) and the vial was sealed with a septum. Subsequently, dry CH_3CN (1.0 mL) was added under N_2 atmosphere (0.25 M). The mixture was cooled to 0 ˚C and 2-fluoropyridine (29 mg, 26 µl, 0.30 mmol, 1.2 equiv.) was added. Triflic anhydride (78 mg, 47 µl, 0.28 mmol, 1.1 equiv.) was added slowly dropwise and the mixture was stirred (900 rpm) for 20 minutes at 0 ˚C. Triethylsilane (32 mg, 44 µl, 0.28 mmol, 1.1 equiv.) was added dropwise and the resulting mixture was stirred at 0 ˚C for additional 10 minutes. Then, the vial was removed from the ice-water bath and left stirring at room temperature for 2 h.

After, the solution was diluted with acetonitrile (4 mL, 0.05 M *final concentration*) and the corresponding alkyl iodide (0.75 mmol, 3.0 equiv.) and tris(trimethylsilyl)silane (124 mg, 154 µl, 0.50 mmol, 2.0 equiv.) were added.

For the reactions performed under continuous flow conditions: The solution was then transferred under N_2 atmosphere to a 10 mL gastight syringe. The syringe was mounted on a syringe pump and pushed into the Signify "Eagle Reactor" (See section 2.2). The flow rate is set to achieve 20 minutes residence time (reactor volume 2.8 mL, flow rate 0.14 mL/min). Once the syringe containing the reaction solution was fully emptied a second 10 mL gastight syringe containing CH_3CN was loaded to push the reaction solution out of the reactor. The outflow was collected in a 20 mL amber vial.

For the reactions performed under batch conditions: The vial containing the solution was sealed with electrical tape and stirred and irradiated under 390 nm in the UFO photochemical reactor (See section 2.4) for 48 h. The temperature was maintained at 30-35 ˚C during the course of the reaction.

Finally, the solutions obtained from the photochemical reactors were evaporated under reduced pressure. The crude was suspended in *n*-pentane (10 mL) and sonicated for two minutes. The surnatant was filtered through a plug of celite to retain solid traces. This process was repeated three times. Finally, the celite plug was washed with CH_2Cl_2 (3 × 5 mL). The CH_2Cl_2 phases were collected and added to the residual solid. The corresponding solution was then diluted with 15 mL of $NaHCO_3$ (~ 1:1 ratio organic:water phase) and the biphasic mixture was stirred for 15 minutes at rt. The solution was then transferred to a separatory funnel and extracted with CH_2Cl_2 (3 × 15 mL). The combined organic layers were dried over Na_2SO_4 and filtered, and the solvent was removed under reduced pressure. The crude reaction mixture was purified by flash column chromatography on silica gel.

6. General procedure 5 (GP5): Deoxygenative alkylation of amides and lactamization (activation at -78 ˚C)

To an oven-dried 7 mL vial equipped with a stirring bar was added the corresponding amide (0.25 mmol, 1.0 equiv.) and the vial was sealed with a septum. Subsequently, dry CH_2Cl_2 (1.0 mL) was added under N_2 atmosphere (0.25 M). The mixture was cooled to -78 ˚C and 2-fluoropyridine (29 mg, 26 µl, 0.30 mmol, 1.2 equiv.) was added. Triflic anhydride (78 mg, 47 µl, 0.28 mmol, 1.1 equiv.) was added slowly dropwise and the mixture was stirred (900 rpm) for 10 minutes at -78 ˚C followed by 10 minutes at 0 ˚C. Triethylsilane (32 mg, 44 µl, 0.28 mmol, 1.1 equiv.) was added dropwise and the resulting mixture was stirred at 0 ˚C for additional 10 minutes. Then, the vial was removed from the ice-water bath and left stirring at room temperature for 2 h. The reaction was then treated as in **GP4**.

7. Reaction optimization

7.1. Iminium ion Int-A formation in CH_3CN

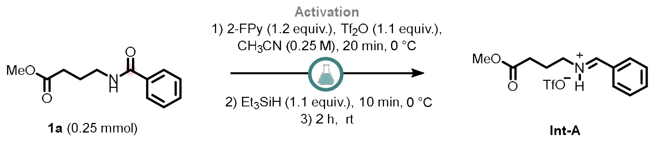

Compound **1a** was activated to generate the corresponding iminium triflate (**Int-A**) as described in **GP4**. Following activation for 2 h, the solvent was removed under reduced pressure and the crude was dissolved in $CDCl_3$, trichloroethylene was added as external standard and ^{1}H NMR was recorded to monitor the formation of iminium triflate **int-A**.

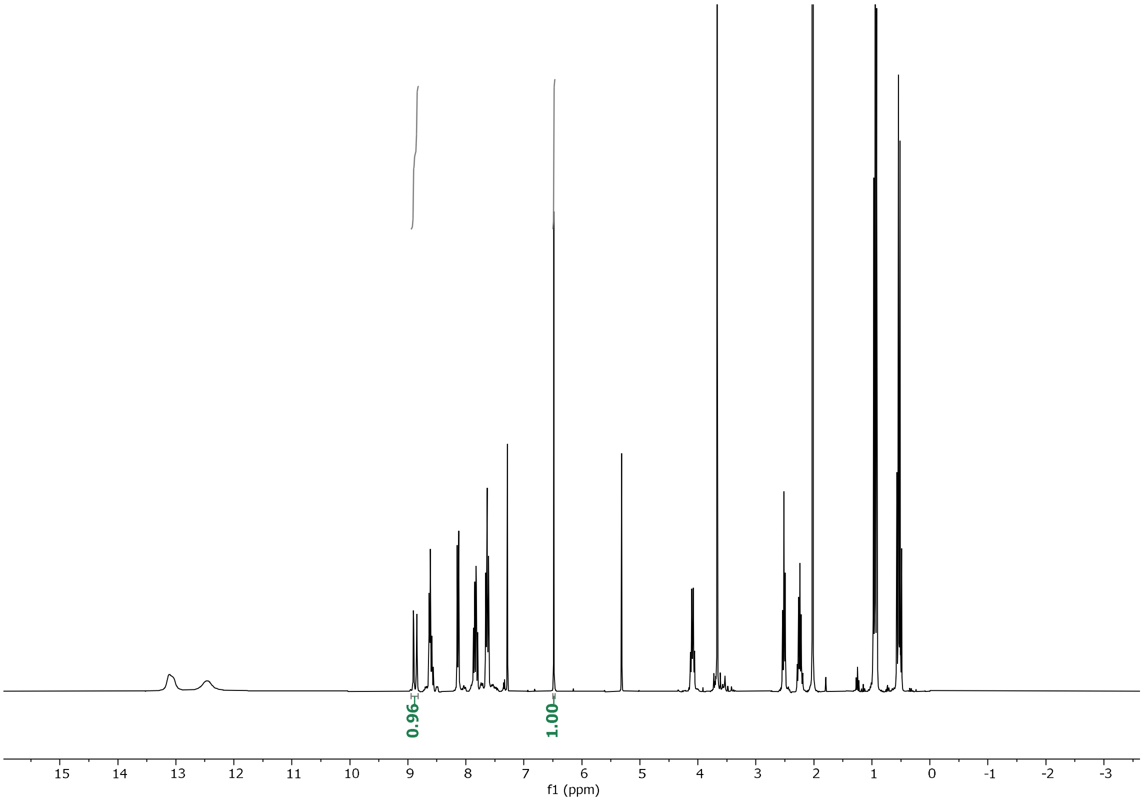

Supplementary Figure 4:Activation of **1a** in CH_3CN, 1.0 equiv. of trichloroethylene added as external standard ($\delta_{standard}$: 6.53 ppm). Formation of the iminium triflate **Int-A** observed in 96% yield.

7.2. Preliminary results in batch: effect of the basic work-up and selection of the model substrate

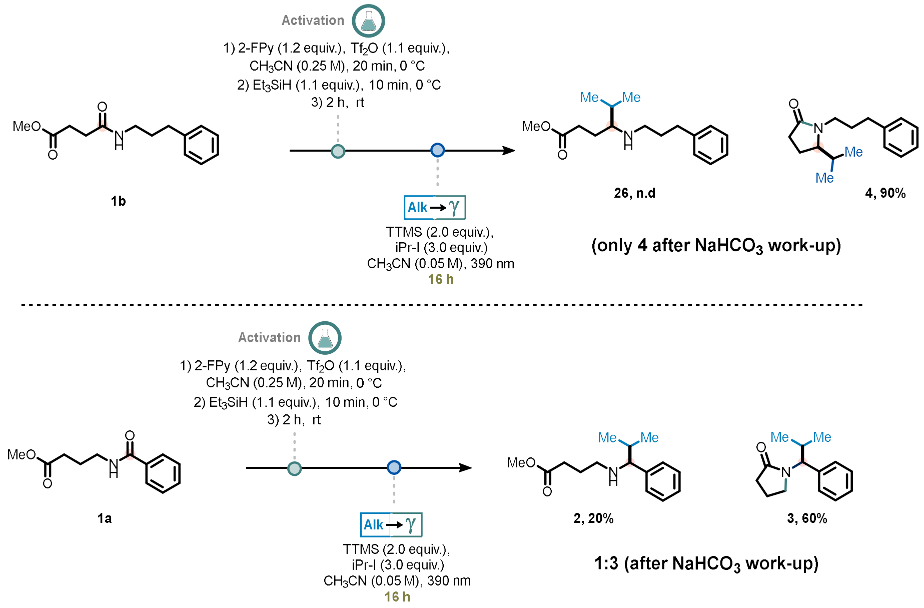

Preliminary results revealed that when subjecting amide **1b** under the reaction conditions reported in **GP4** in batch, the formation of the desired γ-substituted γ-lactam (**4**) was formed in 90% ^{1}H NMR yield with no traces of the linear alkylated analogue **26**. On the other hand, when employing amide **1a** under the same reaction conditions, a mixture **(1:3)** of the linear substituted γ-amino acid (**2**) and the desired *N*-alkylated γ-lactam (**3**) was obtained. These results motivated us to pursue the optimization campaign using amide **1a** due to the challenging and incomplete cyclization (lactamization) of the linear substituted γ-amino ester **2**.

7.3. Reaction optimization in batch

For the optimization campaign all the reactions reported below were performed in accordance to **GP4**, but omitting the $NaHCO_3$ work-up, unless otherwise stated.

Supplementary Table 1: Screening of reaction time

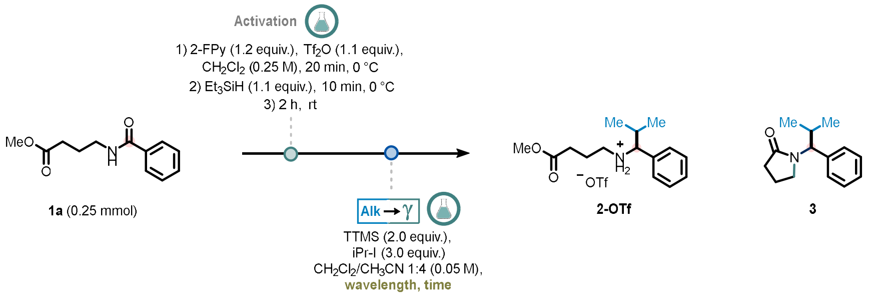

Wavelength (nm)	Time (h)	**3:2-OTf**[a]	Combined yield[a]
390	16	3:1	80%
390	24	6:1	75%
390	48	7:1	80%
390	48	7:1	80%[b]
370	48	7:1	77%[b]

[a] Determined via ^1H NMR using trichloroethylene as external standard.
[b] Activation of amide in CH_3CN instead of CH_2Cl_2

Supplementary Table 2: Optimization of stoichiometry and concentration

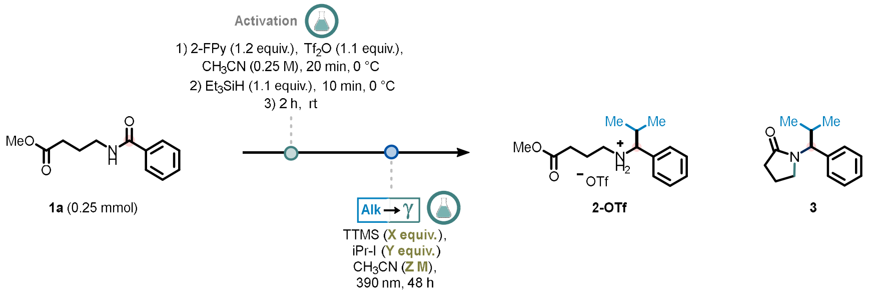

TTMS equiv.	iPr-I equiv.	Concentration	**3:2-OTf**[a]	Combined yield[a]
1.1	1.5	0.25 M	7:1	45%
2.0	3.0	0.25 M	7:1	32%
2.0	3.0	0.05 M	7:1	80%
2.0	1.5	0.05 M	1:1.5	45%
1.1	1.5	0.05 M	2.5:1	70%
1.1	3.0	0.05 M	4:1	79%
1.1	6.0	0.05 M	2:1	74%

[a] Determined via ^1H NMR using trichloroethylene as external standard.

7.4. Test of the generality of the reaction conditions in batch

Here we tested the generality of the developed reaction conditions in batch according to **GP4**. We first analyzed the reaction mixtures prior to basic work-up ($NaHCO_3$) in order to evaluate the efficiency of photochemical cascade without the influence of the base ($NaHCO_3$ work up was omitted).

Supplementary Table 3: Test of the generality of the reaction conditions

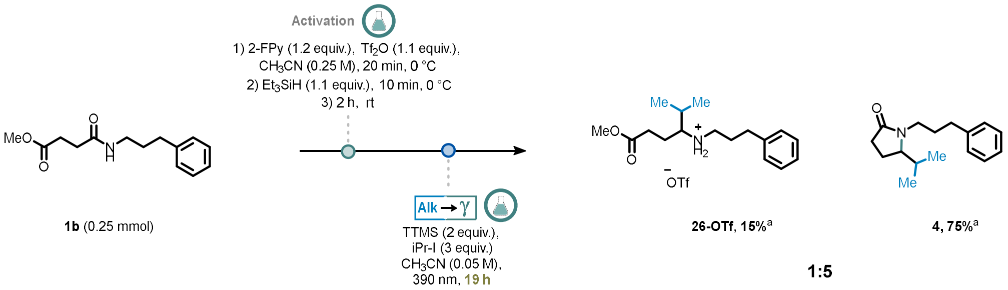

[a] Determined via ^{1}H NMR using trichloroethylene as external standard.

N.B: Fully **4** (90%) after $NaHCO_3$ wash (standard workup)

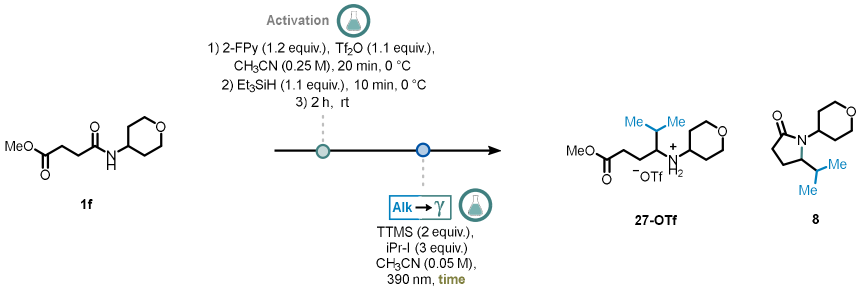

Time (h)	**8:27-OTf**[a]	Combined yield[a]
19	1:1	80%
48	5:1	65%

[a] Determined via ^{1}H NMR using trichloroethylene as external standard.

N.B: Ratio **8**:**27-OTf** unchanged after $NaHCO_3$ wash (standard workup after 19 h)

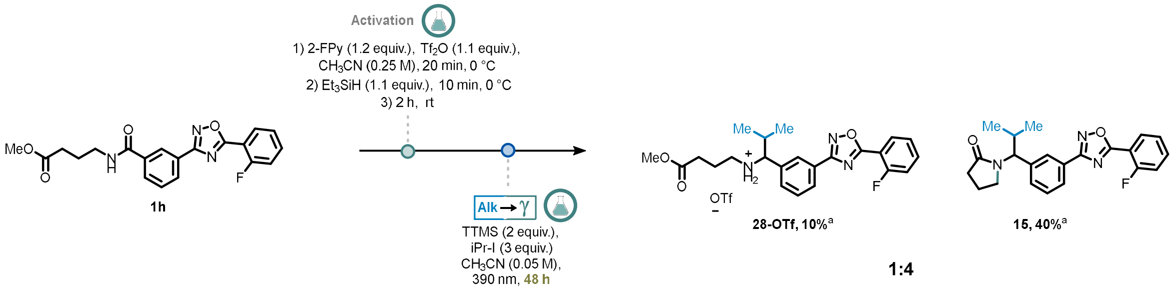

[a] Determined via ^{1}H NMR using trichloroethylene as external standard.

7.5. Translation from batch to flow

Supplementary Table 4: Effect of the back pressure regulator (BPR)

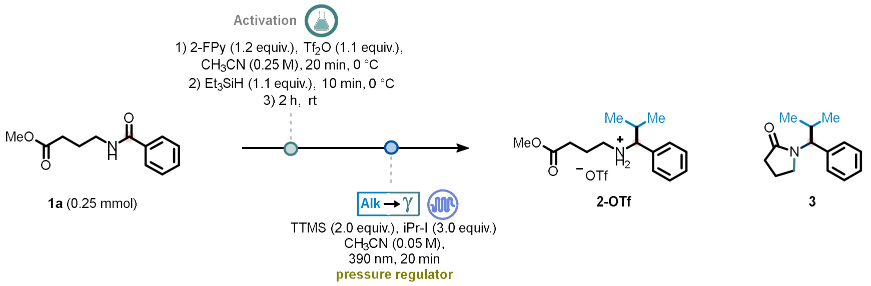

Conditions	3:2-OTf	NMR Yield[a]
no BPR (atmospheric pressure)	1:1	65%
no BPR[b] (atmospheric pressure)	1.5:1	67%
Back pressure regulator (5 bar)	only **3**	73%
Back pressure regulator (5 bar)[b]	only **3**	75%

[a]Combined yield of **2** + **3.** Determined via ^{1}H NMR using trichloroethylene as external standard.

[b]The flow solution was directly collected into a round-bottom flask containing sat. aq. $NaHCO_3$ (neutralization)

Supplementary Table 5: Translation from batch to flow

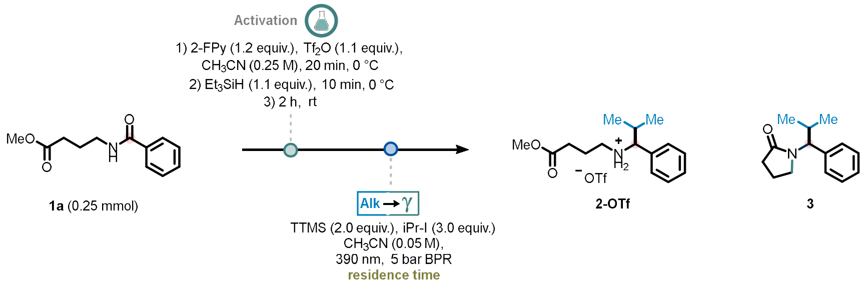

residence time	3:2-OTf[a]	NMR Yield[a]
2 min	1:25	78%
15 min	6:1	80%
20 min	Only **3**	75%

[a] Determined via ^{1}H NMR using trichloroethylene as external standard.

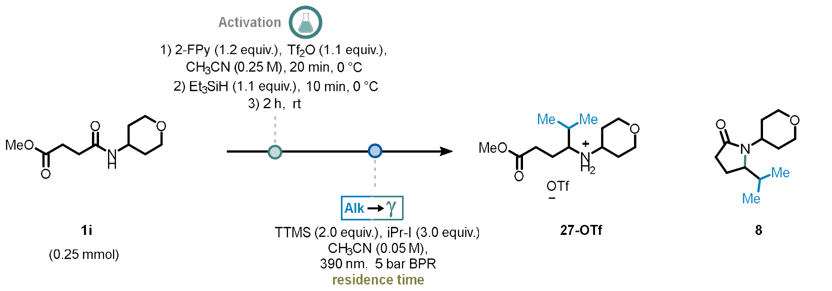

residence time	8:27-OTf[a]	NMR Yield[a]
15 min	4:1	70%
20 min	Only **8**	60%

[a] Determined via ^{1}H NMR using trichloroethylene as external standard.

7.6. Vapourtec Optimization (Effect of different set-up and light intensity)

Supplementary Table 6: Optimization residence time on Vapourtec

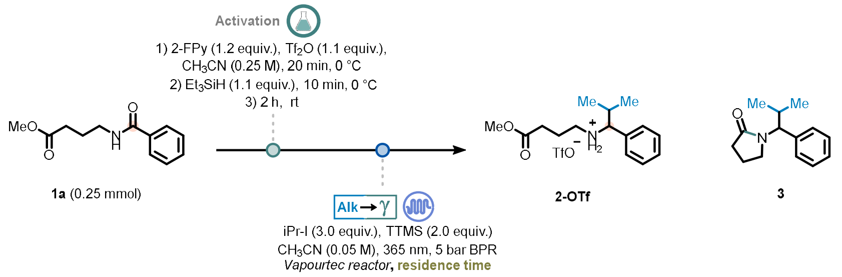

τ (min)	NMR Yield **2-OTf**	NMR Yield **3**
10	92%	0%
30	82%	11%
60	28%	30%
120	traces	60%

8. Scale up

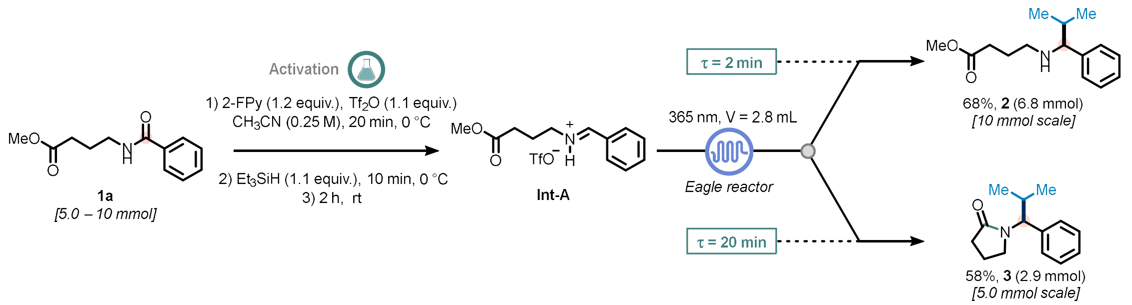

*For lactam **3***: To an oven dried 100 mL round bottom flask equipped with a stirring bar was added the corresponding amide **1a** (1.25 g, 5 mmol, 1.0 equiv.) and the round bottom flask was sealed with a septum. Subsequently, dry and degassed acetonitrile (20 mL) was added under N_2 atmosphere (0.25 M). The mixture was cooled at 0 ˚C with an ice-water bath and 2-fluoropyridine (583 mg, 516 µL, 6 mmol, 1.2 equiv.) was added. Triflic anhydride (1.55 g, 920 µL, 5.5 mmol, 1.1 equiv.) was added slowly dropwise and the mixture was stirred (900 rpm) for 20 minutes at 0 ˚C. Triethylsilane (640 mg, 880 µL, 5.5 mmol, 1.1 equiv.) was added dropwise and the resulting mixture was stirred at 0 ˚C for additional 10 minutes. Then, the round bottom flask was removed from the ice-water bath and left stirring at room temperature for 2 h. After dilution with dry and degassed acetonitrile (80 mL), the isopropyl iodide (2.6 g, 1.5 mL, 15 mmol, 3.0 equiv.) and tris(trimethylsilyl)silane (2.4 g, 2.9 mL, 10 mmol, 2.0 equiv.) were directly added in the reaction flask. Next, the final solution was pumped using 50 mL syringes into the Signify photoreactor (V = 2.8 mL) equipped with UV LEDs (λ = 365 nm, 144 W) using a syringe pump with a flow rate of 0.14 mL·min^{-1} (τ = 20 min). The outflow was collected in a 250 mL round-bottom flask. After all reaction mixture had been pumped through the reactor, 6 mL of acetonitrile were loaded into the reaction flask and pushed into the reactor to collect all the product. Finally, the solvent was evaporated under reduced pressure. The crude was suspended in *n*-pentane and sonicated for two minutes. The surnatant was filtered through a plug of celite to retain solid traces. This process was repeated three times. Finally, the celite plug was washed three times with CH_2Cl_2. The CH_2Cl_2 phases were collected and added to the residual solid. The corresponding solution was then diluted with $NaHCO_3$ (~1:1 ratio organic:water phase) and the biphasic mixture was stirred for 15 minutes at rt. The solution was then transferred to a separatory funnel and extracted three times with CH_2Cl_2. The combined organic layers were dried over Na_2SO_4, filtered and the solvent was removed under reduced pressure. The residue was purified via flash column chromatography on silica gel (Pentane:Ethyl Acetate 1:1) to afford the product as a colorless oil (0.63 g, 58% yield).

*For α-alkylatyed secondary amine **2***: To an oven dried 100 mL round bottom flask equipped with a stirring bar was added the corresponding amide **1a** (2.2 g, 10 mmol, 1.0 equiv.) and the round bottom flask was sealed with a septum. Subsequently, dry and degassed acetonitrile (40 mL) was added under N_2 atmosphere (0.25 M). The mixture was cooled at 0 ˚C with an ice-water bath and 2-fluoropyridine (1.2 g, 1.0 mL, 12 mmol, 1.2 equiv.) was added. Triflic anhydride (3.1 g, 1.9 mL, 11 mmol, 1.1 equiv.) was added slowly dropwise and the mixture was stirred (900 rpm) for 20 minutes at 0 ˚C. Triethylsilane (1.3 g, 1.8 mL, 11 mmol, 1.1 equiv.) was added dropwise and the resulting mixture was stirred at 0 ˚C for additional 10 minutes. Then, the round bottom flask was removed from the ice-water bath and left stirring at room temperature for 2 h. After dilution with dry and degassed acetonitrile (160 mL), the isopropyl iodide (5.1 g, 3.0 mL, 30 mmol, 3.0 equiv.) and tris(trimethylsilyl)silane (5.0 g, 6.2 mL, 20 mmol, 2.0 equiv.) were directly added in the reaction flask. Next, the final solution was pumped using 50 mL syringes into the Signify photoreactor (V = 2.8 mL) equipped with UV LEDs (λ = 365 nm, 144 W) using a syringe pump with a flow rate of 1.4 mL·min^{-1} (τ = 2 min). The outflow was collected in a 500 mL round-bottom flask. After all reaction mixture had been pumped through the reactor, 6 mL of

acetonitrile were loaded into the reaction flask and pushed into the reactor to collect all the product. Finally, the solvent was evaporated under reduced pressure. The crude was suspended in *n*-pentane and sonicated for two minutes. The surnatant was filtered through a plug of celite to retain solid traces. This process was repeated three times. Finally, the celite plug was washed three times with CH_2Cl_2. The CH_2Cl_2 phases were collected and added to the residual solid. The corresponding solution was then diluted with $NaHCO_3$ (~ 1:1 ratio organic:water phase) and the biphasic mixture was stirred for 15 minutes at rt. The solution was then transferred to a separatory funnel and extracted three times with CH_2Cl_2. The combined organic layers were dried over Na_2SO_4, filtered and the solvent was removed under reduced pressure. The residue was purified via flash column chromatography on silica gel (Pentane:Ethyl Acetate 1:1) to afford the product as a colorless oil (1.7 g, 68% yield).

^{1}H NMR (300 MHz, $CDCl_3$) δ 7.34 – 7.27 (m, 2H), 7.25 – 7.18 (m, 3H), 3.64 (s, 3H), 3.29 (d, *J* = 6.9 Hz, 1H), 2.51 – 2.21 (m, 4H), 1.91 – 1.67 (m, 3H), 1.31 – 1.18 (m, 1H), 0.95 (d, *J* = 6.7 Hz, 3H), 0.73 (d, *J* = 6.8 Hz, 3H).
^{13}C NMR (75 MHz, $CDCl_3$) δ 174.3, 142.9, 128.0, 127.9, 126.7, 127.0, 69.5, 51.49, 34.4, 32.0, 25.4, 19.7, 19.4
HRMS (FD+) (m/z): $[M]^{+\cdot}$ calcd. for $C_{15}H_{23}NO_2^{+\cdot}$, 249.1723; found: 249.1744.

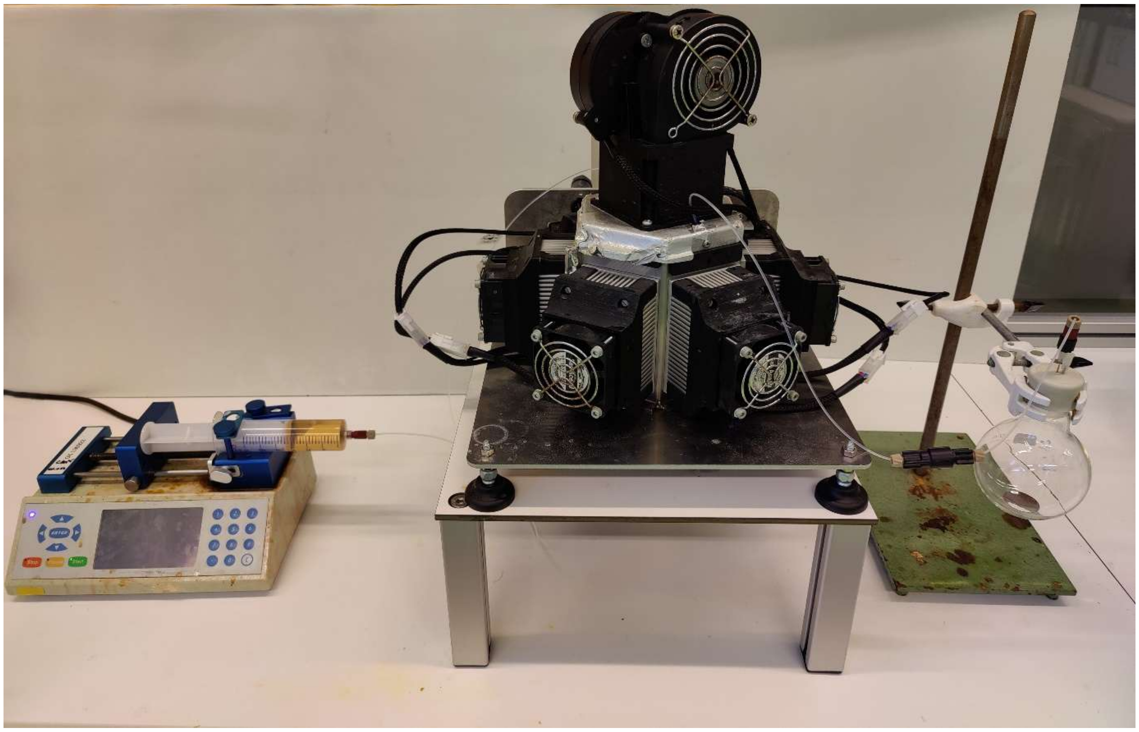

Supplementary Figure 5: Set-up employed for the scale-up reactions of linear (**2**) and cyclized (**3**).

9. Mechanistic investigation

9.1. Reaction kinetics of the flow cascade (alkylation + cyclization)

Aim of the experiment: To understand if the alkylation and the ring closure to form the lactam occur concurrently or as consecutive reactions, a kinetic experiment was performed. **GP4** was followed while varying the residence time by adjusting the flow rate accordingly.

Supplementary Table 7: Kinetic study

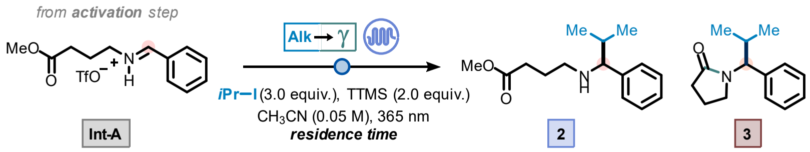

τ (min)	Int-A[a]	2[a]	3[a]
0	90%	–	–
0.5	68%	11%	–
1	49%	33%	–
2	–	75%	3%
5	–	50%	14%
10	–	32%	38%
12	–	22%	48%
15	–	10%	65%
20	–	–	75%

[a] Determined via ^{1}H NMR using trichloroethyleneas external standard

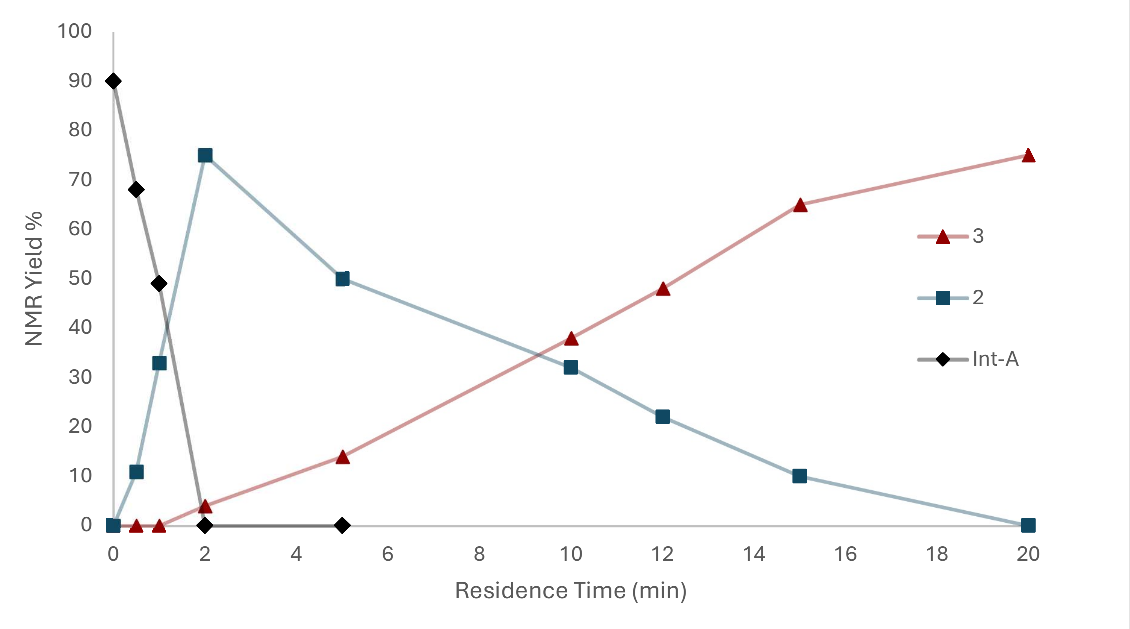

9.2. Lactamization screening under thermal conditions in batch

Product **2** was prepared following **GP4**, reducing the residence time from 20 minutes to 2 minutes. The product was then used for the follow-up experiments without column purification (See below typical ^{1}H NMR of the crude **2**). To an oven-dried 7 mL vial equipped with a stirring bar were added solvents and reagents as described in **Supplementary Table 8**.

Supplementary Table 8: Lactamization screening under thermal conditions

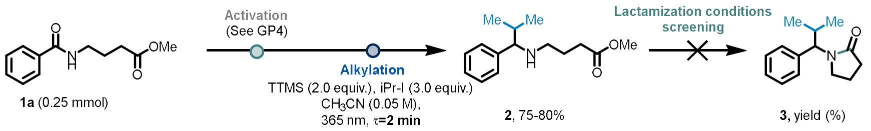

Lactamization conditions	Yield **2**[a]	Yield **3**[a]
After pentane wash	75%[b]	2%
$NaHCO_{3\ (aq)}$ work up	75%	2%
DMF (0.1 M), 130 °C, 16 h	60%	n.d.
DIPEA (1 equiv.), DMF (0.1 M), 130 °C, 16 h	50%	3%
$NaHCO_3$ (2 equiv.) CH_3CN, 80 °C, 2 h	55%	2%
$AlCl_3$ (1 equiv.), CH_3CN (0.1 M), 80 °C, 16 h	63%	<1%

[a]NMR yield. [b]Obtained as ammonium triflate. $NaHCO_3$ (sat) work up describe in GP4 was omitted

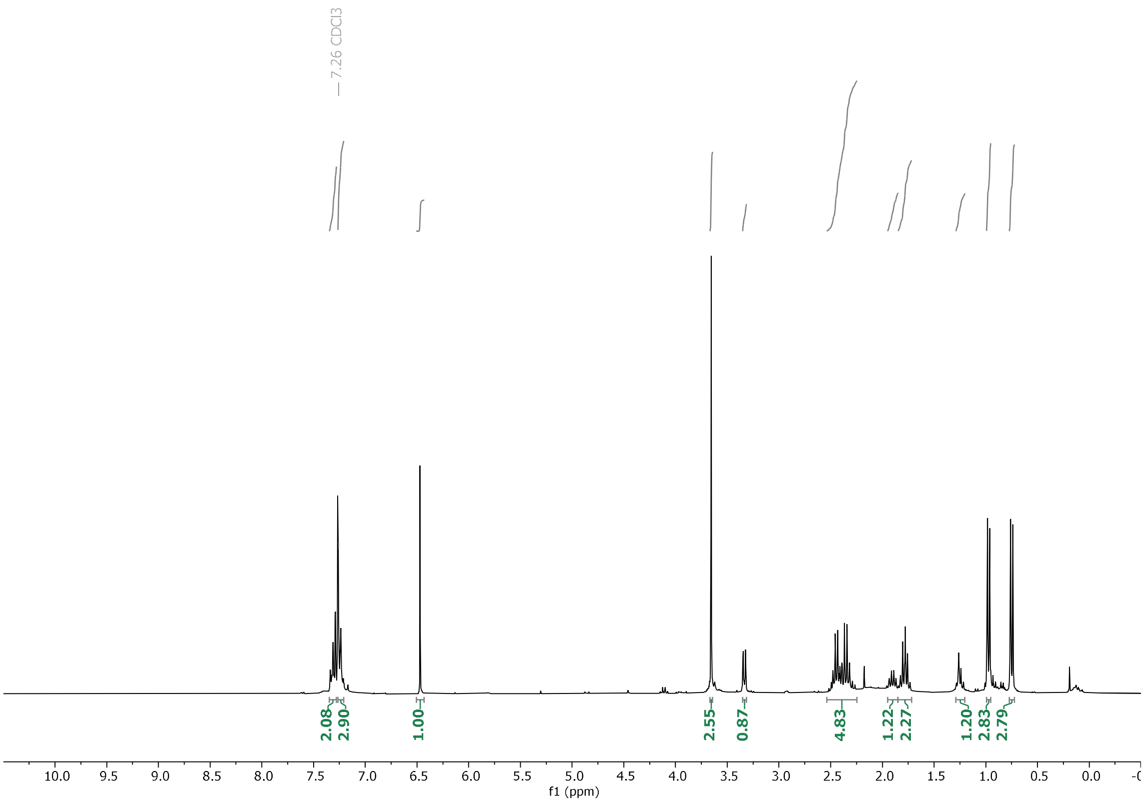

Supplementary Figure 6: Example of ^{1}H NMR of the reaction crude containing **2** (before lactamization) after pentane wash and $NaHCO_3$ (sat) work-up.

9.3. Lactamization studies from linear α-branched amine (2)

9.3.1. Influence of the activation conditions mixture on the lactamization of 2-OTf

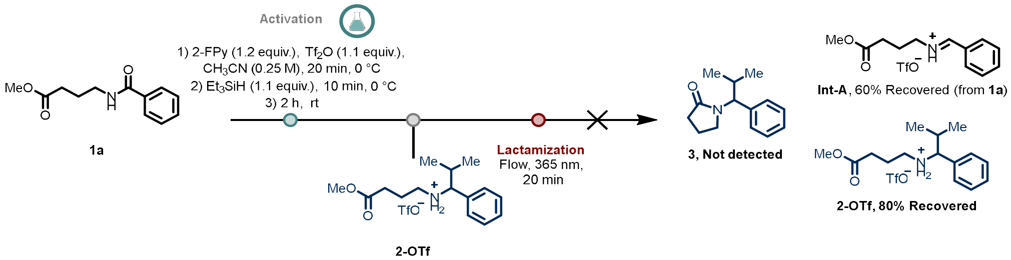

Aim of the experiment: To exclude the role of triethylsilane derived species obtained after the activation step in the promotion of the *Lactamization step*, we performed the experiment graphically described above: the linear α-branched ammonium triflate **2-OTf** was added to the reaction solution obtained after the activation step (containing **Int-A**) and then the new solution was irradiated under 365 nm. Since no lactam formation was observed, the triethylsilane derived species and 2-fluoropyridine obtained in the activation step alone are not sufficient to induce the lactam formation.

Procedure: To an oven-dried 7 mL vial equipped with a stirring bar was added **1** (0.125 mmol, 1.0 equiv.) and the vial was sealed with a septum. Subsequently, dry CH_3CN (0.5 mL) was added under N_2 atmosphere (0.25 M). The mixture was cooled at 0 ˚C with an ice-water bath and 2-fluoropyridine (15 mg, 13 µl, 0.15 mmol, 1.2 equiv.) was added. Triflic anhydride (39 mg, 24 µl, 0.14 mmol, 1.1 equiv.) was added slowly dropwise and the mixture was stirred (900 rpm) for 20 minutes at 0 ˚C. Triethylsilane (16 mg, 22 µl, 0.14 mmol, 1.1 equiv.) was added dropwise and the resulting mixture was stirred at 0 ˚C for additional 10 minutes. Then, the vial was removed from the ice-water bath and left stirring at room temperature for 2 h.

To an oven-dried round bottom flask equipped with a stirring bar was added methyl 4-[(2-methyl-1-phenylpropyl)amino]butanoate **2** to obtain a 0.25 M mother solution and the flask was sealed with a septum and degassed with N_2. Subsequently, dry CH_3CN was added under N_2 atmosphere (0.25 M). The mixture was cooled at 0 ˚C with an ice-water bath and Triflic acid (1.0 equiv.) was added dropwise.

0.5 mL of the mother solution was transferred under N_2 to the vial containing the product of the activation step. The solution was diluted with acetonitrile (4 mL, 0.05 M *final concentration*) and then transferred under N_2 atmosphere in a 10 mL gastight syringe. The syringe mounted on a syringe pump was pushed into the Signify "Eagle Reactor" (See section 2.2). A 5 bar BPR was positioned at the end of the reactor. The flow rate was set to achieve 20 minutes residence time. Once the syringe containing the reaction solution is fully emptied a second 10 mL gastight syringe containing CH_3CN was loaded to push the reaction solution out of the reactor. And the outflow was collected in a 20 mL amber vial.

Finally, the solvent was evaporated under reduced pressure. The crude was suspended in *n*-pentane and sonicated for two minutes. The surnatant was filtered through a plug of celite to retain solid traces. This process was repeated three times. Finally, the celite plug was washed three times with CH_2Cl_2. The CH_2Cl_2 phases were collected and added to the residual solid. The solvent evaporated and the rection mixture was analyzed via ^1H NMR using trichloroethylene as external standard.

9.3.2. *Additives effect on the lactamization of linear α-branched amine (2)*

Aim of the experiments: To understand what are the minimum conditions that enable the cyclization, the single components used in the alkylation/cyclization steps were analyzed. Additionally, also the main side products identified were investigated.

Thermal lactamization

To an oven-dried round bottom flask equipped with a stirring bar was added methyl 4-[(2-methyl-1-phenylpropyl)amino]butanoate **2.** The flask was sealed with a septum and degassed with N_2. Subsequently, dry CH_3CN was added under N_2 atmosphere (0.25 M). The mixture was cooled at 0 ˚C with an ice-water bath and triflic acid (1.0 equiv.) was added dropwise to form **2-OTf** (90% avg. yield).

1 mL of the **2-OTf** solution was transferred under N_2 to an oven-dried 7 mL vial. The solution was diluted with acetonitrile (4 mL, 0.05 M *final concentration*) and the investigated reagents were added.

The solution was then transferred under N_2 atmosphere in a 10 mL gastight syringe. The syringe mounted on a syringe pump was pushed into a 2.8 mL coiled tube reactor (FEP capillary tubing: 1.6 mm OD, 0.8 mm ID). The coil reactor is heated using an oil bath at 55°C in which it is submerged. A 5 bar BPR is positioned at the end of the reactor. The flow rate is set to achieve 20 minutes residence time. Once the syringe containing the reaction solution is fully emptied a second 10 mL gastight syringe containing CH_3CN was loaded to push the reaction solution out of the reactor. The outflow is collected in a 20 mL amber vial.

Finally, the solvent was evaporated under reduced pressure. The crude was suspended in *n*-pentane and sonicated for two minutes. The surnatant was filtered through a plug of celite to retain solid traces. This process was repeated three times. Finally, the celite plug was washed three times with CH_2Cl_2. The CH_2Cl_2 phases were collected and added to the residual solid. The solvent evaporated and the rection mixture is analyzed via ^1H NMR using trichloroethylene as external standard.

Supplementary Table 9: Investigation of the cyclization reaction under thermal conditions

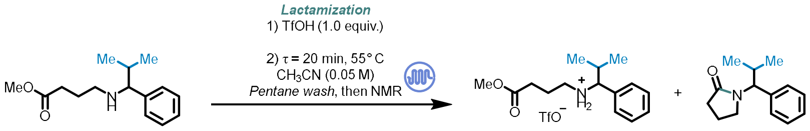

2 (0.25 mmol) **2-OTf** **3**

Reagents	3:2-OTf[a]	NMR Yield[a]
No	only **2-OTf**	~65%
TTMS-I (2.0 equiv)	only **2-OTf**	~50%
iPr-I (3.0 equiv.)	only **2-OTf**	~50%
TTMS (2.0 equiv)	only **2-OTf**	90%
TTMS (2.0 equiv) + iPr-I (3.0 equiv.)	only **2-OTf**	88%
TTMS (2.0 equiv) + iPr-I (3.0 equiv.) +2-F Py (1.2 equiv.)	only **2-OTf**	50%

[a]Combined yield of **2** + **3.** Determined via ^1H NMR using trichloroethylene as external standard.

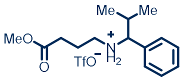

2-OTf

2-OTf formed after step 1)
(average 90% from **2** after TfOH addition)

Photochemical lactamization

To an oven-dried round bottom flask equipped with a stirring bar was added methyl 4-[(2-methyl-1-phenylpropyl)amino]butanoate **2.** The flask was sealed with a septum and degassed with N_2. Subsequently, dry CH_3CN was added under N_2 atmosphere (0.25 M). The mixture was cooled at 0 ˚C with an ice-water bath and triflic acid (1.0 equiv.) was added dropwise to form **2-OTf** (90% avg. yield).

1 mL of the **2-OTf** solution was transferred under N_2 to an oven-dried 7 mL vial. The solution was diluted with acetonitrile (4 mL, 0.05 M *final concentration*) and the investigated reagents were added.

The solution was then transferred under N_2 atmosphere in a 10 mL gastight syringe. The syringe mounted on a syringe pump was pushed into the Signify "Eagle Reactor" (See section 2.2). A 5 bar BPR is positioned at the end of the reactor. The flow rate is set to achieve 20 minutes residence time. Once the syringe containing the reaction solution is fully emptied a second 10 mL gastight syringe containing CH_3CN was loaded to push the reaction solution out of the reactor. The outflow is collected in a 20 mL amber vial.

Finally, the solvent was evaporated under reduced pressure. The crude was suspended in *n*-pentane and sonicated for two minutes. The surnatant was filtered through a plug of celite to retain solid traces. This process was repeated three times. Finally, the celite plug was washed three times with CH_2Cl_2. The CH_2Cl_2 phases were collected and added to the residual solid. The solvent evaporated and the rection mixture is analyzed via ^1H NMR using trichloroethylene as external standard.

TTMS-I [5] and *TTMS-OTf* [6] were prepared according to known procedures.

Supplementary Table 10: Investigation of the cyclization reaction under photochemical conditions

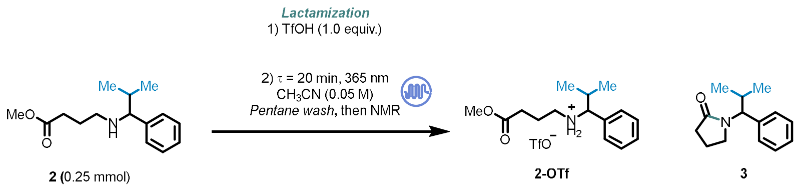

Reagents	3:2-OTf[a]	NMR Yield (2+3)
No	Only **2-OTf**	~77%
TTMS (2.0 equiv.)	Only **2-OTf**	90%
2-F Py (1.2 equiv.)	Only **2-OTf**	80%
*i*Pr-I (3.0 equiv.)	Only **2-OTf**	~90%
TTMS-I (2.0 equiv)	Only **2-OTf**	~53%
TTMS-OTf (1.0 equiv.)	Only **2-OTf**	80%
TES-OTf (1.0 equiv.)	Only **2-OTf**	80%
TTMS-I (2.0 equiv.) + 2-F Py (1.2 equiv.)	Only **2-OTf**	85%
TTMS (2.0 equiv.) + iPr-I (3.0 equiv.)	Only **3**	46%
TTMS (2.0 equiv.), iPr-I (3.0 equiv.), 2-F Py (1.2 equiv.)	Only **3**	60%
TTMS (1.0 equiv.), iPr-I (1.5 equiv.),TTMS-I (1.0 equiv.)	1.5:1	90%
TTMS (1.0 equiv.), iPr-I (1.5 equiv.)	1:2	90%

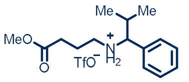

2-OTf

2-OTf formed after step 1)

(average 90% from **2** after TfOH addition)

Supplementary Table 11: Lactamization attempt of neutral intermediate **2** (no TfOH for ammonium formation)

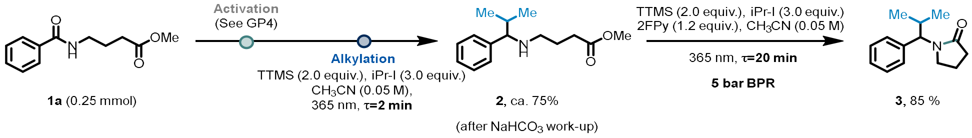

10.Limitations

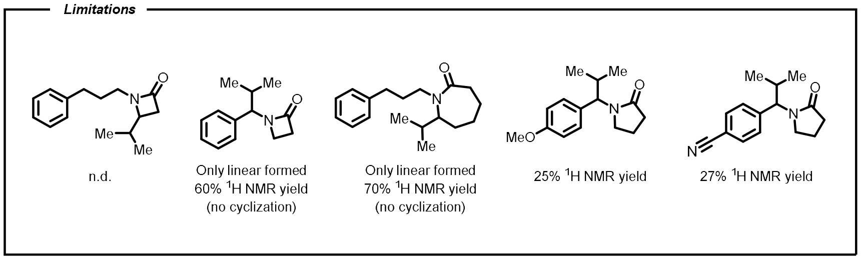

Photochemical lactamization using isopropyl bromide

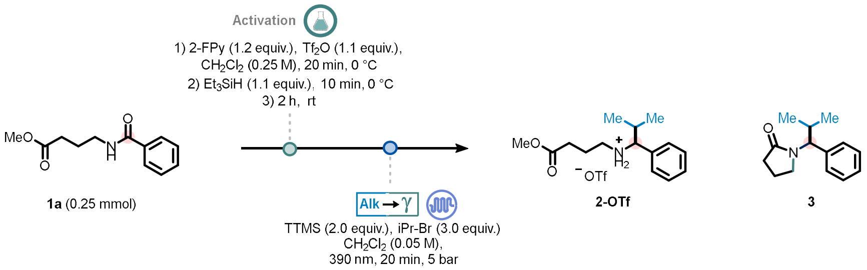

Additive	NMR Yield **3**[a]
none	nd
NaI (3 equiv.)	nd

[a]Determined via ^{1}H NMR using trichloroethylene as external standard.

11.Characterization data of synthesized compounds

11.1. Characterization of compounds 1a-1o (*secondary amides*)

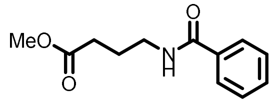

***Methyl 4-benzamidobutanoate* (1a).** Prepared according to **GP1** from benzoyl chloride (1.74 mL, 2.11 g, 15.0 mmol, 1.0 equiv.) and methyl 4-aminobutanoate hydrochloride (2.30 g, 15.0 mmol, 1.0 equiv.) to afford the product as a white solid (3.03 g, 91%).

^{1}H NMR (400 MHz, $CDCl_3$) δ 7.77 (d, *J* = 7.1 Hz, 2H), 7.48 (t, *J* = 7.3 Hz, 1H), 7.41 (t, *J* = 7.4 Hz, 2H), 6.62 (s, 1H), 3.66 (s, 3H), 3.50 (q, *J* = 6.5 Hz, 2H), 2.44 (t, *J* = 7.0 Hz, 2H), 1.96 (p, *J* = 6.9 Hz, 2H).
^{13}C NMR (101 MHz, $CDCl_3$) δ 174.4, 167.7, 134.6, 131.53, 128.7, 127.0, 51.9, 39.8, 31.9, 24.6.
HRMS (FD+) (m/z): $[M+H]^+$ calcd. for $C_{12}H_{16}NO_3^+$, 222.1125; found: 222.1130.

The obtained data are in accordance with the literature.[7]

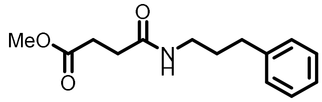

***Methyl 4-oxo-4-((3-phenylpropyl)amino)butanoate* (1b).** Prepared according to **GP2** from 4-methoxy-4-oxobutanoic acid (661 mg, 5.00 mmol, 1.0 equiv.) and 3-phenylpropan-1-amine (711 µL, 676 mg, 5.00 mmol, 1.0 equiv.) to afford the product as a white solid (1.06 g, 85%).

^{1}H NMR (400 MHz, $CDCl_3$) δ 7.31 – 7.24 (m, 2H), 7.22 – 7.14 (m, 3H), 5.72 (s, 1H), 3.67 (s, 3H), 3.28 (q, *J* = 6.8 Hz, 2H), 2.69 – 2.59 (m, 4H), 2.42 (t, *J* = 6.8 Hz, 2H), 1.83 (p, *J* = 7.3 Hz, 2H).
^{13}C NMR (101 MHz, $CDCl_3$) δ 173.7, 171.4, 141.6, 128.6, 128.5, 126.1, 51.9, 39.4, 33.4, 31.3, 31.2, 29.5.
HRMS (FD+) (m/z): $[M]^{+\cdot}$ calcd. for $C_{14}H_{19}NO_3^{+\cdot}$, 249.1359; found: 249.1361.

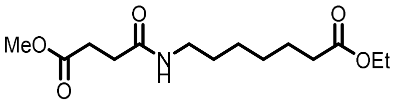

***Ethyl 7-(4-methoxy-4-oxobutanamido)heptanoate* (1c).** Prepared according to **GP2** from 4-methoxy-4-oxobutanoic acid (661 mg, 5.00 mmol, 1.0 equiv.) and ethyl 7-aminoheptanoate hydrochloride (1.05 mg, 5.00 mmol, 1.0 equiv.) to afford the product as a white solid (1.05 g, 73%).

^{1}H NMR (400 MHz, $CDCl_3$) δ 5.71 (s, 1H), 4.11 (q, *J* = 6.8 Hz, 2H), 3.67 (s, 3H), 3.22 (q, *J* = 6.8 Hz, 2H), 2.66 (t, *J* = 6.5 Hz, 2H), 2.45 (t, *J* = 6.8 Hz, 2H), 2.27 (t, *J* = 7.2 Hz, 2H), 1.61 (p, *J* = 6.9 Hz, 2H), 1.48 (p, *J* = 6.7 Hz, 2H), 1.36 – 1.28 (m, 4H), 1.24 (t, *J* = 7.1 Hz, 3H).
^{13}C NMR (101 MHz, $CDCl_3$) δ 173.9, 173.7, 171.4, 60.3, 51.9, 39.6, 34.3, 31.2, 29.5, 29.5, 28.8, 26.6, 24.9, 14.4.
HRMS (FD+) (m/z): $[M]^{+\cdot}$ calcd. for $C_{14}H_{25}NO_5^{+\cdot}$, 287.1727; found: 287.1733.

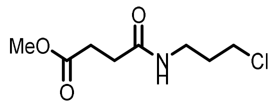

Methyl 4-((3-chloropropyl)amino)-4-oxobutanoate **(1d).** Prepared according to **GP2** from 4-methoxy-4-oxobutanoic acid (661 mg, 5.00 mmol, 1.0 equiv.) and 3-chloropropan-1-amine hydrochloride (650 mg, 5.00 mmol, 1.0 equiv.). Purified by column chromatography (Pentane:Ethyl acetate 90:10 to 25:75) to afford the product as a clear oil (417 mg, 40%).

^{1}H NMR (400 MHz, $CDCl_3$) δ 5.87 (s, 1H), 3.68 (s, 3H), 3.58 (t, *J* = 6.3 Hz, 2H), 3.41 (q, *J* = 6.4 Hz, 2H), 2.67 (t, *J* = 6.7 Hz, 2H), 2.46 (t, *J* = 6.7 Hz, 2H), 1.99 (p, *J* = 6.4 Hz, 2H).
^{13}C NMR (101 MHz, $CDCl_3$) δ 173.6, 171.8, 52.0, 42.6, 37.2, 32.2, 31.2, 29.5.
HRMS (FD+) (m/z): $[M]^{+\cdot}$ calcd. for $C_8H_{14}ClNO_3^{+\cdot}$, 207.0657; found: 207.0662.

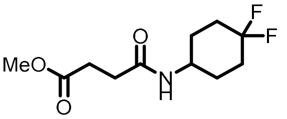

Methyl 4-((4,4-difluorocyclohexyl)amino)-4-oxobutanoate **(1e).** Prepared according to **GP2** from 4-methoxy-4-oxobutanoic acid (661 mg, 5.00 mmol, 1.0 equiv.) and 4,4-difluorocyclohexan-1-amine hydrochloride (858 mg, 5.00 mmol, 1.0 equiv.) to afford the product as an off-white solid (928 mg, 75%).

^{1}H NMR (300 MHz, $CDCl_3$) δ 5.71 (d, *J* = 7.6 Hz, 1H), 3.96 – 3.78 (m, 1H), 3.68 (s, 3H), 2.66 (t, *J* = 6.9 Hz, 2H), 2.44 (t, *J* = 6.9 Hz, 2H), 2.21 – 1.67 (m, 6H), 1.61 – 1.40 (m, 2H).
^{13}C NMR (101 MHz, $CDCl_3$) δ 173.6, 171.1, 122.6 (dd, *J* = 241.3, 241.1 Hz), 52.0 (d, *J* = 2.8 Hz), 46.3, 32.2 (t, *J* = 24.8 Hz), 31.2, 29.5, 28.6 (d, *J* = 9.0 Hz).
^{19}F NMR (282 MHz, $CDCl_3$) δ -94.99 (d, *J* = 237.6 Hz), -101.09 (d, *J* = 237.8 Hz).
HRMS (FD+) (m/z): $[M]^{+\cdot}$ calcd. for $C_{11}H_{17}F_2NO_3^{+\cdot}$, 249.1171; found: 249.1170.

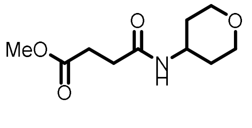

Methyl 4-oxo-4-((tetrahydro-2*H*-pyran-4-yl)amino)butanoate **(1f).** Prepared according to **GP2** from 4-methoxy-4-oxobutanoic acid (661 mg, 5.00 mmol, 1.0 equiv.) and tetrahydro-2*H*-pyran-4-amine (518 µL, 506 mg, 5.00 mmol, 1.0 equiv.) to afford the product as a white solid (390 mg, 36%).

^{1}H NMR (400 MHz, $CDCl_3$) δ 5.59 (s, 1H), 4.05 – 3.89 (m, 3H), 3.68 (s, 3H), 3.46 (t, *J* = 11.6 Hz, 2H), 2.67 (t, *J* = 7.0 Hz, 2H), 2.45 (t, *J* = 6.7 Hz, 2H), 1.92 – 1.84 (m, 2H), 1.51 – 1.40 (m, 2H).
^{13}C NMR (101 MHz, $CDCl_3$) δ 173.6, 170.8, 66.9, 52.0, 45.9, 33.2, 31.3, 29.5.
HRMS (GC-FI+) (m/z): $[M]^{+\cdot}$ calcd. for $C_{10}H_{17}NO_4^{+\cdot}$, 215.1158; found: 215.1153.

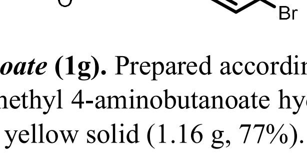

Methyl 4-(4-bromobenzamido)butanoate **(1g).** Prepared according to **GP2** from 4-bromobenzoic acid (1.01 g, 5.00 mmol, 1.0 equiv.) and methyl 4-aminobutanoate hydrochloride (768 mg, 5.00 mmol, 1.0 equiv.) to afford the product as a pale yellow solid (1.16 g, 77%).

^{1}H NMR (300 MHz, $CDCl_3$) δ 7.66 (d, *J* = 8.7 Hz, 2H), 7.57 (d, *J* = 8.6 Hz, 2H), 6.61 (s, 1H), 3.67 (s, 3H), 3.50 (q, *J* = 6.5 Hz, 2H), 2.46 (t, *J* = 6.8 Hz, 2H), 1.97 (p, *J* = 6.8 Hz, 2H).
^{13}C NMR (101 MHz, $CDCl_3$) δ 174.5, 166.6, 133.4, 131.9, 128.7, 126.2, 52.0, 40.0, 32.0, 24.3.
HRMS (GC-FI+) (m/z): $[M]^{+\cdot}$ calcd. for $C_{12}H_{14}BrNO_3^{+\cdot}$, 299.0152; found: 299.0145.

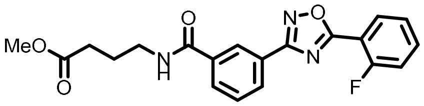

***Methyl 4-(3-(5-(2-fluorophenyl)-1,2,4-oxadiazol-3-yl)benzamido)butanoate* (1h).** Prepared according to **GP2** from 3-(5-(2-fluorophenyl)-1,2,4-oxadiazol-3-yl)benzoic acid (1.42 g, 5.00 mmol, 1.0 equiv.) and methyl 4-aminobutanoate hydrochloride (768 mg, 5.00 mmol, 1.0 equiv.) to afford the product as a white solid (1.32 g, 69%).

^{1}H NMR (400 MHz, $CDCl_3$) δ 8.52 (s, 1H), 8.30 (d, *J* = 7.8 Hz, 1H), 8.22 (t, *J* = 7.3 Hz, 1H), 8.00 (d, *J* = 7.7 Hz, 1H), 7.66 – 7.55 (m, 2H), 7.38 – 7.27 (m, 2H), 6.75 (s, 1H), 3.69 (s, 3H), 3.55 (q, *J* = 6.5 Hz, 2H), 2.48 (t, *J* = 6.9 Hz, 2H), 2.01 (p, *J* = 6.8 Hz, 2H).
^{13}C NMR (101 MHz, $CDCl_3$) δ 174.3, 173.1 (d, *J* = 4.5 Hz), 168.3, 166.8, 160.9 (d, *J* = 260.6 Hz), 135.4, 134.9 (d, *J* = 8.6 Hz), 131.1, 130.4 (d, *J* = 11.0 Hz), 129.5, 127.2, 125.7, 124.9 (d, *J* = 3.8 Hz), 117.3 (d, *J* = 20.9 Hz), 112.8 (d, *J* = 11.3 Hz), 52.0, 40.0, 31.9, 24.6. One aromatic carbon not visible.
^{19}F NMR (282 MHz, $CDCl_3$) δ -108.18.
HRMS (FD+) (m/z): $[M+H]^{+}$ calcd. for $C_{20}H_{19}FN_3O_4^{+}$, 384.1354; found: 384.1360

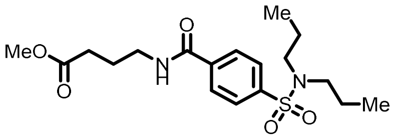

***Methyl 4-(4-(*N,N*-dipropylsulfamoyl)benzamido)butanoate* (1i).** Prepared according to **GP2** from 4-(*N,N*-dipropylsulfamoyl)benzoic acid (1.43 g, 5.00 mmol, 1.0 equiv.) and methyl 4-aminobutanoate hydrochloride (768 mg, 5.00 mmol, 1.0 equiv.) to afford the product as a white solid (1.54 g, 80%).

^{1}H NMR (400 MHz, $CDCl_3$) δ 7.89 (d, *J* = 8.6 Hz, 2H), 7.83 (d, *J* = 8.5 Hz, 2H), 6.88 (t, *J* = 5.8 Hz, 1H), 3.68 (s, 3H), 3.52 (q, *J* = 6.3 Hz, 2H), 3.11 – 3.03 (m, 4H), 2.47 (t, *J* = 6.8 Hz, 2H), 1.98 (p, *J* = 6.7 Hz, 2H), 1.53 (h, *J* = 7.5 Hz, 4H), 0.86 (t, *J* = 7.4 Hz, 6H).
^{13}C NMR (101 MHz, $CDCl_3$) δ 174.6, 166.3, 142.9, 138.1, 127.8, 127.4, 52.1, 50.1, 40.2, 32.0, 24.2, 22.1, 11.3.
HRMS (FD+) (m/z): $[M]^{+\cdot}$ calcd. for $C_{18}H_{28}N_2O_5S^{+\cdot}$, 384.1713; found: 384.1719.

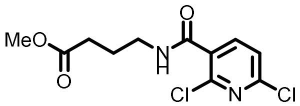

***Methyl 4-(2,6-dichloronicotinamido)butanoate* (1j).** Prepared according to **GP2** from 2,6-dichloronicotinic acid (960 mg, 5.00 mmol, 1.0 equiv.) and methyl 4-aminobutanoate hydrochloride (768 mg, 5.00 mmol, 1.0 equiv.) to afford the product as a white solid (1.19 g, 82%).

^{1}H NMR (300 MHz, $CDCl_3$) δ 8.05 (d, *J* = 8.0 Hz, 1H), 7.36 (d, *J* = 8.0 Hz, 1H), 6.76 (s, 1H), 3.68 (s, 3H), 3.53 (q, *J* = 6.7 Hz, 2H), 2.46 (t, *J* = 7.1 Hz, 2H), 1.97 (p, *J* = 7.0 Hz, 2H).
^{13}C NMR (101 MHz, $CDCl_3$) δ 173.8, 164.0, 151.8, 146.4, 142.2, 130.0, 123.6, 52.0, 40.0, 31.6, 24.4.
HRMS (FD+) (m/z): $[M]^{+\cdot}$ calcd. for $C_{11}H_{12}Cl_2N_2O_3^{+\cdot}$, 290.0219; found: 290.0225.

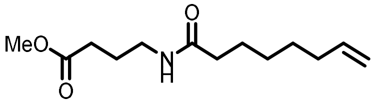

***Methyl 4-(oct-7-enamido)butanoate* (1k).** Prepared according to **GP2** from oct-7-enoic acid (711 mg, 5.00 mmol, 1.0 equiv.) and methyl 4-aminobutanoate hydrochloride (768 mg, 5.00 mmol, 1.0 equiv.) to afford the product as a light yellow oil (994 mg, 82%).

^{1}H NMR (300 MHz, $CDCl_3$) δ 5.79 (ddt, J = 16.9, 10.1, 6.7 Hz, 1H), 5.66 (s, 1H), 5.05 – 4.89 (m, 2H), 3.68 (s, 3H), 3.29 (q, J = 6.9 Hz, 2H), 2.37 (t, J = 7.1 Hz, 2H), 2.15 (t, J = 7.4 Hz, 2H), 2.09 – 1.99 (m, 2H), 1.84 (p, J = 7.0 Hz, 2H), 1.68 – 1.56 (m, 2H), 1.46 – 1.25 (m, 4H).
^{13}C NMR (101 MHz, $CDCl_3$) δ 174.1, 173.3, 139.0, 114.6, 51.9, 39.1, 36.9, 33.7, 31.7, 28.9, 28.7, 25.7, 24.8.
HRMS (FD+) (m/z): $[M+H]^+$ calcd. for $C_{13}H_{24}NO_3^+$, 242.1751; found: 242.1756.

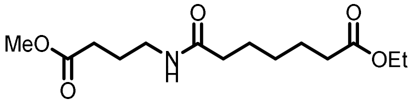

***Ethyl 7-((4-methoxy-4-oxobutyl)amino)-7-oxoheptanoate* (1l).** Prepared according to **GP2** from 7-ethoxy-7-oxoheptanoic acid (888 µL, 941 mg, 5.00 mmol, 1.0 equiv.) and methyl 4-aminobutanoate hydrochloride (768 mg, 5.00 mmol, 1.0 equiv.) to afford the product as a white solid (1.14 g, 79%).

^{1}H NMR (400 MHz, $CDCl_3$) δ 5.74 (s, 1H), 4.11 (q, J = 7.2 Hz, 2H), 3.68 (s, 3H), 3.29 (q, J = 6.5 Hz, 2H), 2.36 (t, J = 7.1 Hz, 2H), 2.29 (t, J = 7.4 Hz, 2H), 2.16 (t, J = 7.6 Hz, 2H), 1.83 (p, J = 7.0 Hz, 2H), 1.71 – 1.58 (m, 4H), 1.41 – 1.29 (m, 2H), 1.25 (t, J = 7.1 Hz, 3H).
^{13}C NMR (101 MHz, $CDCl_3$) δ 174.1, 173.8, 173.1, 60.4, 51.9, 39.1, 36.6, 34.2, 31.7, 28.8, 25.4, 24.8, 24.7, 14.4.
HRMS (FD+) (m/z): $[M]^{+\cdot}$ calcd. for $C_{14}H_{25}NO_5^{+\cdot}$, 287.1727; found: 287.1733.

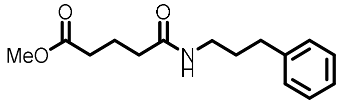

***Methyl 5-oxo-5-((3-phenylpropyl)amino)pentanoate* (1m).** Prepared according to **GP2** from 5-methoxy-5-oxopentanoic acid (731 mg, 5.00 mmol, 1.0 equiv.) and 3-phenylpropan-1-amine (711 µL, 676 mg, 5.00 mmol, 1.0 equiv.) to afford the product as a white solid (1.03 g, 78%).

^{1}H NMR (400 MHz, $CDCl_3$) δ 7.30 – 7.23 (m, 2H), 7.21 – 7.13 (m, 3H), 5.63 (s, 1H), 3.66 (s, 3H), 3.27 (q, J = 6.6 Hz, 2H), 2.64 (t, J = 7.7 Hz, 2H), 2.36 (t, J = 7.0 Hz, 2H), 2.18 (t, J = 7.3 Hz, 2H), 1.93 (p, J = 7.2 Hz, 2H), 1.83 (p, J = 7.4 Hz, 2H).
^{13}C NMR (101 MHz, $CDCl_3$) δ 173.8, 172.2, 141.5, 128.6, 128.5, 126.1, 51.7, 39.3, 35.6, 33.4, 33.2, 31.3, 21.0.
HRMS (FD+) (m/z): $[M]^{+\cdot}$ calcd. for $C_{15}H_{21}NO_3^{+\cdot}$, 263.1516; found: 263.1526.

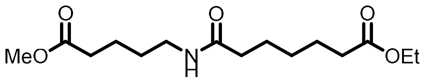

Ethyl 7-((5-methoxy-5-oxopentyl)amino)-7-oxoheptanoate **(1n).** Prepared according to **GP2** from 7-ethoxy-7-oxoheptanoic acid (731 mg, 5.00 mmol, 1.0 equiv.) and methyl 5-aminopentanoate hydrochloride (838 mg, 5.00 mmol, 1.0 equiv.) to afford the product as a white solid (0.99 g, 66%).

^{1}H NMR (400 MHz, $CDCl_3$) 5.52 (s, 1H), 4.12 (q, *J* = 7.1 Hz, 2H), 3.67 (s, 3H), 3.23 (q, *J* = 7.0 Hz, 2H), 2.38 (t, *J* = 7.1 Hz, 2H), 2.28 (t, *J* = 7.5 Hz, 2H), 2.22 (t, *J* = 7.4 Hz, 2H), 1.95 (p, *J* = 7.2 Hz, 2H), 1.67 – 1.57 (m, 2H), 1.53 – 1.44 (m, 2H), 1.38 – 1.29 (m, 4H), 1.25 (t, *J* = 7.1 Hz, 3H).
^{13}C NMR (101 MHz, $CDCl_3$) δ 173.9, 173.9, 172.1, 60.4, 51.7, 39.5, 35.7, 34.4, 33.2, 29.6, 28.9, 26.7, 24.9, 21.1, 14.4.
HRMS (FD+) (m/z): $[M]^{+\cdot}$ calcd. for $C_{15}H_{27}NO_5^{+\cdot}$, 301.1884; found: 301.1889.

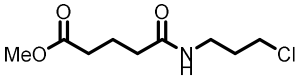

Methyl 5-((3-chloropropyl)amino)-5-oxopentanoate **(1o).** Prepared according to **GP2** from 5-methoxy-5-oxopentanoic acid (731 mg, 5.00 mmol, 1.0 equiv.) and 3-chloropropan-1-amine hydrochloride (650 mg, 5.00 mmol, 1.0 equiv.) to afford the product as a colorless oil (0.73 g, 55%).

^{1}H NMR (300 MHz, $CDCl_3$) δ 5.73 (s, 1H), 3.67 (s, 3H), 3.58 (t, *J* = 6.3 Hz, 2H), 3.41 (q, *J* = 6.5 Hz, 2H), 2.38 (t, *J* = 7.1 Hz, 2H), 2.24 (t, *J* = 7.3 Hz, 2H), 2.04 – 1.93 (m, 4H).
^{13}C NMR (101 MHz, $CDCl_3$) δ 173.8, 172.6, 51.8, 42.7, 37.1, 35.5, 33.2, 32.2, 21.0.
HRMS (EI+) (m/z): $[M]^{+\cdot}$ calcd. for $C_9H_{16}ClNO_3^{+\cdot}$, 221.0813; found: 221.0819.

11.2. Characterization of compounds 3-25 (*lactams*)

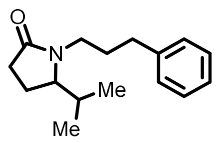

***5-Isopropyl-1-(3-phenylpropyl)pyrrolidin-2-one* (4).** Prepared according to **GP4** from **1b** (55 mg, 0.25 mmol, 1.0 equiv.) and 2-iodopropane (75 µL, 127 mg, 0.75 mmol, 3.0 equiv.). Purified via flash column chromatography on silica gel (Pentane:Ethyl Acetate 90:10 to 50:50) to afford the product as a light yellow oil (38 mg, 62% yield).

^{1}H NMR (400 MHz, $CDCl_3$) δ 7.31 – 7.25 (m, 2H), 7.21 – 7.15 (m, 3H), 3.81 – 3.70 (m, 1H), 3.58 – 3.51 (m, 1H), 2.88 (ddd, J = 13.8, 8.7, 5.0 Hz, 1H), 2.62 (t, J = 7.9 Hz, 2H), 2.40 – 2.23 (m, 2H), 2.07 – 1.98 (m, 1H), 1.89 – 1.67 (m, 4H), 0.90 (d, J = 7.0 Hz, 3H), 0.74 (d, J = 6.8 Hz, 3H).
^{13}C NMR (101 MHz, $CDCl_3$) δ 175.4, 141.6, 128.5, 128.4, 126.1, 62.0, 40.1, 33.5, 30.8, 28.8, 28.2, 18.7, 17.8, 14.3.
HRMS (FD+) (m/z): $[M+H]^+$ calcd. for $C_{16}H_{24}NO^+$, 246.1852; found: 246.1852.

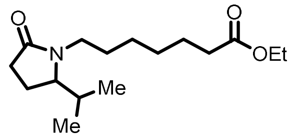

***Ethyl 7-(2-isopropyl-5-oxopyrrolidin-1-yl)heptanoate* (5).** Prepared according to **GP4** from **1c** (72 mg, 0.25 mmol, 1.0 equiv.) and 2-iodopropane (75 µL, 127 mg, 0.75 mmol, 3.0 equiv.). Purified via flash column chromatography on silica gel (Pentane:Ethyl Acetate 90:10 to 25:75) to afford the product as a light yellow oil (45 mg, 64% yield).

^{1}H NMR (400 MHz, $CDCl_3$) δ 4.10 (q, J = 7.1 Hz, 2H), 3.71 – 3.61 (m, 1H), 3.59 – 3.51 (m, 1H), 2.83 – 2.73 (m, 1H), 2.39 – 2.23 (m, 4H), 2.11 – 2.00 (m, 1H), 1.94 – 1.85 (m, 1H), 1.72 (dddd, J = 13.6, 9.1, 6.9, 4.9 Hz, 1H), 1.63 – 1.41 (m, 4H), 1.32 – 1.22 (m, 3H), 1.23 (t, J = 7.1 Hz, 4H), 0.91 (d, J = 6.9 Hz, 3H), 0.74 (d, J = 6.8 Hz, 3H).
^{13}C NMR (101 MHz, $CDCl_3$) δ 175.3, 173.9, 62.0, 60.3, 40.2, 34.3, 30.8, 28.9, 28.2, 26.9, 26.8, 25.0, 18.7, 17.8, 14.4, 14.3.
HRMS (FD+) (m/z): $[M]^{+\cdot}$ calcd. for $C_{16}H_{29}NO_3^{+\cdot}$, 283.2142; found: 283.2147.

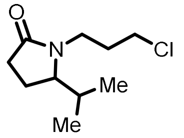

***1-(3-Chloropropyl)-5-isopropylpyrrolidin-2-one* (6).** Prepared according to **GP4** from **1d** (52 mg, 0.25 mmol, 1.0 equiv.) and 2-iodopropane (75 µL, 127 mg, 0.75 mmol, 3.0 equiv.). Purified via flash column chromatography on silica gel (Pentane:Ethyl Acetate 90:10 to 25:75) to afford the product as a light yellow oil (28 mg, 54% yield).

^{1}H NMR (300 MHz, $CDCl_3$) δ 3.78 – 3.65 (m, 1H), 3.64 – 3.49 (m, 3H), 3.12 – 2.99 (m, 1H), 2.43 – 2.24 (m, 2H), 2.18 – 2.02 (m, 2H), 2.02 – 1.85 (m, 2H), 1.85 – 1.67 (m, 1H), 0.94 (d, J = 6.9 Hz, 3H), 0.76 (d, J = 6.8 Hz, 3H).
^{13}C NMR (101 MHz, $CDCl_3$) δ 175.8, 62.7, 42.7, 38.3, 30.6, 30.2, 28.5, 18.7, 18.0, 14.4.
HRMS (FD+) (m/z): $[M]^{+\cdot}$ calcd. for $C_{10}H_{18}ClNO^{+\cdot}$, 203.1071; found: 203.1077.

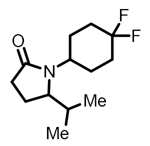

***1-(4,4-Difluorocyclohexyl)-5-isopropylpyrrolidin-2-one* (7).** Prepared according to **GP4** from **1e** (62 mg, 0.25 mmol, 1.0 equiv.) and 2-iodopropane (75 µL, 127 mg, 0.75 mmol, 3.0 equiv.). Purified via flash column chromatography on silica gel (Pentane:Ethyl Acetate 75:25 to 25:75) to afford the product as a light yellow oil (42 mg, 69% yield).

^{1}H NMR (400 MHz, $CDCl_3$) δ 3.89 – 3.77 (m, 1H), 3.61 (dt, *J* = 9.1, 2.9 Hz, 1H), 2.42 – 2.00 (m, 6H), 1.97 – 1.67 (m, 7H), 0.92 (d, *J* = 6.9 Hz, 3H), 0.79 (d, *J* = 6.7 Hz, 3H).
^{13}C NMR (101 MHz, $CDCl_3$) δ 175.5, 122.5 (dd, *J* = 243.3, 239.7 Hz), 62.3 (d, *J* = 2.4 Hz), 50.8, 33.4 (dd, *J* = 24.3, 8.6 Hz), 33.1 (dd, *J* = 23.7, 8.0 Hz), 31.2, 27.1 (d, *J* = 10.0 Hz), 25.3 (d, *J* = 10.3 Hz), 19.2, 19.2, 18.3, 14.3.
^{19}F NMR (282 MHz, $CDCl_3$) δ -93.00 (d, *J* = 237.5 Hz), -102.61 (d, *J* = 237.2 Hz).
HRMS (FD+) (m/z): $[M]^{+\cdot}$ calcd. for $C_{13}H_{21}F_2NO^{+\cdot}$, 245.1586; found: 245.1586.

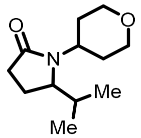

***5-Isopropyl-1-(tetrahydro-2*H*-pyran-4-yl)pyrrolidin-2-one* (8).** Prepared according to **GP4** from **1f** (54 mg, 0.25 mmol, 1.0 equiv.) and 2-iodopropane (75 µL, 127 mg, 0.75 mmol, 3.0 equiv.). Purified via flash column chromatography on silica gel (Pentane:Ethyl Acetate 50:50 to Ethyl Acetate to Ethyl Acetate:Methanol 95:5) to afford the product as a yellow oil (23 mg, 44% yield).

^{1}H NMR (400 MHz, $CDCl_3$) δ 4.08 – 3.90 (m, 3H), 3.64 (dt, *J* = 9.1, 2.9 Hz, 1H), 3.49 – 3.39 (m, 2H), 2.44 – 2.23 (m, 2H), 2.19 – 2.04 (m, 2H), 1.98 – 1.73 (m, 4H), 1.62 – 1.52 (m, 1H), 0.93 (d, *J* = 6.9 Hz, 3H), 0.81 (d, *J* = 6.7 Hz, 3H).
^{13}C NMR (101 MHz, $CDCl_3$) δ 175.5, 67.8, 67.7, 62.5, 50.5, 31.7, 31.4, 31.1, 29.8, 19.2, 18.3, 14.3.
HRMS (ESI+) (m/z): $[M]^{+\cdot}$ calcd. for $C_{12}H_{21}NO_2^{+\cdot}$, 211.1567; found: 211.1562.

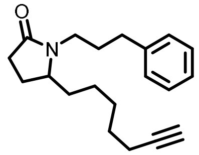

***5-(Hept-6-yn-1-yl)-1-(3-phenylpropyl)pyrrolidin-2-one* (9).** Prepared according to **GP4** from **1b** (62 mg, 0.25 mmol, 1.0 equiv.) and 7-iodohept-1-yne (167 mg, 0.75 mmol, 3.0 equiv.). Purified via flash column chromatography on silica gel (Pentane:Ethyl Acetate 90:10 to 25:75) to afford the product as a yellow oil (15 mg, 20% yield).

^{1}H NMR (400 MHz, $CDCl_3$) δ 7.31 – 7.26 (m, 2H), 7.22 – 7.16 (m, 3H), 3.72 – 3.60 (m, 1H), 3.58 – 3.47 (m, 1H), 2.99 – 2.89 (m, 1H), 2.62 (t, *J* = 7.8 Hz, 2H), 2.43 – 2.23 (m, 2H), 2.19 (td, *J* = 6.9, 2.7 Hz, 2H), 2.12 – 2.00 (m, 1H), 1.95 (t, *J* = 2.7 Hz, 1H), 1.90 – 1.75 (m, 2H), 1.70 – 1.60 (m, 2H), 1.52 (p, *J* = 6.7 Hz, 2H), 1.47 – 1.36 (m, 2H), 1.33 – 1.23 (m, 3H).
^{13}C NMR (101 MHz, $CDCl_3$) δ 175.1, 141.6, 128.5, 128.4, 126.1, 84.5, 68.5, 57.6, 40.1, 33.5, 33.2, 30.5, 29.1, 28.8, 28.4, 24.3, 24.2, 18.5.
HRMS (FD+) (m/z): $[M]^{+\cdot}$ calcd. for $C_{20}H_{27}NO^{+\cdot}$, 297.2087; found: 297.2093.

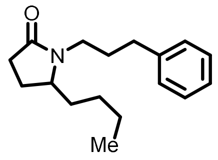

***5-Butyl-1-(3-phenylpropyl)pyrrolidin-2-one* (10).** Prepared according to **GP4** from **1b** (62 mg, 0.25 mmol, 1.0 equiv.) and 1-iodobutane (85 µL, 138 mg, 0.75 mmol, 3.0 equiv.). Purified via flash column chromatography on silica gel (Pentane:Ethyl Acetate 90:10 to 50:50) to afford the product as a light yellow oil (26 mg, 40% yield).

^{1}H NMR (400 MHz, $CDCl_3$) δ 7.32 – 7.23 (m, 2H), 7.22 – 7.14 (m, 3H), 3.72 – 3.62 (m, 1H), 3.60 – 3.48 (m, 1H), 3.00 – 2.89 (m, 1H), 2.62 (t, J = 7.8 Hz, 2H), 2.43 – 2.23 (m, 2H), 2.12 – 2.00 (m, 1H), 1.92 – 1.74 (m, 2H), 1.67 – 1.58 (m, 2H), 1.38 – 1.18 (m, 5H), 0.90 (t, J = 7.1 Hz, 3H).
^{13}C NMR (101 MHz, $CDCl_3$) δ 175.1, 141.7, 128.5, 128.4, 126.1, 57.6, 40.1, 33.5, 33.0, 30.5, 29.1, 26.9, 24.2, 22.9, 14.2.
HRMS (FD+) (m/z): $[M]^{+\cdot}$ calcd. for $C_{17}H_{25}NO^{+\cdot}$, 259.1931; found: 259.1936.

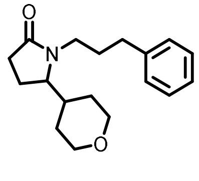

***1-(3-Phenylpropyl)-5-(tetrahydro-2*H*-pyran-4-yl)pyrrolidin-2-one* (11).** Prepared according to **GP4** from **1b** (62 mg, 0.25 mmol, 1.0 equiv.) and 4-iodotetrahydro-2*H*-pyran (90 µL, 159 mg, 0.75 mmol, 3.0 equiv.). Purified via flash column chromatography on silica gel (Pentane:Ethyl Acetate 50:50 to Ethyl Acetate to Ethyl Acetate:Methanol 95:5) to afford the product as a yellow oil (35 mg, 49% yield).

^{1}H NMR (400 MHz, $CDCl_3$) δ 7.32 – 7.23 (m, 2H), 7.22 – 7.14 (m, 3H), 4.05 – 3.95 (m, 2H), 3.82 – 3.73 (m, 1H), 3.56 – 3.50 (m, 1H), 3.35 (td, J = 11.7, 2.5 Hz, 1H), 3.27 (td, J = 11.4, 2.9 Hz, 1H), 2.90 – 2.81 (m, 1H), 2.69 – 2.56 (m, 2H), 2.39 – 2.23 (m, 2H), 1.96 – 1.76 (m, 5H), 1.45 (qd, J = 12.2, 4.5 Hz, 1H), 1.37 – 1.24 (m, 3H).
^{13}C NMR (101 MHz, $CDCl_3$) δ 175.3, 141.5, 128.6, 128.4, 126.2, 68.0, 67.9, 60.9, 40.2, 36.7, 33.4, 30.6, 29.0, 28.7, 25.5, 19.3.
HRMS (FD+) (m/z): $[M]^{+\cdot}$ calcd. for $C_{18}H_{25}NO_2^{+\cdot}$, 287.1880; found: 287.1874.

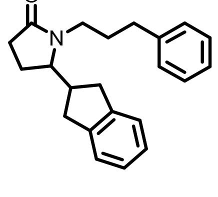

***5-(2,3-Dihydro-1*H*-inden-2-yl)-1-(3-phenylpropyl)pyrrolidin-2-one* (12).** Prepared according to **GP4** from **1b** (62 mg, 0.25 mmol, 1.0 equiv.) and 2-iodo-2,3-dihydro-1*H*-indene (183 mg, 0.75 mmol, 3.0 equiv.). Purified via flash column chromatography on silica gel (Pentane:Ethyl Acetate 90:10 to 60:40) to afford the product as a light yellow oil (47 mg, 60% yield).

^{1}H NMR (300 MHz, $CDCl_3$) δ 7.34 – 7.25 (m, 2H), 7.23 – 7.12 (m, 7H), 3.87 – 3.74 (m, 2H), 3.12 – 2.95 (m, 2H), 2.94 – 2.79 (m, 2H), 2.77 – 2.59 (m, 4H), 2.43 – 2.22 (m, 2H), 2.02 – 1.79 (m, 3H), 1.71 – 1.53 (m, 1H).

^{13}C NMR (101 MHz, CDCl$_3$) δ 175.5, 142.4, 142.3, 141.5, 128.5, 128.4, 126.7, 126.7, 126.1, 124.6, 124.5, 60.5, 40.6, 40.3, 35.8, 33.5, 33.4, 30.5, 29.0, 20.2.
HRMS (FD+) (m/z): [M]$^{+\cdot}$ calcd. for $C_{22}H_{25}NO^{+\cdot}$, 319.1931; found: 319.1933.

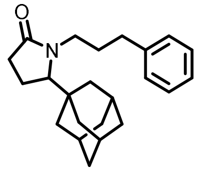

5-((3r,5r,7r)-Adamantan-1-yl)-1-(3-phenylpropyl)pyrrolidin-2-one **(13).** Prepared according to **GP4** from **1b** (62 mg, 0.25 mmol, 1.0 equiv.) and 1-iodoadamantane (197 mg, 0.75 mmol, 3.0 equiv.). Purified via flash column chromatography on silica gel (Pentane:Ethyl Acetate 90:10 to 50:50) to afford the product as a yellow solid (63 mg, 75% yield).

^{1}**H NMR** (300 MHz, CDCl$_3$) δ 7.32 – 7.25 (m, 2H), 7.22 – 7.13 (m, 3H), 3.90 (ddd, *J* = 13.8, 9.1, 6.6 Hz, 1H), 3.17 – 2.98 (m, 2H), 2.62 – 2.53 (m, 2H), 2.44 – 2.29 (m, 1H), 2.17 (ddd, *J* = 17.0, 10.0, 2.6 Hz, 1H), 2.04 – 1.91 (m, 5H), 1.91 – 1.78 (m, 2H), 1.75 – 1.64 (m, 3H), 1.64 – 1.55 (m, 3H), 1.55 – 1.42 (m, 6H).
^{13}**C NMR** (101 MHz, CDCl$_3$) δ 177.1, 141.6, 128.5, 128.4, 126.0, 67.4, 44.6, 39.0, 38.9, 37.0, 33.4, 31.3, 28.7, 28.2, 20.9.
HRMS (FD+) (m/z): [M]$^{+\cdot}$ calcd. for $C_{23}H_{31}NO^{+\cdot}$, 337.2400; found: 337.2406.

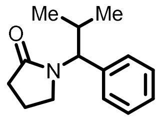

1-(2-Methyl-1-phenylpropyl)pyrrolidin-2-one **(3).** Prepared according to **GP4** from **1a** (55 mg, 0.25 mmol, 1.0 equiv.) and 2-iodopropane (75 µL, 127 mg, 0.75 mmol, 3.0 equiv.). Purified via flash column chromatography on silica gel (Pentane:Ethyl Acetate 90:10 to 50:50) to afford the product as a light yellow oil (38 mg, 70% yield).

^{1}**H NMR** (400 MHz, CDCl$_3$) δ 7.30 – 7.24 (m, 3H), 7.24 – 7.18 (m, 2H), 4.79 (d, *J* = 11.4 Hz, 1H), 3.35 – 3.23 (m, 1H), 3.08 – 2.96 (m, 1H), 2.41 – 2.17 (m, 3H), 1.99 – 1.76 (m, 2H), 0.93 (d, *J* = 6.5 Hz, 3H), 0.78 (d, *J* = 6.5 Hz, 3H).
^{13}**C NMR** (101 MHz, CDCl$_3$) δ 174.8, 138.7, 128.7, 128.7, 127.7, 61.6, 42.8, 31.5, 27.6, 20.3, 19.8, 18.1.
HRMS (ESI+) (m/z): [M+H]$^+$ calcd. for $C_{14}H_{20}NO^+$, 218.1539; found: 218.1545.

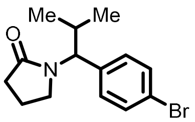

1-(1-(4-Bromophenyl)-2-methylpropyl)pyrrolidin-2-one **(14).** Prepared according to **GP4** in batch from **1g** (75 mg, 0.25 mmol, 1.0 equiv.) and 2-iodopropane (75 µL, 127 mg, 0.75 mmol, 3.0 equiv.). Purified via flash column chromatography on silica gel (Pentane:Ethyl Acetate 90:10 to 50:50) to afford the product as a light yellow oil (51 mg, 68% yield).

^{1}H NMR (400 MHz, $CDCl_3$) δ 7.45 (d, *J* = 8.4 Hz, 2H), 7.19 (d, *J* = 8.4 Hz, 2H), 4.79 (d, *J* = 11.5 Hz, 1H), 3.35 – 3.27 (m, 1H), 3.07 – 2.99 (m, 1H), 2.46 – 2.21 (m, 3H), 2.02 – 1.79 (m, 2H), 0.97 (d, *J* = 6.5 Hz, 3H), 0.82 (d, *J* = 6.5 Hz, 3H).
^{13}C NMR (101 MHz, $CDCl_3$) δ 174.9, 137.7, 131.8, 130.3, 121.6, 61.0, 42.7, 31.3, 27.6, 20.3, 19.7, 18.1.
HRMS (FD+) (m/z): $[M]^{+\cdot}$ calcd. for $C_{14}H_{18}BrNO^{+\cdot}$, 295.0566; found: 295.0572.

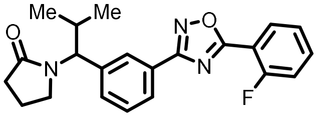

1-(1-(3-(5-(2-Fluorophenyl)-1,2,4-oxadiazol-3-yl)phenyl)-2-methylpropyl)pyrrolidin-2-one **(15).** Prepared according to **GP4** in batch from **1h** (96 mg, 0.25 mmol, 1.0 equiv.) and 2-iodopropane (75 µL, 127 mg, 0.75 mmol, 3.0 equiv.). Purified via flash column chromatography on silica gel (Pentane:Ethyl Acetate 90:10 to 25:75) to afford the product as a light yellow oil (45 mg, 47% yield).

^{1}H NMR (300 MHz, $CDCl_3$) δ 8.23 (ddd, *J* = 7.8, 7.0, 1.8 Hz, 1H), 8.14 – 8.06 (m, 2H), 7.65 – 7.55 (m, 1H), 7.55 – 7.43 (m, 2H), 7.38 – 7.27 (m, 2H), 4.95 (d, *J* = 11.5 Hz, 1H), 3.41 (ddd, *J* = 9.6, 8.4, 4.9 Hz, 1H), 3.13 (ddd, *J* = 9.5, 8.2, 6.5 Hz, 1H), 2.58 – 2.22 (m, 3H), 2.09 – 1.77 (m, 2H), 1.02 (d, *J* = 6.5 Hz, 3H), 0.87 (d, *J* = 6.5 Hz, 3H).
^{13}C NMR (101 MHz, $CDCl_3$) δ 174.9, 173.0 (d, *J* = 4.3 Hz), 168.7, 160.9 (d, *J* = 260.6 Hz), 139.6 (2C), 134.8 (d, *J* = 8.5 Hz), 132.1, 131.1, 129.5, 127.2, 127.0 (d, *J* = 7.8 Hz), 124.8 (d, *J* = 3.8 Hz), 117.3 (d, *J* = 21.1 Hz), 112.9 (d, *J* = 11.5 Hz), 61.5, 42.9, 31.4, 27.7, 20.4, 19.7, 18.2.
^{19}F NMR (282 MHz, $CDCl_3$) δ -108.30.
HRMS (FD+) (m/z): $[M]^{+\cdot}$ calcd. for $C_{22}H_{22}FN_3O_2^{+\cdot}$, 379.1691; found: 379.1696.

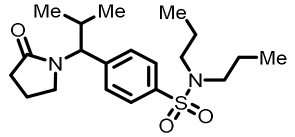

4-(2-Methyl-1-(2-oxopyrrolidin-1-yl)propyl)-N,N-dipropylbenzenesulfonamide **(16).** Prepared according to **GP4** in batch from **1i** (96 mg, 0.25 mmol, 1.0 equiv.) and 2-iodopropane (75 µL, 127 mg, 0.75 mmol, 3.0 equiv.). Purified via flash column chromatography on silica gel (Pentane:Ethyl Acetate 75:25 to 50:50) to afford the product as a pale yellow oil (48 mg, 50% yield).

^{1}H NMR (300 MHz, $CDCl_3$) δ 7.76 (d, *J* = 8.4 Hz, 2H), 7.44 (d, *J* = 8.4 Hz, 2H), 4.89 (d, *J* = 11.5 Hz, 1H), 3.35 (ddd, *J* = 9.5, 8.4, 4.8 Hz, 1H), 3.14 – 2.96 (m, 5H), 2.49 – 2.22 (m, 3H), 2.08 – 1.82 (m, 2H), 1.65 – 1.53 (m, 4H), 1.00 (d, *J* = 6.5 Hz, 3H), 0.89 – 0.79 (m, 9H).
^{13}C NMR (101 MHz, $CDCl_3$) δ 175.0, 143.3, 139.6, 129.1, 127.5, 61.2, 50.2, 42.8, 31.3, 27.6, 22.3, 20.2, 19.7, 18.2, 11.3.
HRMS (FD+) (m/z): $[M+H]^{+}$ calcd. for $C_{20}H_{33}N_2O_3S^{+\cdot}$, 381.2206; found: 381.2212.

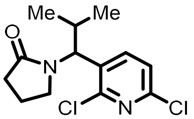

1-(1-(2,6-Dichloropyridin-3-yl)-2-methylpropyl)pyrrolidin-2-one **(17).** Prepared according to **GP5** from **1j** (73 mg, 0.25 mmol, 1.0 equiv.) and 2-iodopropane (75 µL, 127 mg, 0.75 mmol, 3.0 equiv.). Purified via flash column chromatography on silica gel (Pentane:Ethyl Acetate 90:10 to 25:75) to afford the product as a yellow oil (26 mg, 36% yield).

¹H NMR (300 MHz, $CDCl_3$) δ 7.88 (d, *J* = 8.1 Hz, 1H), 7.28 (d, *J* = 8.2 Hz, 1H), 4.91 (d, *J* = 11.3 Hz, 1H), 3.46 – 3.31 (m, 1H), 3.17 – 3.01 (m, 1H), 2.70 – 2.52 (m, 1H), 2.43 – 2.28 (m, 2H), 2.06 – 1.87 (m, 2H), 1.05 (d, *J* = 6.5 Hz, 3H), 0.81 (d, *J* = 6.6 Hz, 3H).
¹³C NMR (101 MHz, $CDCl_3$) δ 175.0, 151.2, 149.3, 140.7, 132.1, 123.0, 59.4, 45.8, 31.4, 28.3, 19.9, 19.8, 18.5.
HRMS (FD+) (m/z): $[M]^{+\cdot}$ calcd. for $C_{13}H_{16}Cl_2N_2O^{+\cdot}$, 286.0634; found: 286.0640.

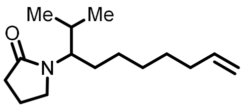

1-(2-Methyldec-9-en-3-yl)pyrrolidin-2-one **(18).** Prepared according to **GP4** in batch from **1k** (60 mg, 0.25 mmol, 1.0 equiv.) and 2-iodopropane (75 µL, 127 mg, 0.75 mmol, 3.0 equiv.). Purified via flash column chromatography on silica gel (Pentane:Ethyl Acetate 90:10 to 60:40) to afford the product as a light yellow oil (23 mg, 38% yield).

¹H NMR (400 MHz, $CDCl_3$) δ 5.79 (ddt, *J* = 16.9, 10.2, 6.7 Hz, 1H), 5.04 – 4.83 (m, 2H), 3.75 – 3.63 (m, 1H), 3.21 (t, *J* = 7.0 Hz, 2H), 2.41 (t, *J* = 8.0 Hz, 2H), 2.07 – 1.88 (m, 4H), 1.68 – 1.55 (m, 2H), 1.40 – 1.12 (m, 7H), 0.95 (d, *J* = 6.6 Hz, 3H), 0.82 (d, *J* = 6.7 Hz, 3H).
¹³C NMR (101 MHz, $CDCl_3$) δ 175.6, 139.2, 114.4, 57.3, 42.6, 33.8, 31.6, 30.8, 29.5, 29.1, 29.0, 26.4, 20.2, 20.0, 18.6.
HRMS (FD+) (m/z): $[M]^{+\cdot}$ calcd. for $C_{15}H_{27}NO^{+\cdot}$, 237.2087; found: 237.2093.

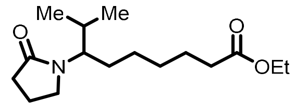

Ethyl 8-methyl-7-(2-oxopyrrolidin-1-yl)nonanoate **(19).** Prepared according to **GP4** from **1l** (72 mg, 0.25 mmol, 1.0 equiv.) and 2-iodopropane (75 µL, 127 mg, 0.75 mmol, 3.0 equiv.). Purified via flash column chromatography on silica gel (Pentane:Ethyl Acetate 90:10 to 25:75) to afford the product as a light yellow oil (39 mg, 55% yield).

¹H NMR (400 MHz, $CDCl_3$) δ 4.10 (q, *J* = 7.1 Hz, 2H), 3.72 – 3.61 (m, 1H), 3.21 (t, *J* = 6.9 Hz, 2H), 2.41 (t, *J* = 8.0 Hz, 2H), 2.26 (t, *J* = 7.5 Hz, 2H), 1.98 (p, *J* = 7.6 Hz, 2H), 1.70 – 1.50 (m, 4H), 1.39 – 1.09 (m, 5H), 1.23 (t, *J* = 7.1 Hz, 3H), 0.94 (d, *J* = 6.6 Hz, 3H), 0.81 (d, *J* = 6.6 Hz, 3H).
¹³C NMR (101 MHz, $CDCl_3$) δ 175.7, 173.9, 60.3, 57.3, 42.6, 34.4, 31.5, 30.8, 29.3, 29.1, 26.1, 25.0, 20.1, 20.0, 18.6, 14.4.
HRMS (FD+) (m/z): $[M]^{+\cdot}$ calcd. for $C_{16}H_{29}NO_3^{+\cdot}$, 283.2142; found: 283.2147.

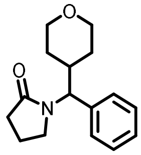

1-(phenyl(tetrahydro-2H-pyran-4-yl)methyl)pyrrolidin-2-one **(20).** Prepared according to **GP4** from **1a** (55 mg, 0.25 mmol, 1.0 equiv.) and 4-iodotetrahydro-2H-pyran (90 µL, 159 mg, 0.75 mmol, 3.0 equiv.). Purified via flash column chromatography on silica gel (Pentane:Ethyl Acetate 50:50 to 0:100) to afford the product as a pale yellow oil (37 mg, 57% yield).

¹H NMR (400 MHz, $CDCl_3$) δ 7.37 – 7.27 (m, 5H), 4.95 (d, *J* = 11.4 Hz, 1H), 4.04 (dt, *J* = 11.5, 3.4 Hz, 1H), 3.92 – 3.85 (m, 1H), 3.46 – 3.27 (m, 3H), 3.15 – 3.06 (m, 1H), 2.46 – 2.25 (m, 3H), 1.99 – 1.85 (m, 2H), 1.55 – 1.50 (m, 1H), 1.38 – 1.20 (m, 3H).
¹³C NMR ¹³C NMR (101 MHz, $CDCl_3$) δ 175.0, 137.3, 128.9, 128.8, 128.0, 67.8, 67.6, 60.2, 43.2, 34.6, 31.4, 30.7, 30.2, 18.1.

HRMS (FD+) (m/z): $[M]^{+\cdot}$ calcd. for $C_{16}H_{21}NO_2^{+\cdot}$, 259.1567; found: 259.1562.

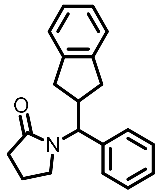

1-((2,3-dihydro-1H-inden-2-yl)(phenyl)methyl)pyrrolidin-2-one **(21).** Prepared according to **GP4** from **1a** (55mg, 0.25 mmol, 1.0 equiv.) and 2-iodo-2,3-dihydro-1*H*-indene (183 mg, 0.75 mmol, 3.0 equiv.). Purified via flash column chromatography on silica gel (Pentane:Ethyl Acetate 50:50 to 0:100) to afford the product as a yellow oil (40 mg, 55% yield).

^{1}H NMR (300 MHz, $CDCl_3$) δ 7.45 – 7.29 (m, 5H), 7.22 – 7.06 (m, 4H), 5.30 (d, *J* = 11.7 Hz, 1H), 3.49 – 3.38 (m, 1H), 3.36 – 3.26 (m, 1H), 3.18 – 2.84 (m, 4H), 2.65 – 2.51 (m, 1H), 2.50 – 2.27 (m, 2H), 2.10 – 1.79 (m, 2H).
^{13}C NMR (101 MHz, $CDCl_3$) δ 174.8, 142.7, 142.5, 138.8, 128.8, 128.6, 128.0, 126.6, 126.5, 124.6, 124.5, 59.5, 43.0, 39.7, 37.7, 37.0, 31.5, 18.2.
HRMS (FD+) (m/z): $[M]^{+\cdot}$ calcd. for $C_{20}H_{21}NO^{+\cdot}$, 291.1618; found: 291.1630.

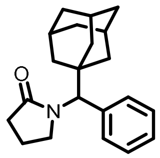

1-(((3r,5r,7r)-adamantan-1-yl)(phenyl)methyl)pyrrolidin-2-one **(22).** Prepared according to **GP4** from **1a** (55 mg, 0.25 mmol, 1.0 equiv.) and 1-iodoadamantane (197 mg, 0.75 mmol, 3.0 equiv.). Purified via flash column chromatography on silica gel (Pentane:Ethyl Acetate 90:10 to 50:50) to afford the product as a white solid (36 mg, 46% yield).

^{1}H NMR (400 MHz, CDCl3) δ 7.40 – 7.35 (m, 2H), 7.35 – 7.28 (m, 3H), 4.92 (s, 1H), 3.79 (td, *J* = 8.8, 3.8 Hz, 1H), 3.45 (q, *J* = 8.2 Hz, 1H), 2.47 – 2.35 (m, 1H), 2.32 – 2.15 (m, 1H), 2.07 – 1.84 (m, 5H), 1.78 – 1.71 (m, 3H), 1.70 – 1.64 (m, 3H), 1.60 (d, *J* = 12.5 Hz, 6H).
^{13}C NMR (101 MHz, $CDCl_3$) δ 175.8, 136.9, 130.8, 128.2, 127.5, 66.2, 47.8, 40.5, 38.7, 37.0, 31.0, 28.7, 18.7.
HRMS (FD+) (m/z): $[M]^{+\cdot}$ calcd. for $C_{21}H_{27}NO^{+\cdot}$, 309.2087; found: 309.2085.

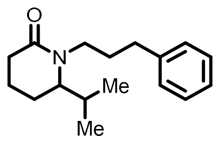

6-Isopropyl-1-(3-phenylpropyl)piperidin-2-one **(23).** Prepared according to **GP4** from **1m** (66 mg, 0.25 mmol, 1.0 equiv.) and 2-iodopropane (75 µL, 127 mg, 0.75 mmol, 3.0 equiv.). Purified via flash column chromatography on silica gel (Pentane:Ethyl Acetate 90:10 to 50:50) to afford the product as a colorless oil (31 mg, 47% yield).

^{1}H NMR (400 MHz, $CDCl_3$) δ 7.30 – 7.26 (m, 2H), 7.21 – 7.15 (m, 3H), 4.02 – 3.90 (m, 1H), 3.20 (q, *J* = 6.1 Hz, 1H), 2.97 – 2.86 (m, 1H), 2.69 – 2.55 (m, 2H), 2.42 (dt, *J* = 17.6, 5.7 Hz, 1H), 2.31 – 2.21 (m, 1H), 2.12 – 1.96 (m, 1H), 1.94 – 1.69 (m, 4H), 1.68 – 1.53 (m, 2H), 0.91 (d, *J* = 6.9 Hz, 3H), 0.81 (d, *J* = 6.8 Hz, 3H).
^{13}C NMR (101 MHz, $CDCl_3$) δ 171.5, 141.9, 128.5, 128.4, 126.0, 61.4, 45.0, 33.6, 32.4, 29.8, 29.0, 23.3, 19.6, 18.7, 16.5.

HRMS (FD+) (m/z): $[M]^{+\cdot}$ calcd. for $C_{17}H_{25}NO^{+\cdot}$, 259.1931; found: 259.1933.

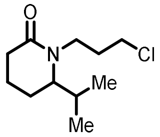

1-(3-Chloropropyl)-6-isopropylpiperidin-2-one **(24).** Prepared according to **GP4** from **1o** (59.8 mg, 0.25 mmol, 1.0 equiv.) and 2-iodopropane (75 µL, 127 mg, 0.75 mmol, 3.0 equiv.). Purified via flash column chromatography on silica gel (Pentane:Ethyl Acetate 50:50) to afford the product as a colorless oil (26 mg, 48% yield).

^{1}H NMR (400 MHz, $CDCl_3$) δ 3.88 (ddd, J = 13.9, 8.7, 5.5 Hz, 1H), 3.61 – 3.50 (m, 2H), 3.31 – 3.22 (m, 1H), 3.19 – 3.08 (m, 1H), 2.48 – 2.35 (m, 1H), 2.31 – 2.19 (m, 1H), 2.20 – 2.07 (m, 2H), 2.06 – 1.90 (m, 1H), 1.90 – 1.74 (m, 2H), 1.69 – 1.58 (m, 2H), 0.96 (d, J = 6.8 Hz, 3H), 0.85 (d, J = 6.6 Hz, 3H).
^{13}C NMR (101 MHz, $CDCl_3$) δ 171.7, 62.4, 43.6, 43.2, 32.4, 30.5, 30.1, 23.4, 19.7, 18.7, 16.6.
HRMS (FD+) (m/z): $[M]^{+\cdot}$ calcd. for $C_{11}H_{20}ClNO^{+\cdot}$, 217.1228; found: 217.123

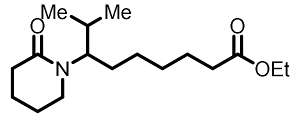

Ethyl 8-methyl-7-(2-oxopiperidin-1-yl)nonanoate **(25).** Prepared according to **GP4** from **1n** (75 mg, 0.25 mmol, 1.0 equiv.) and 2-iodopropane (75 µL, 127 mg, 0.75 mmol, 3.0 equiv.). Purified via flash column chromatography on silica gel (Pentane:Ethyl Acetate 75:25 to 25:75) to afford the product as a light yellow oil (49 mg, 65% yield).

^{1}H NMR (300 MHz, $CDCl_3$) δ 4.11 (q, J = 7.2 Hz, 2H), 3.91 (ddd, J = 13.5, 9.5, 6.3 Hz, 1H), 3.21 (dt, J = 7.2, 5.6 Hz, 1H), 2.82 (ddd, J = 14.0, 9.3, 5.3 Hz, 1H), 2.46 – 2.35 (m, 1H), 2.28 (t, J = 7.5 Hz, 2H), 2.16 – 2.04 (m, 1H), 1.90 – 1.72 (m, 2H), 1.70 – 1.41 (m, 7H), 1.39 – 1.22 (m, 4H), 1.25 (t, J = 7.2 Hz, 3H), 0.94 (d, J = 6.9 Hz, 3H), 0.82 (d, J = 6.8 Hz, 3H).
^{13}C NMR (101 MHz, $CDCl_3$) δ 173.9, 171.3, 61.3, 60.3, 45.0, 34.4, 32.4, 29.8, 29.0, 27.2, 26.9, 25.0, 23.3, 19.7, 18.7, 16.5, 14.3.
HRMS (FD+) (m/z): $[M+H]^+$ calcd. for $C_{17}H_{32}NO_3^+$, 298.2377; found: 298.2382.

12.References

(1) Masson, T. M.; Zondag, S. D. A.; Schuurmans, J. H. A.; Noël, T. Open-Source 3D Printed Reactors for Reproducible Batch and Continuous-Flow Photon-Induced Chemistry: Design and Characterization. *React. Chem. Eng.* **2024**, *9* (8), 2218–2225. https://doi.org/10.1039/D4RE00081A.

(2) Zhang, Z.; Górski, B.; Leonori, D. Merging Halogen-Atom Transfer (XAT) and Copper Catalysis for the Modular Suzuki–Miyaura-Type Cross-Coupling of Alkyl Iodides and Organoborons. *J. Am. Chem. Soc.* **2022**, *144* (4), 1986–1992. https://doi.org/10.1021/jacs.1c12649.

(3) Morgan, D. C.; McDougall, L.; Knuhtsen, A.; Jamieson, A. G. Development of Bifunctional, Raman Active Diyne-Girder Stapled α-Helical Peptides. *Chemistry – A European Journal* **2023**, *29* (41), e202300855. https://doi.org/10.1002/chem.202300855.

(4) Liu, C.; Zhang, Z.; Zhao, L.-L.; Bertrand, G.; Yan, X. Mesoionic Carbene-Catalyzed Formyl Alkylation of Aldehydes. *Angewandte Chemie International Edition* **2023**, *62* (24), e202303478. https://doi.org/10.1002/anie.202303478.

(5) Blackwell, J. H.; Kumar, R.; Gaunt, M. J. Visible-Light-Mediated Carbonyl Alkylative Amination to All-Alkyl α-Tertiary Amino Acid Derivatives. *J. Am. Chem. Soc.* **2021**, *143* (3), 1598–1609. https://doi.org/10.1021/jacs.0c12162.

(6) König, H. F.; Rummel, L.; Hausmann, H.; Becker, J.; Schümann, J. M.; Schreiner, P. R. Gauging the Steric Effects of Silyl Groups with a Molecular Balance. *J. Org. Chem.* **2022**, *87* (7), 4670–4679. https://doi.org/10.1021/acs.joc.1c03103.

(7) Kehner, R. A.; Zhang, G.; Bayeh-Romero, L. Mild Divergent Semireductive Transformations of Secondary and Tertiary Amides via Zirconocene Hydride Catalysis. *J. Am. Chem. Soc.* **2023**, *145* (9), 4921–4927. https://doi.org/10.1021/jacs.2c11786.

13.NMR spectra of starting materials

^{1}H NMR (400 MHz, $CDCl_3$) of **1a**

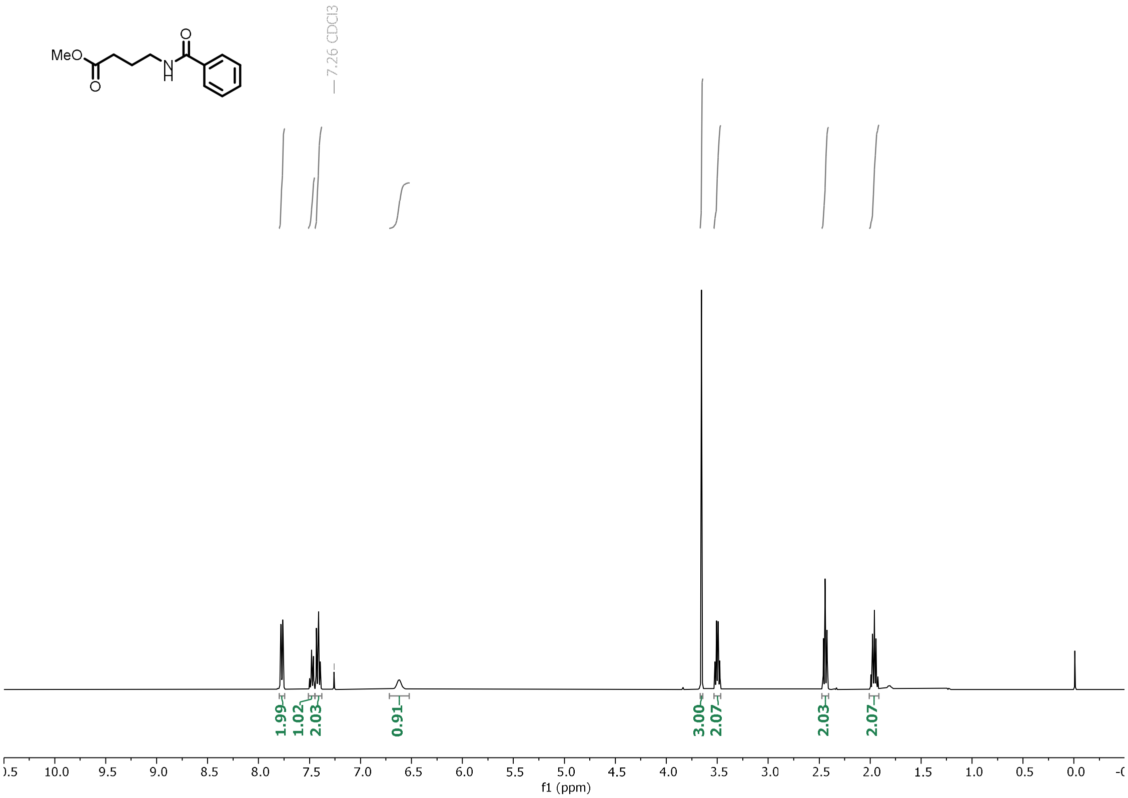

^{13}C NMR (101 MHz, $CDCl_3$) of **1a**

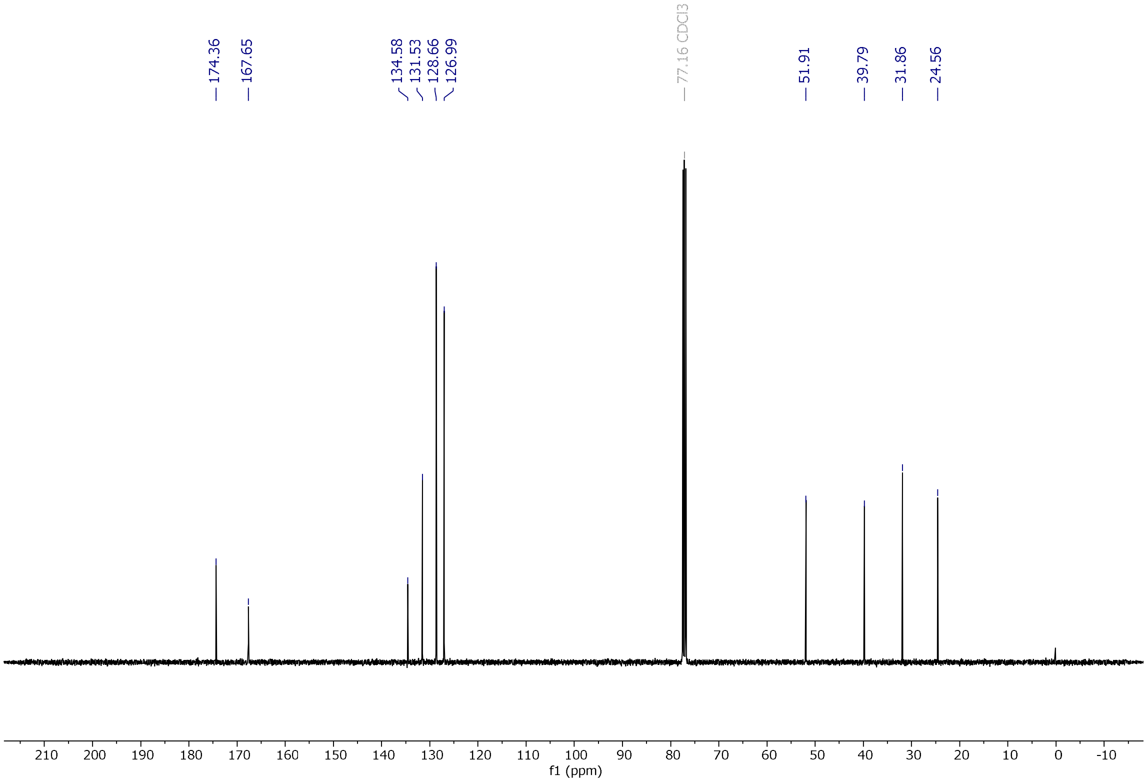

^{1}H NMR (400 MHz, $CDCl_3$) of **1b**

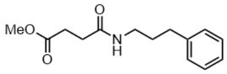

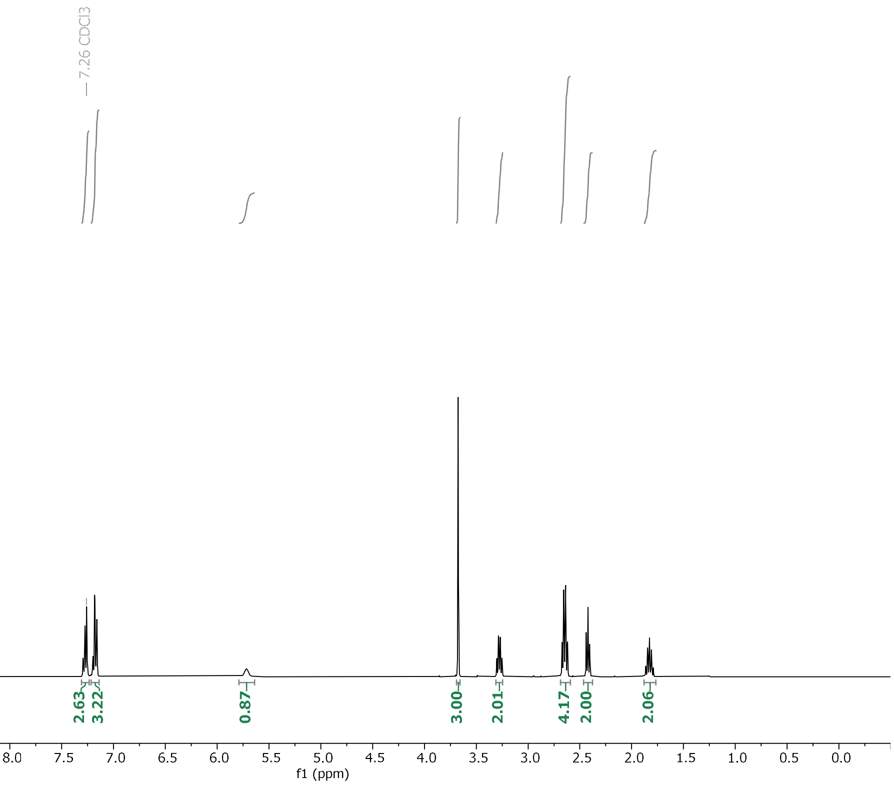

^{13}C NMR (101 MHz, $CDCl_3$) of **1b**

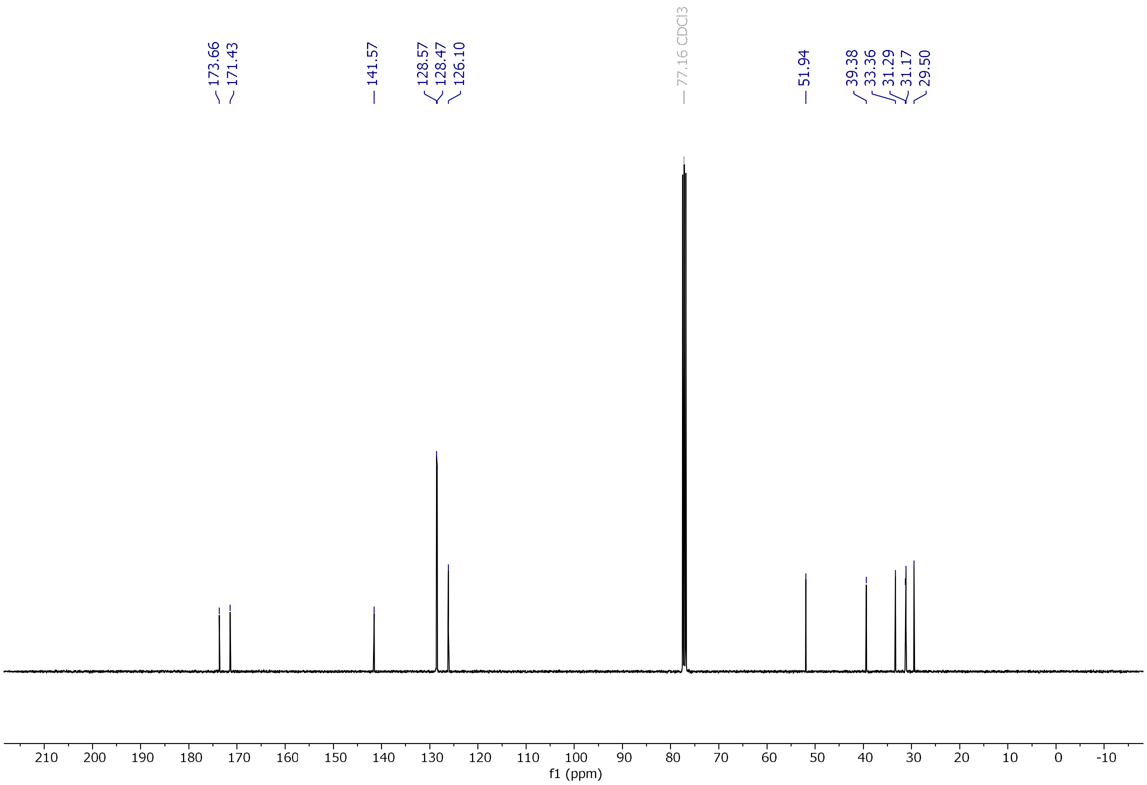

^{1}H NMR (400 MHz, $CDCl_3$) of **1c**

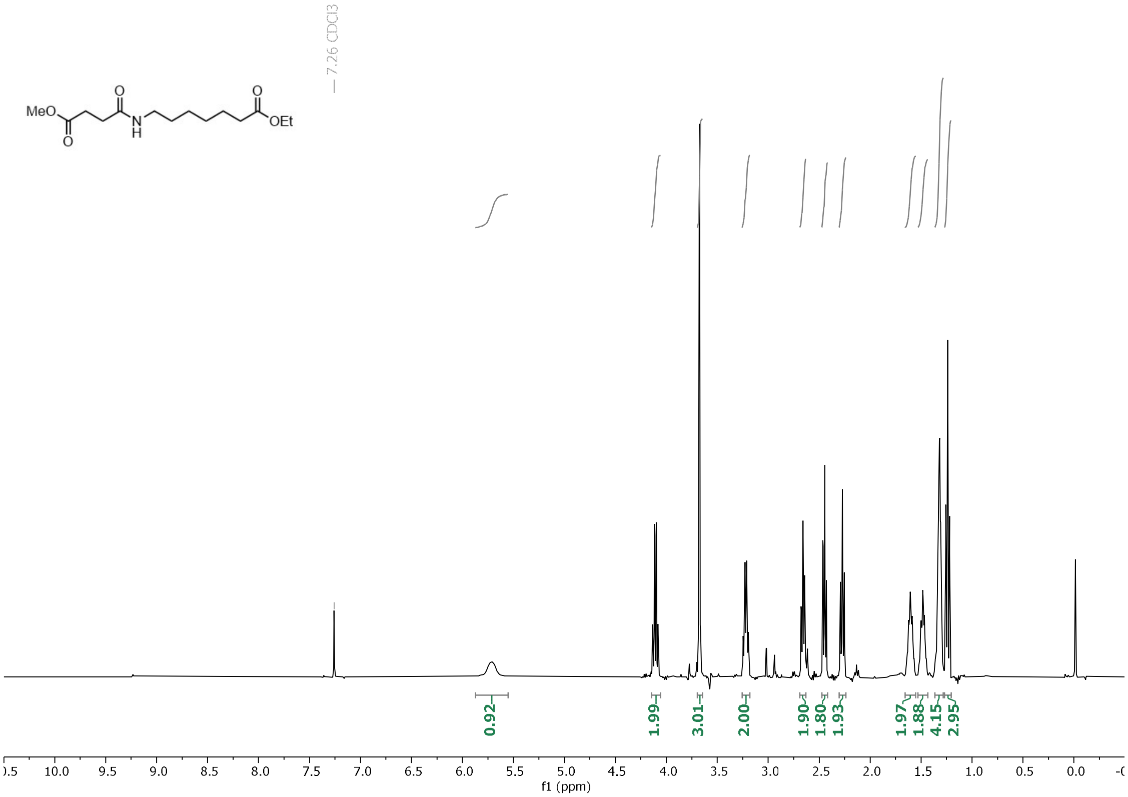

^{13}C NMR (101 MHz, $CDCl_3$) of **1c**

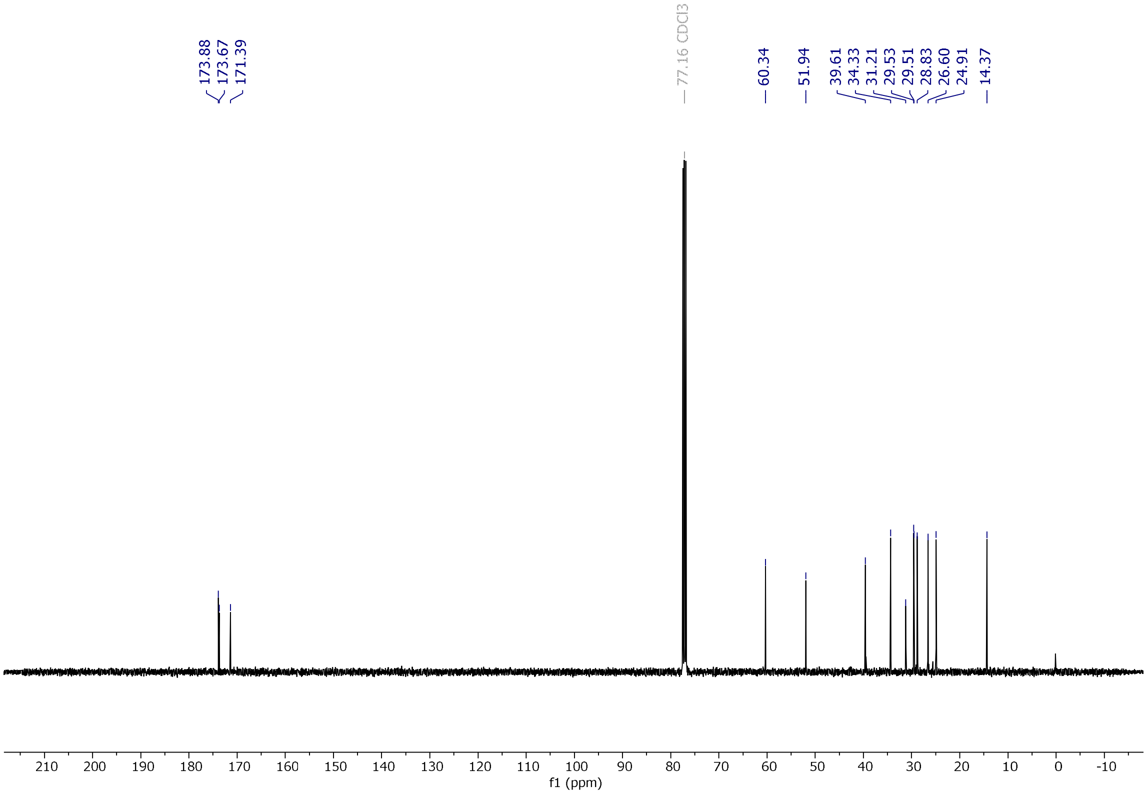

^{1}H NMR (400 MHz, $CDCl_3$) of **1d**

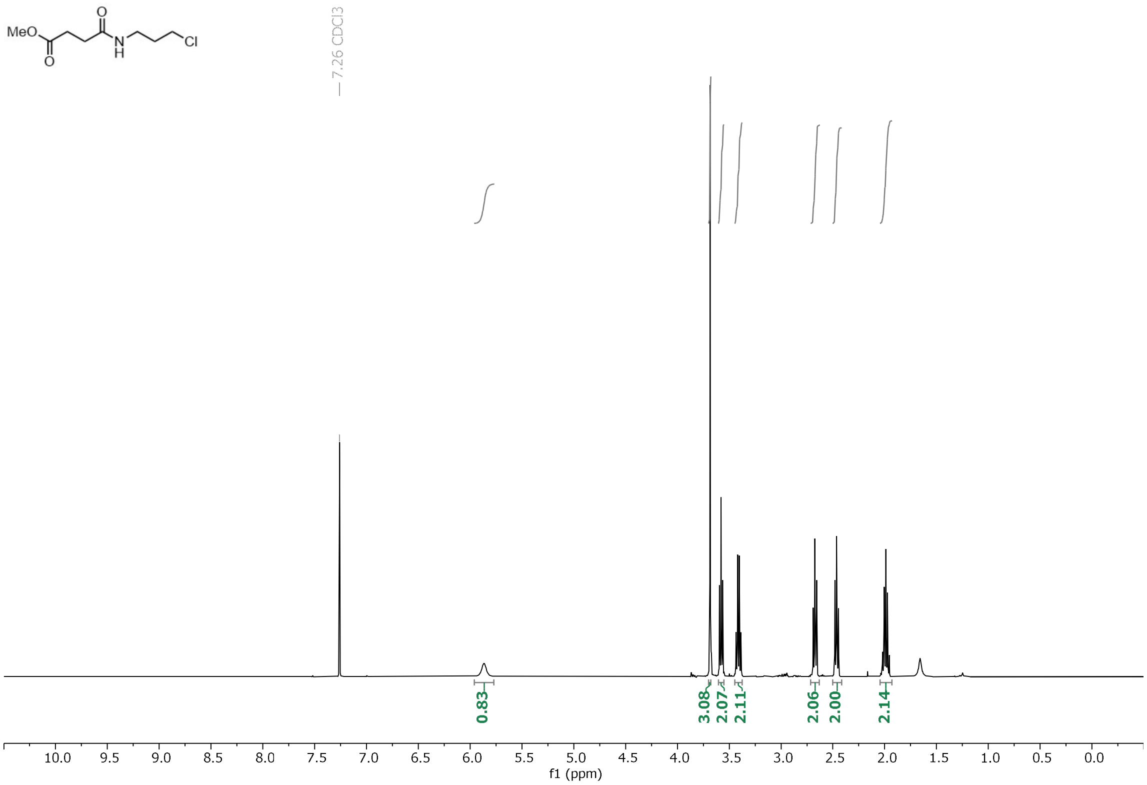

^{13}C NMR (101 MHz, $CDCl_3$) of **1d**

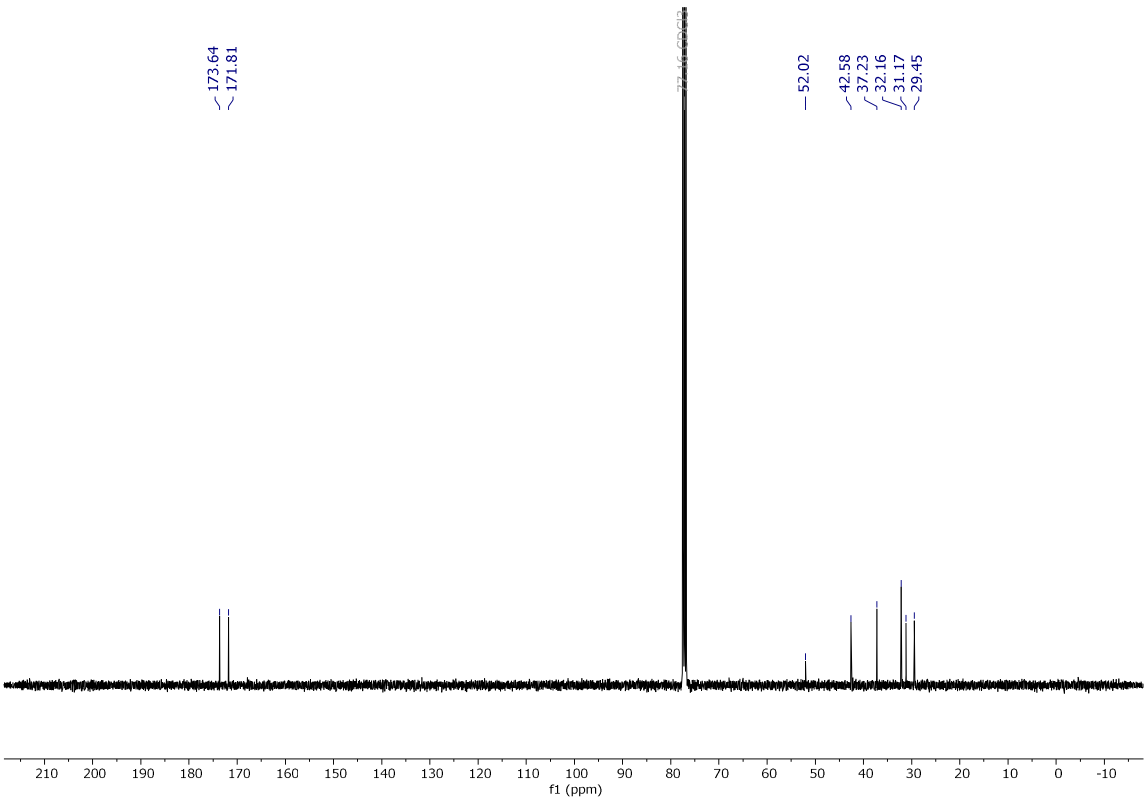

^{1}H NMR (300 MHz, $CDCl_3$) of **1e**

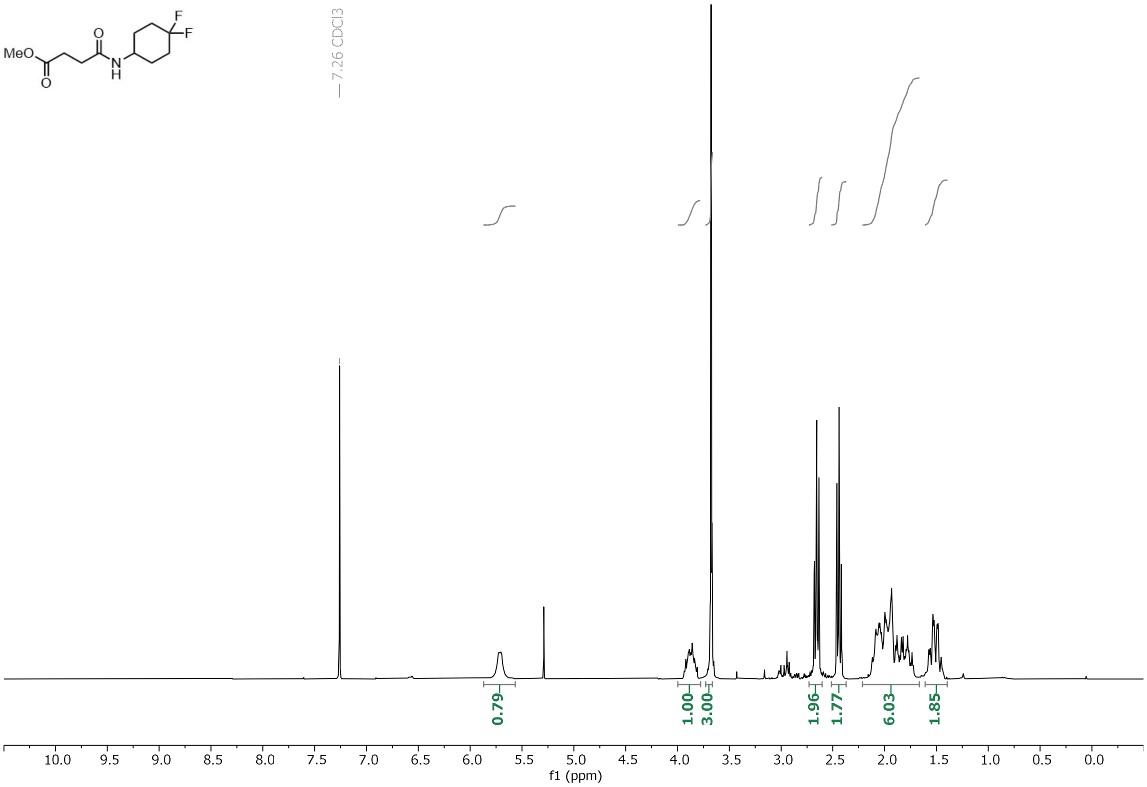

^{13}C NMR (101 MHz, $CDCl_3$) of **1e**

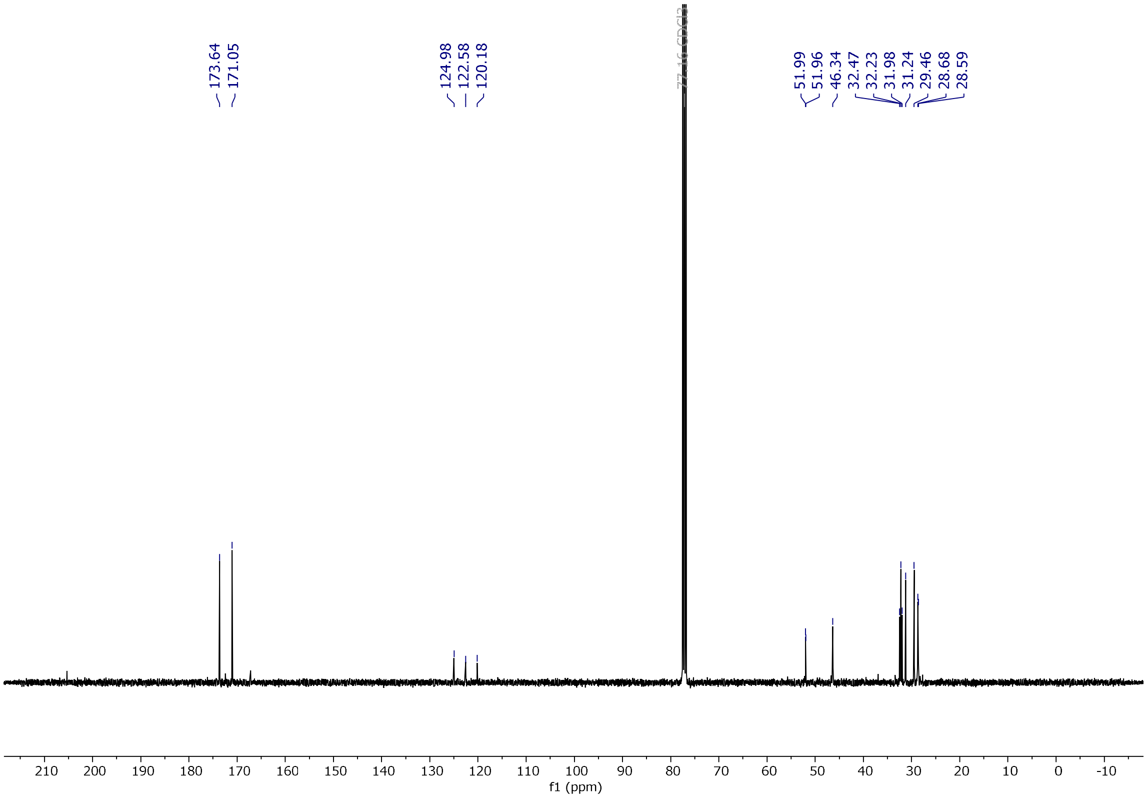

^{19}F NMR (282 MHz, $CDCl_3$) of **1e**

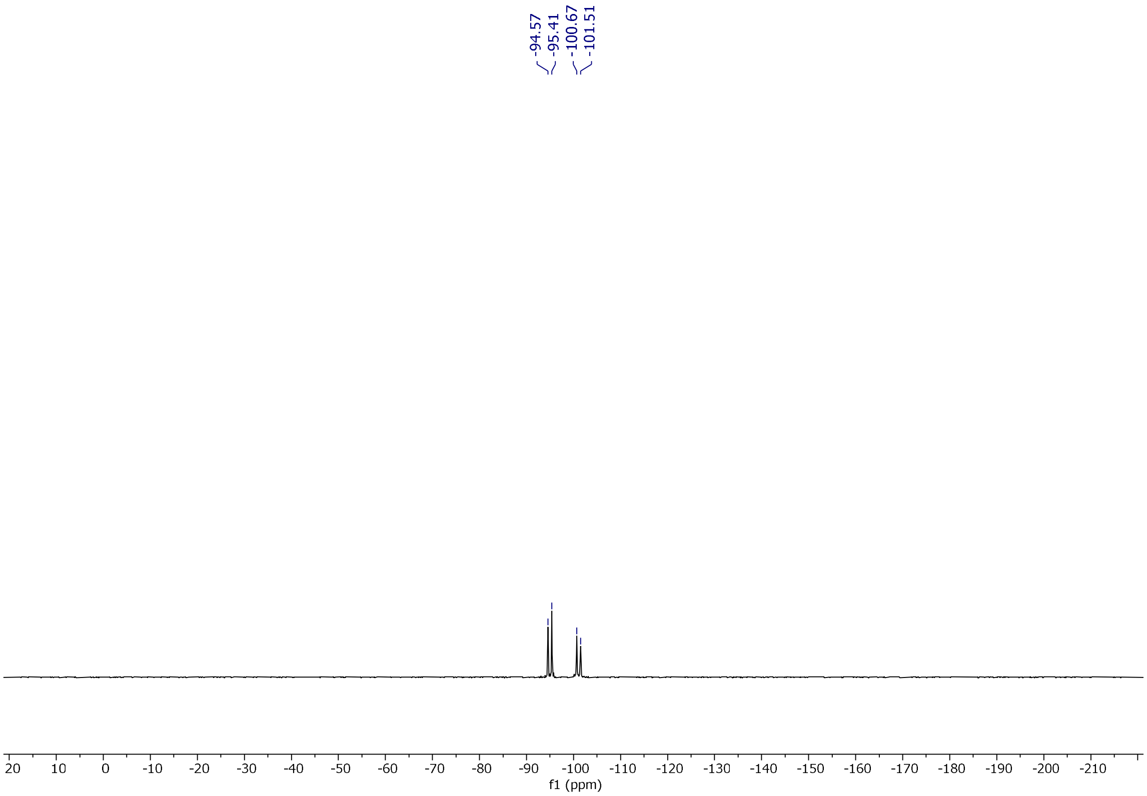

^{1}H NMR (400 MHz, $CDCl_3$) of **1f**

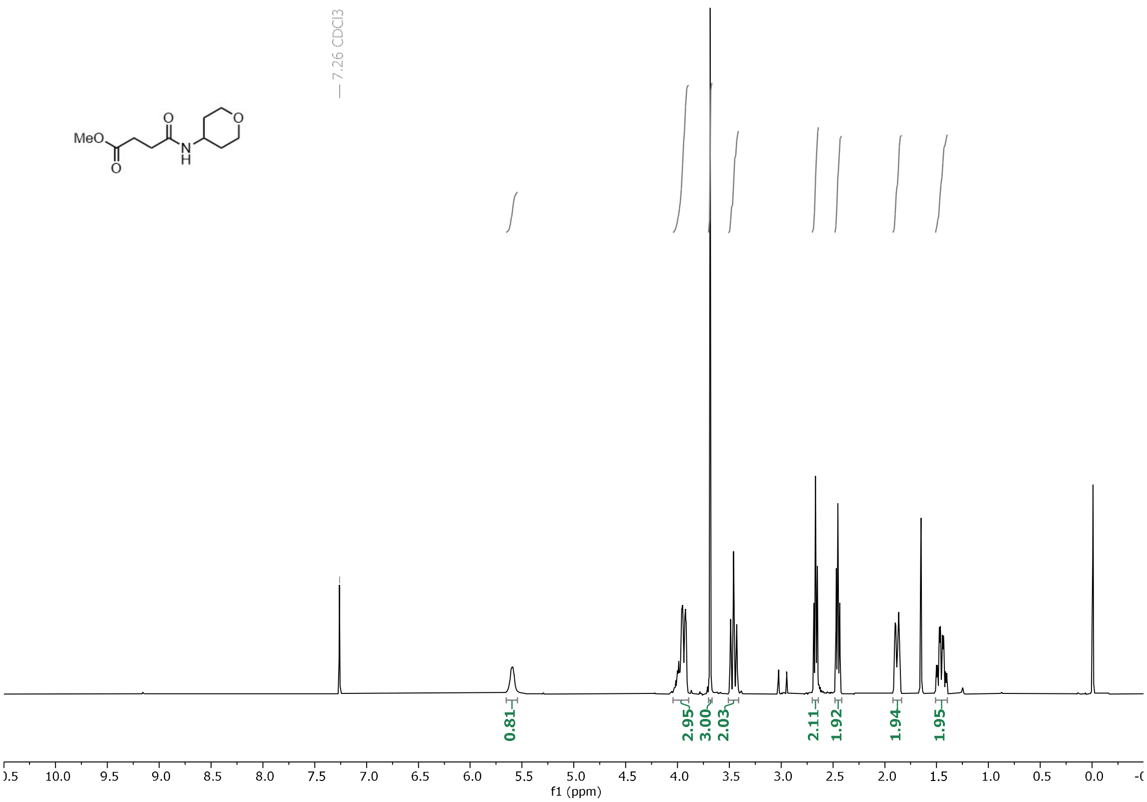

^{13}C NMR (101 MHz, $CDCl_3$) of **1f**

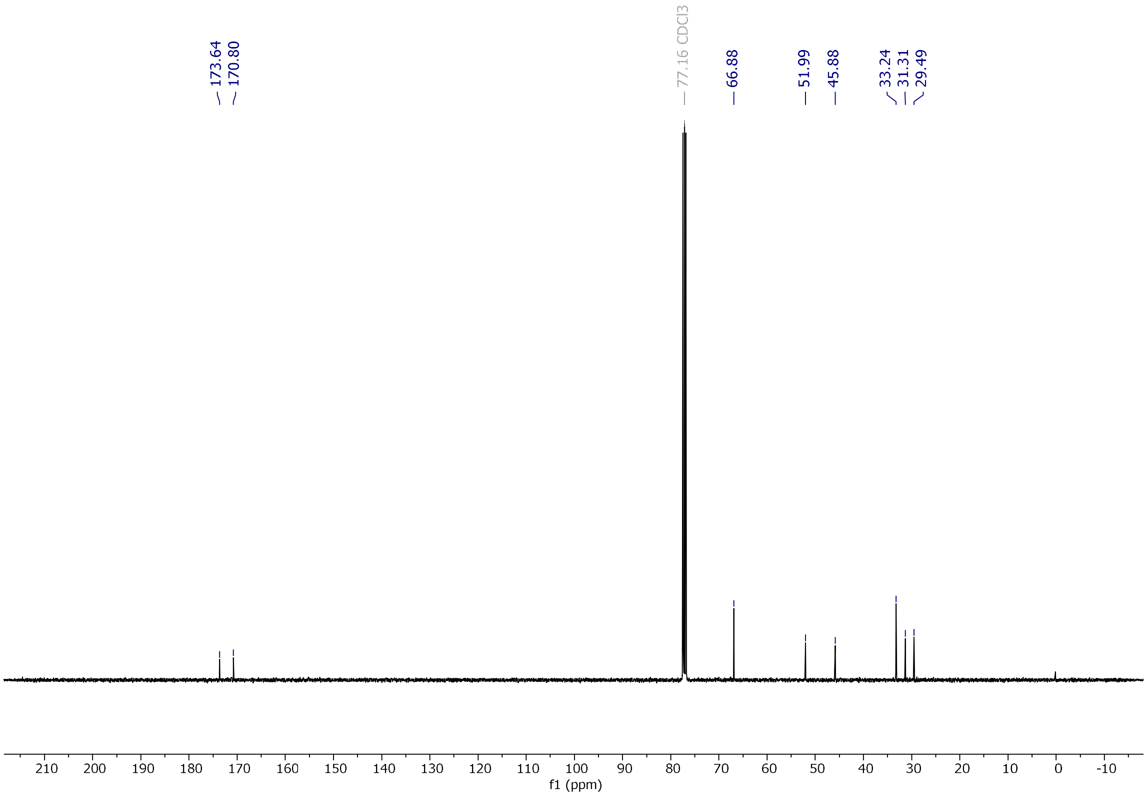

^{1}H NMR (300 MHz, $CDCl_3$) of **1g**

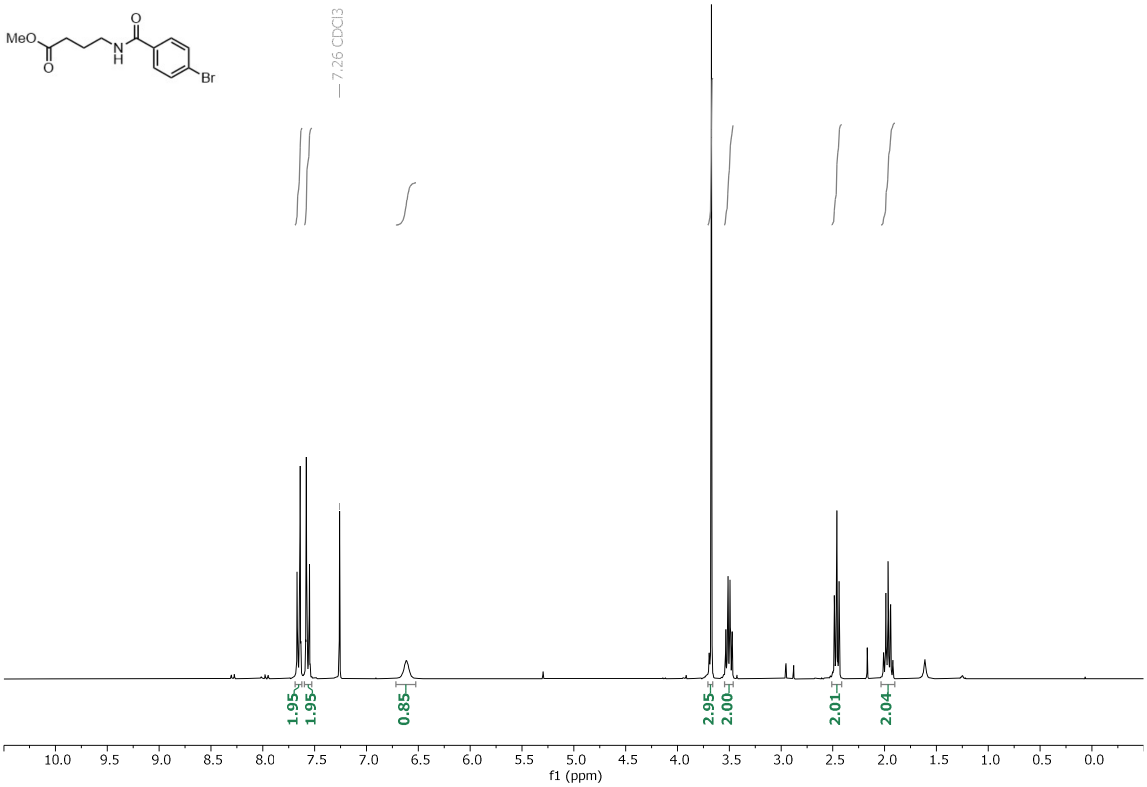

^{13}C NMR (101 MHz, $CDCl_3$) of **1g**

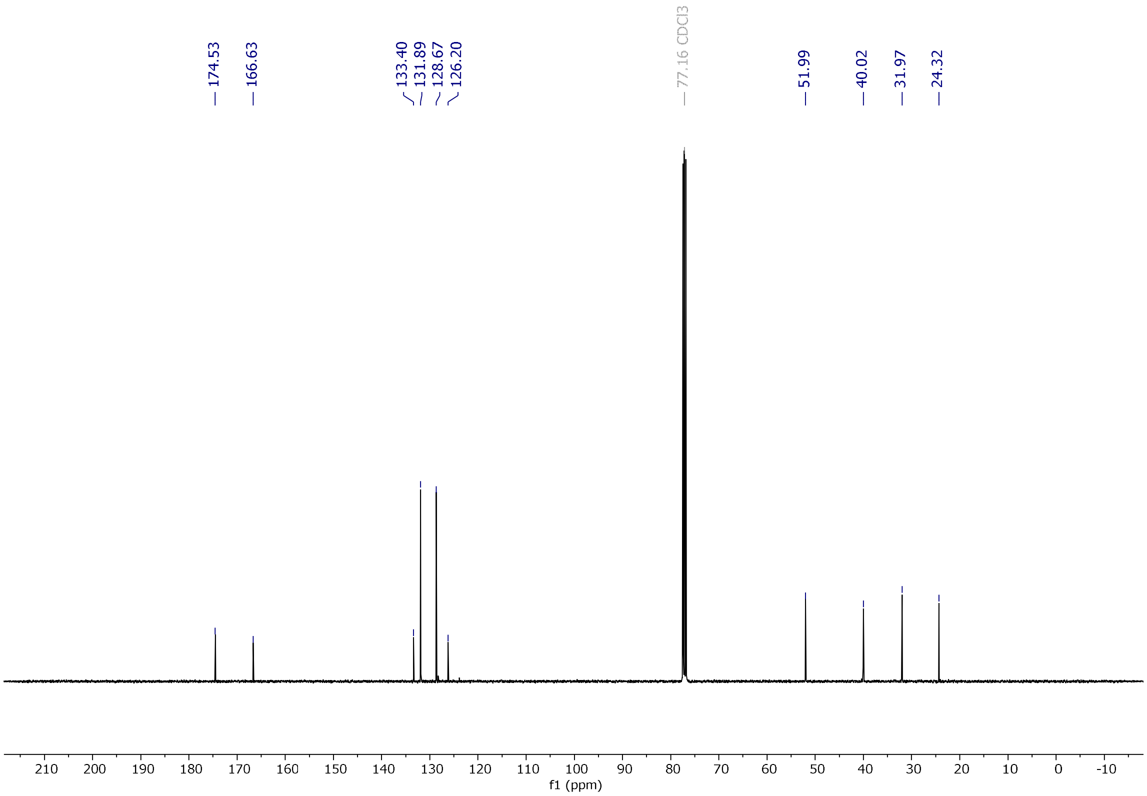

^{1}H NMR (400 MHz, $CDCl_3$) of **1h**

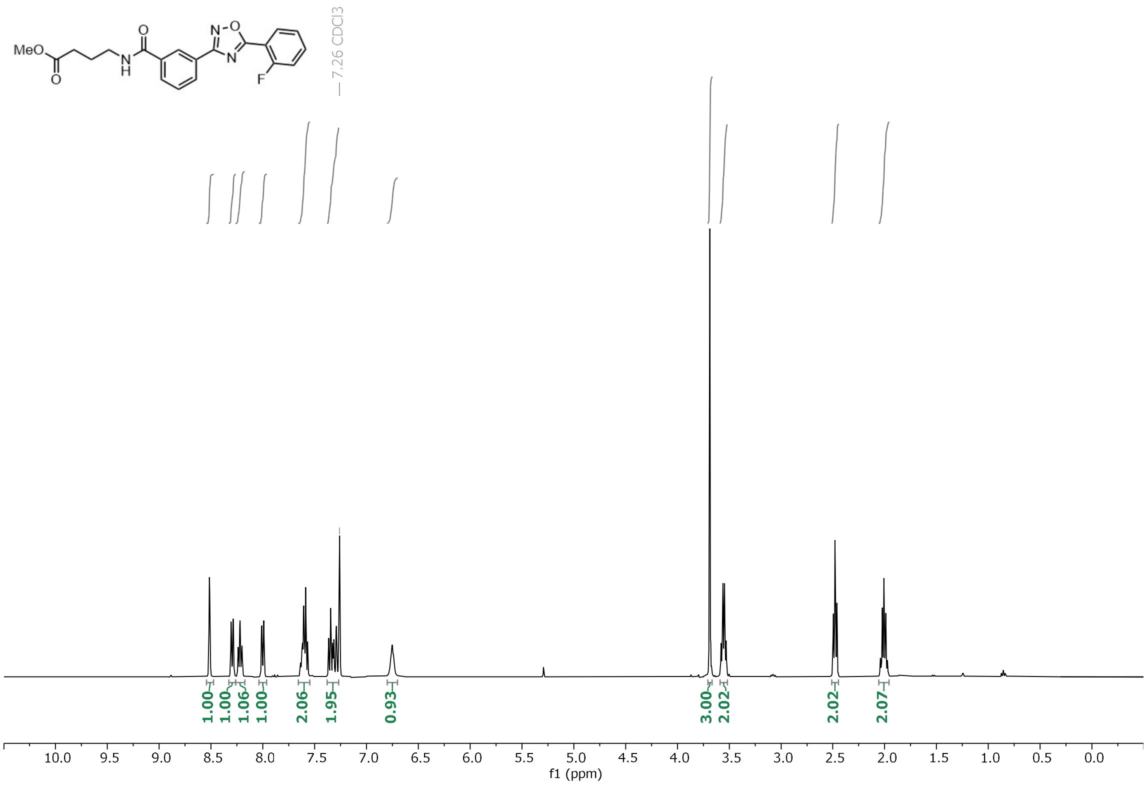

^{13}C NMR (101 MHz, $CDCl_3$) of **1h**

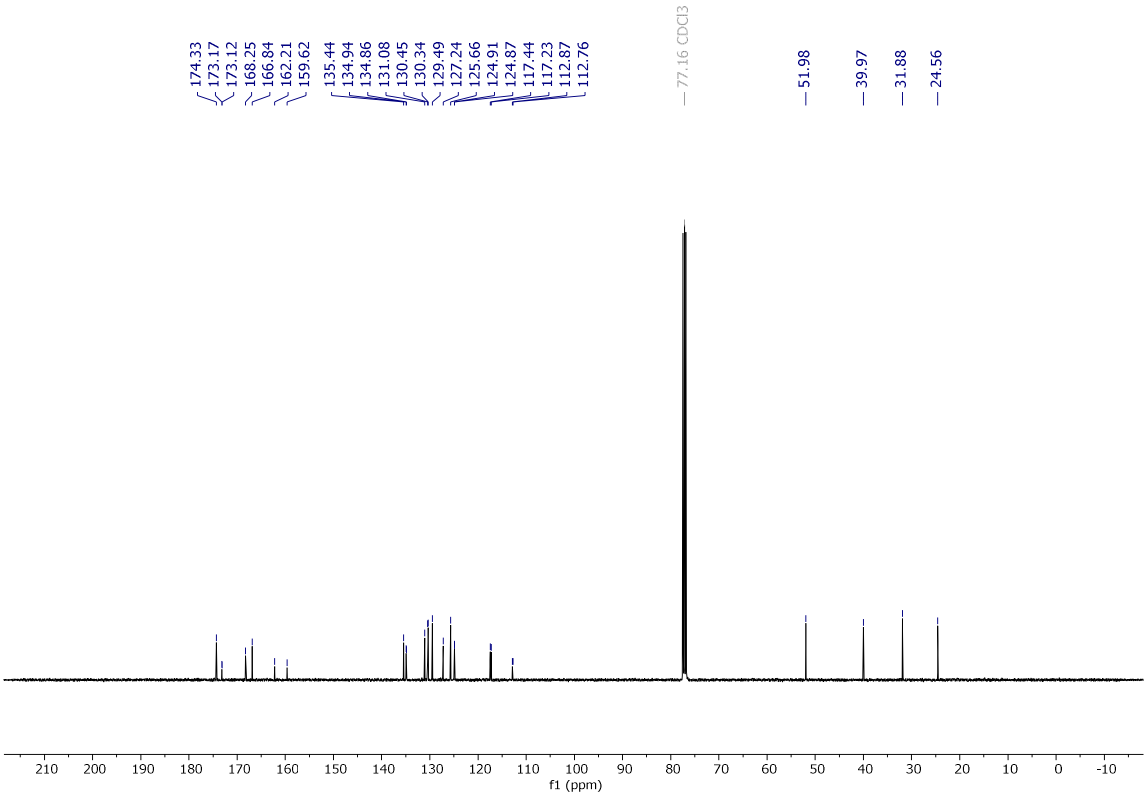

^{19}F NMR (282 MHz, $CDCl_3$) of **1h**

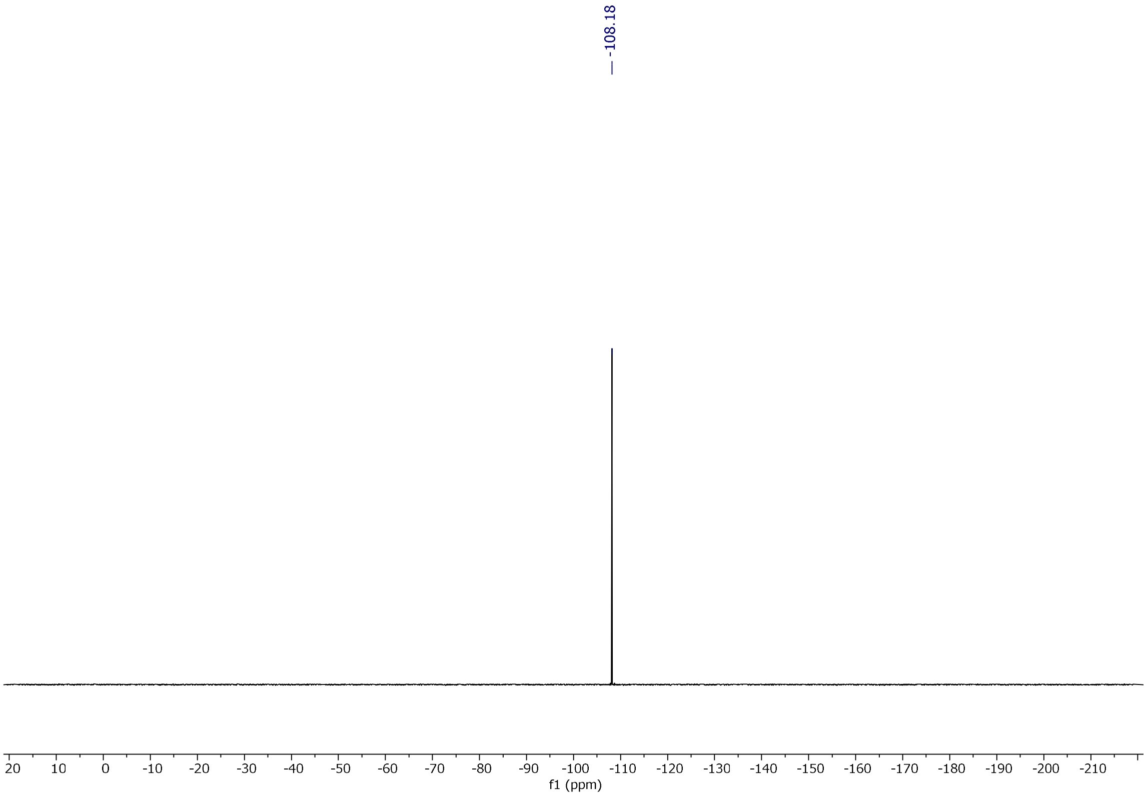

^{1}H NMR (400 MHz, $CDCl_3$) of **1i**

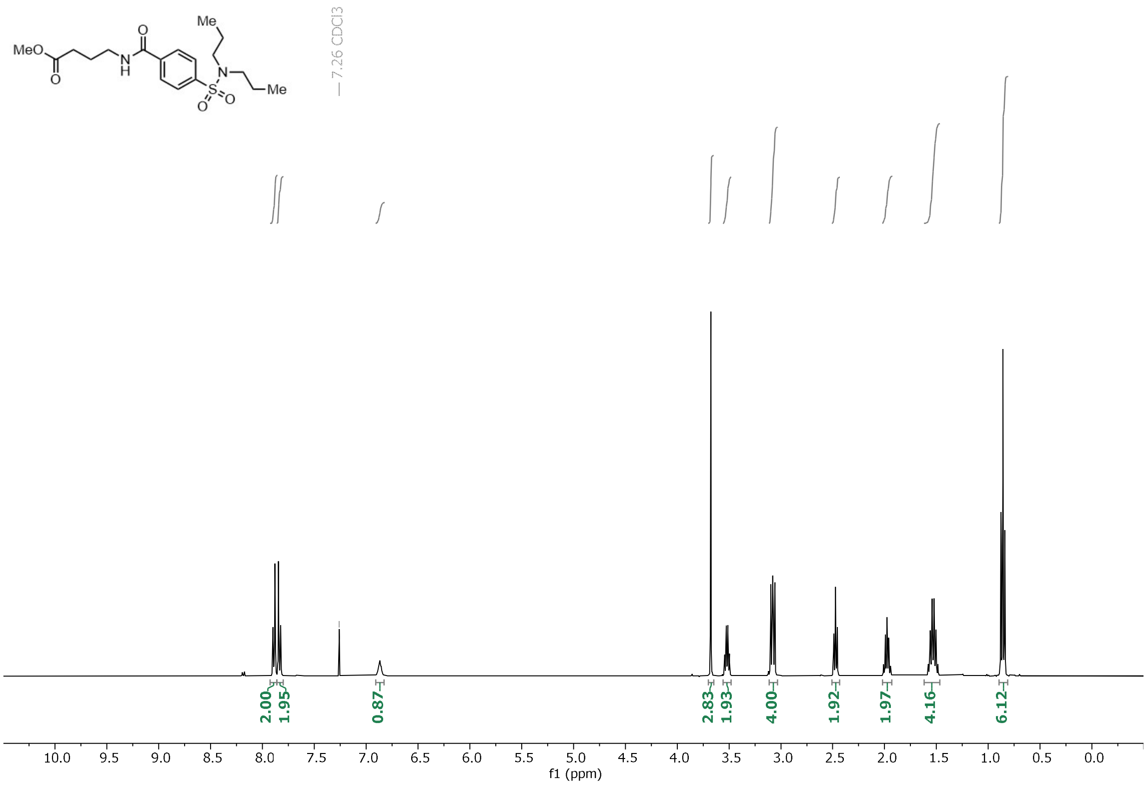

^{13}C NMR (101 MHz, $CDCl_3$) of **1i**

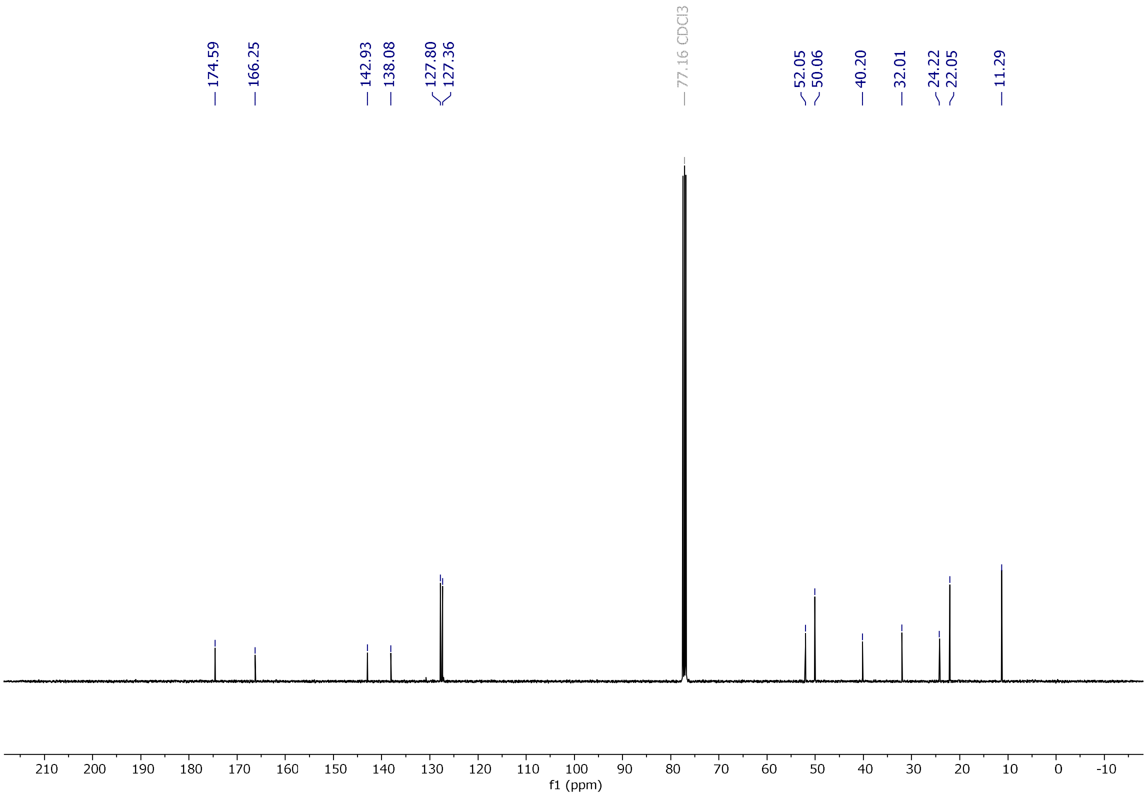

^{1}H NMR (300 MHz, $CDCl_3$) of **1j**

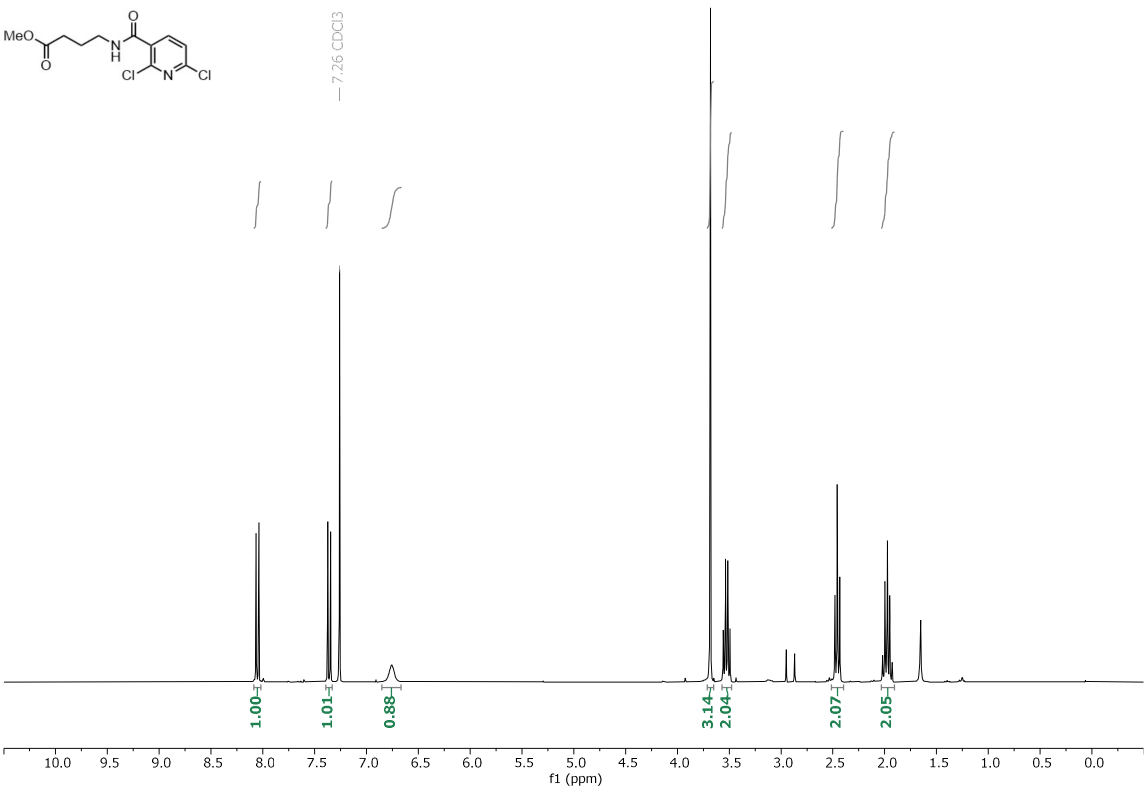

^{13}C NMR (101 MHz, $CDCl_3$) of **1j**

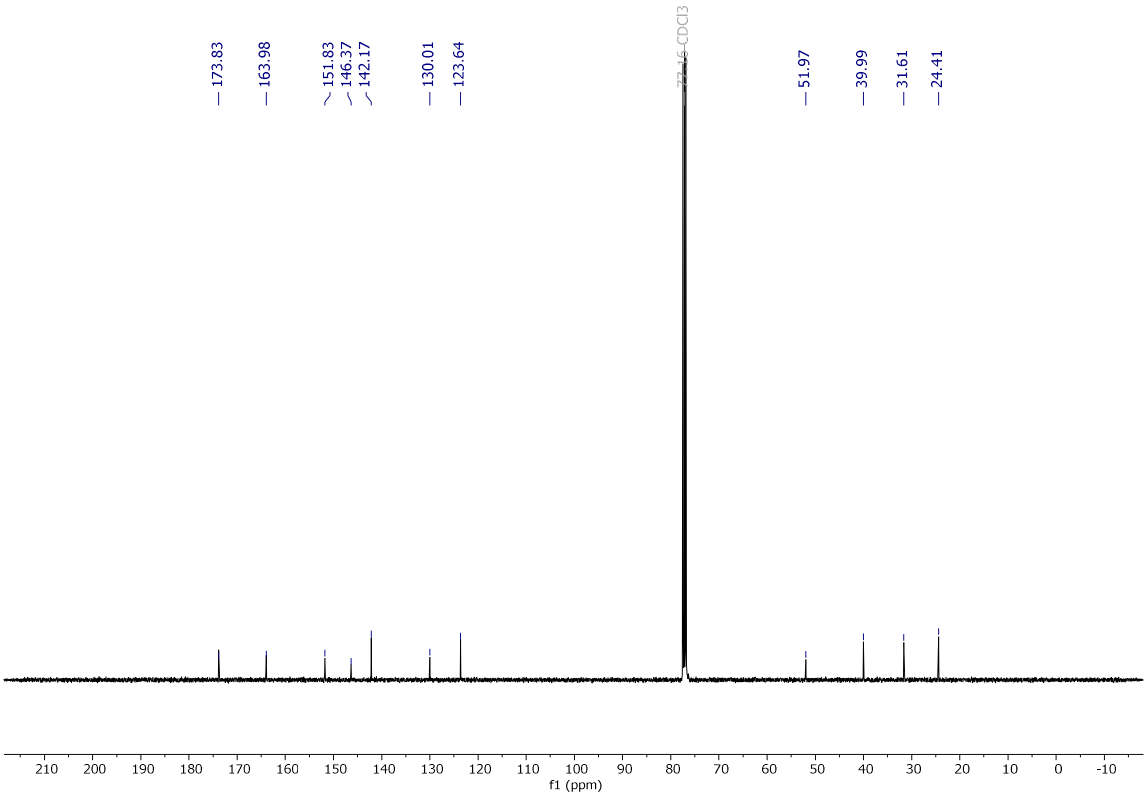

^{1}H NMR (300 MHz, $CDCl_3$) of **1k**

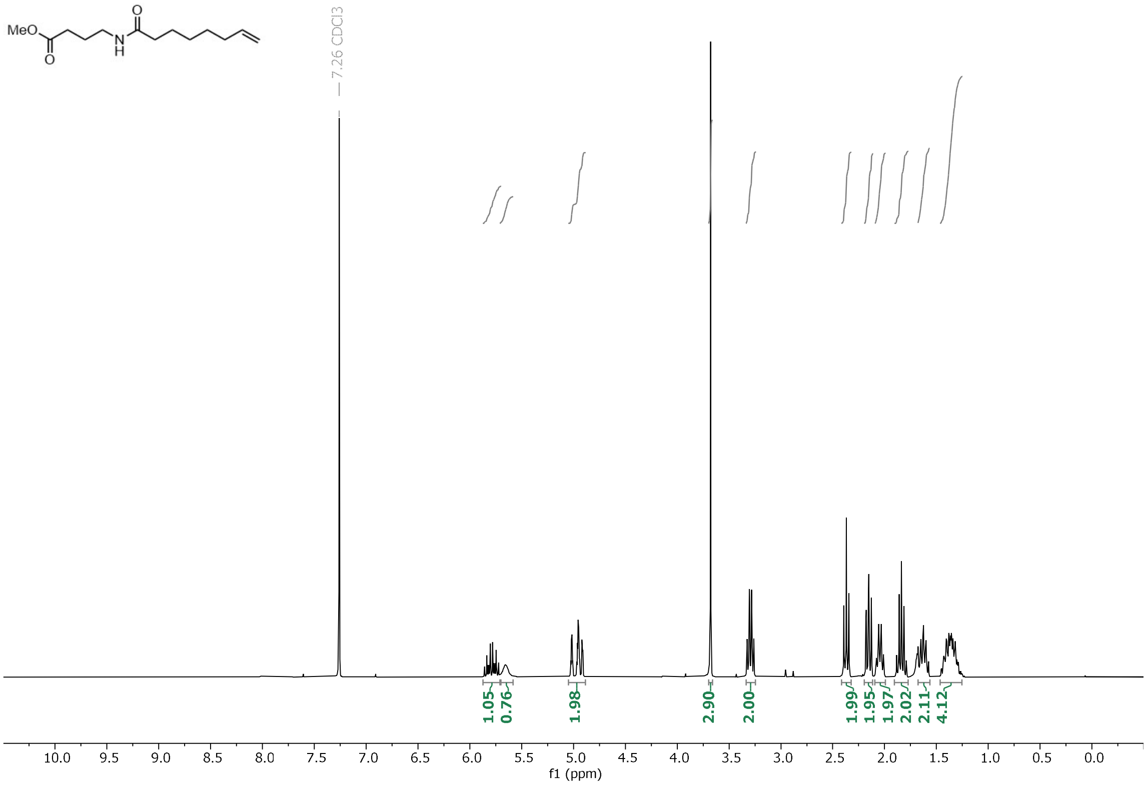

^{13}C NMR (101 MHz, $CDCl_3$) of **1k**

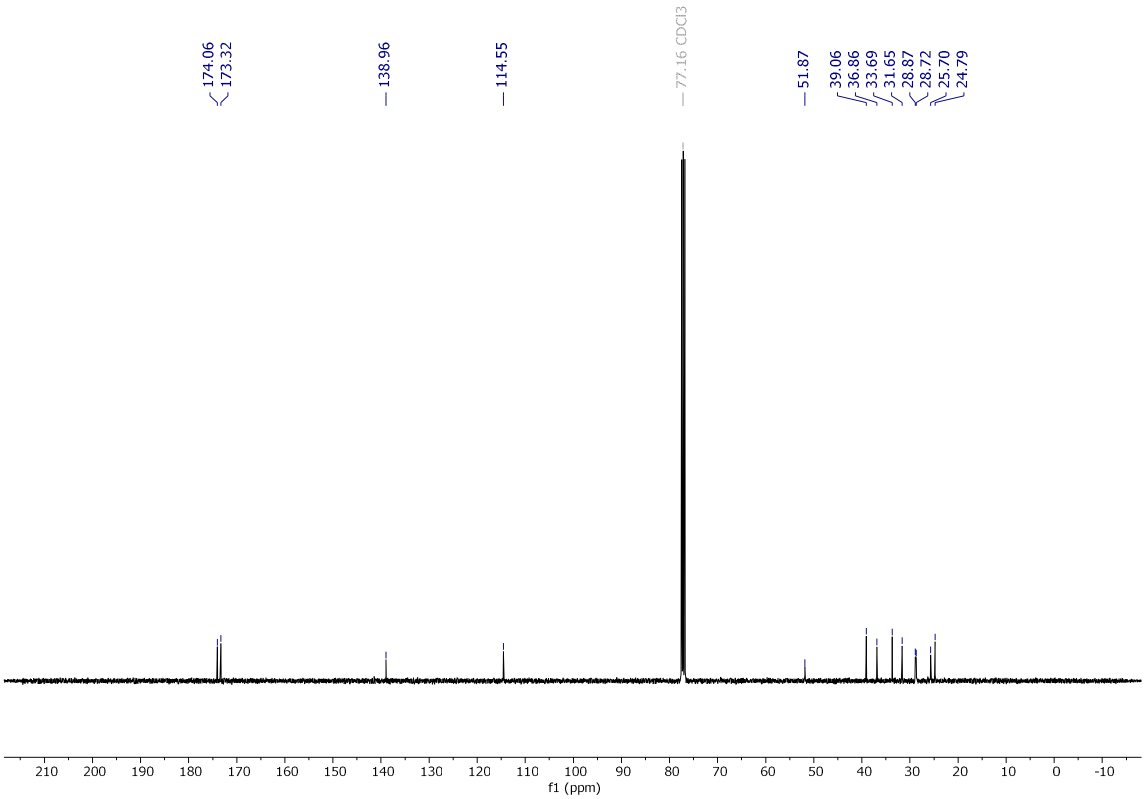

^{1}H NMR (400 MHz, $CDCl_3$) of **1l**

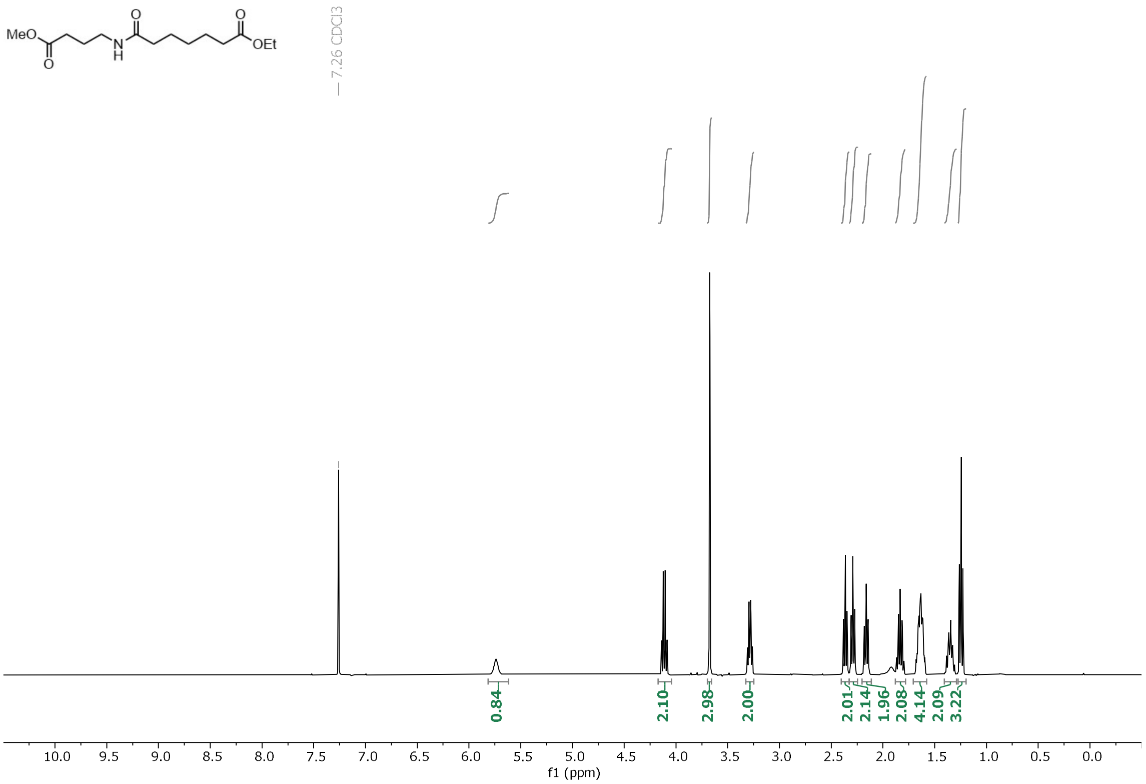

^{13}C NMR (101 MHz, $CDCl_3$) of **1l**

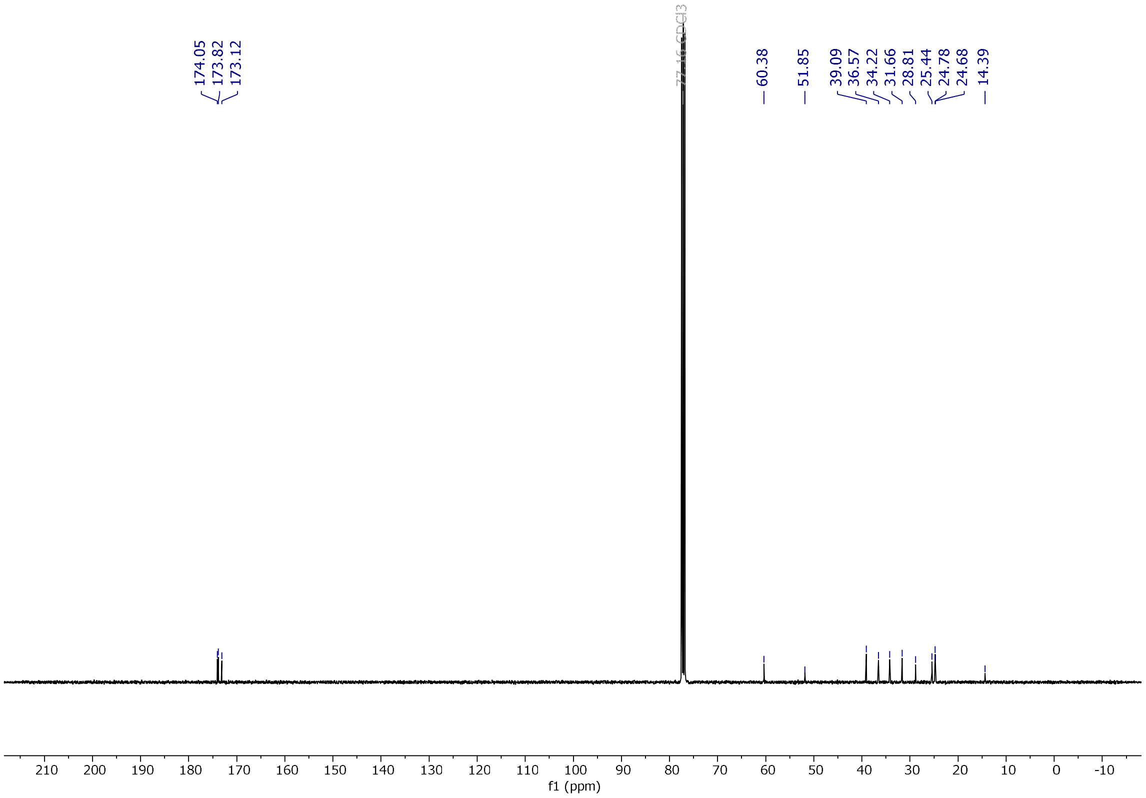

^{1}H NMR (400 MHz, $CDCl_3$) of **1m**

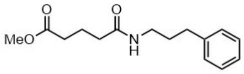

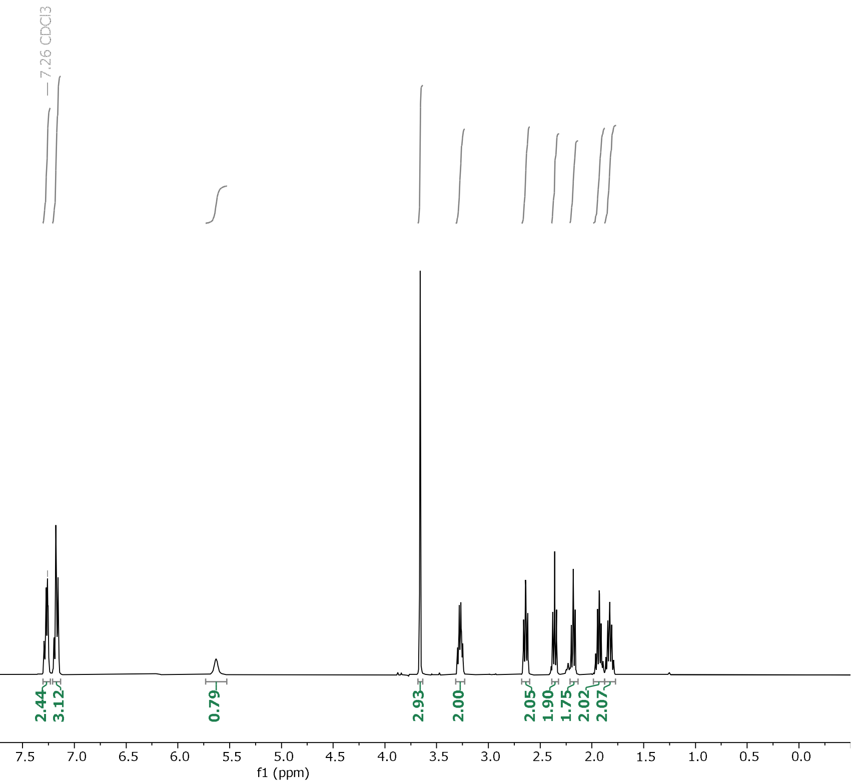

^{13}C NMR (101 MHz, $CDCl_3$) of **1m**

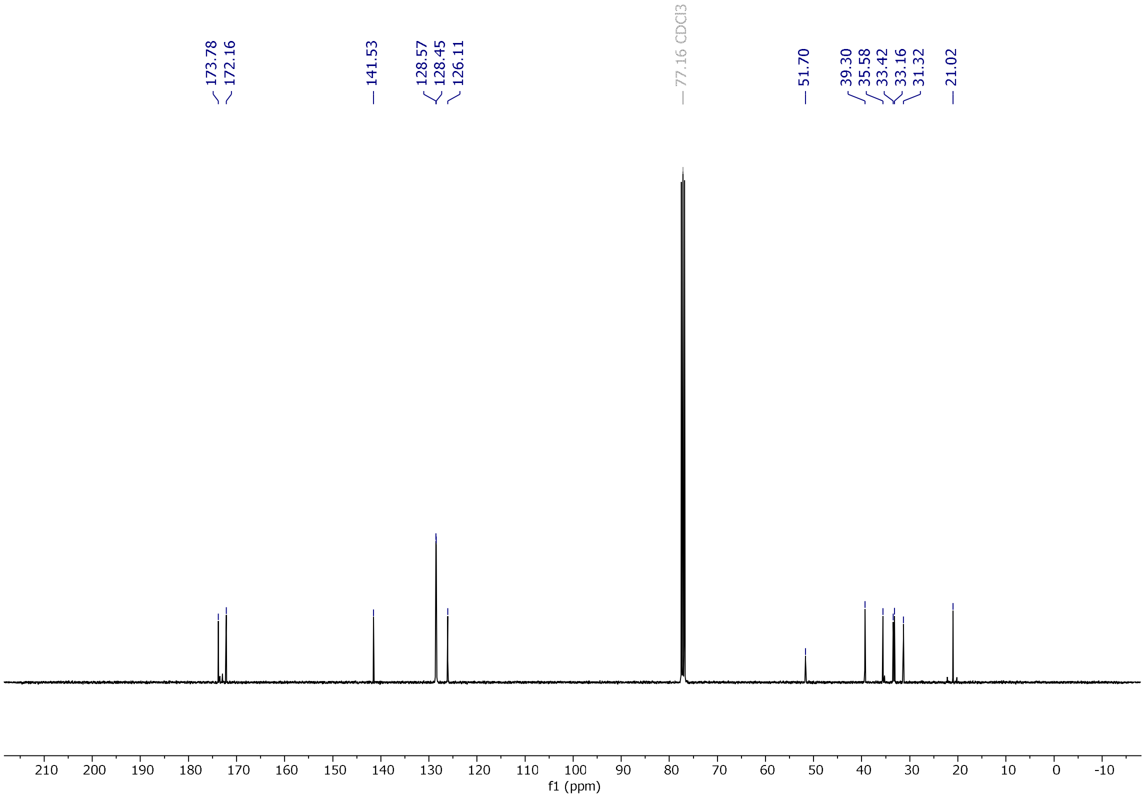

^1H NMR (400 MHz, $CDCl_3$) of **1n**

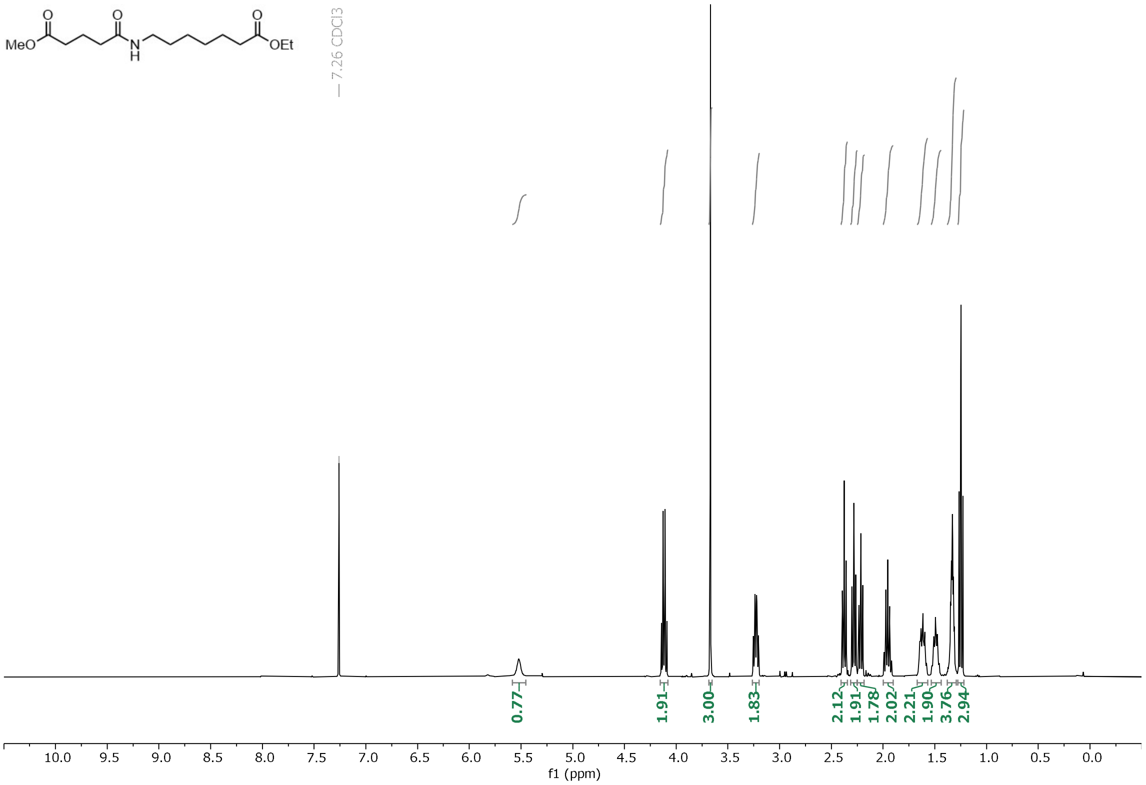

^{13}C NMR (101 MHz, $CDCl_3$) of **1n**

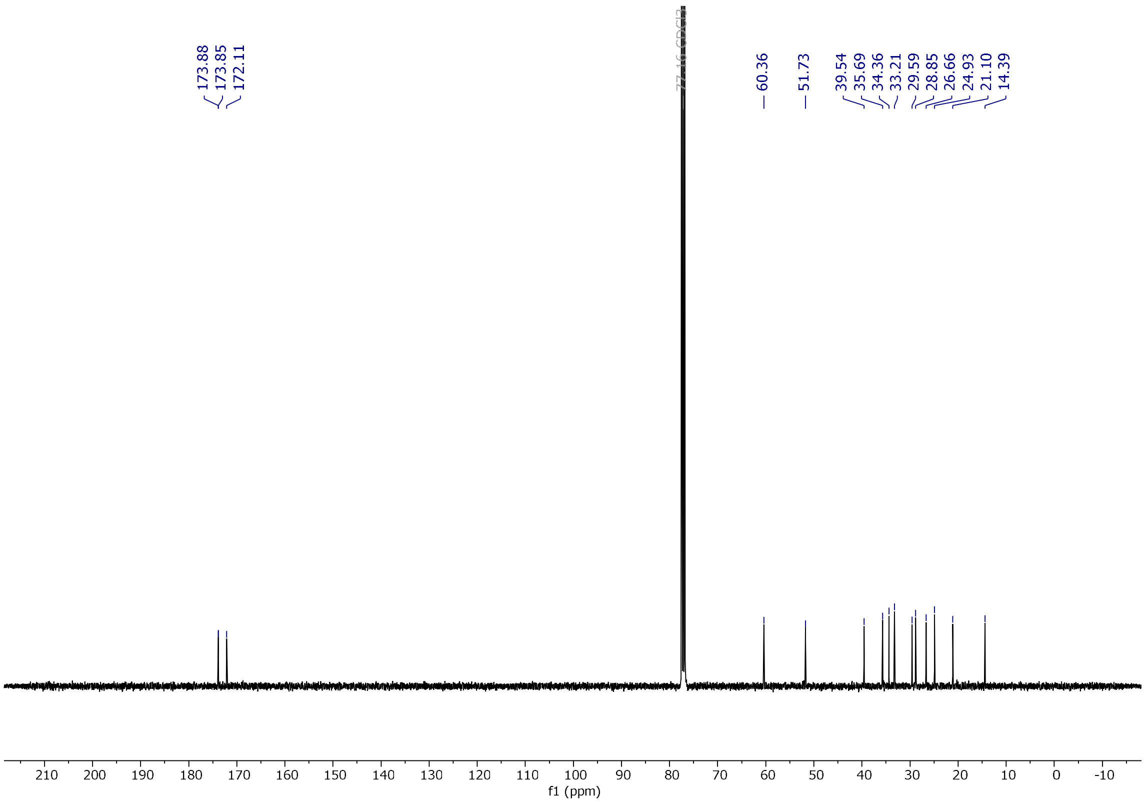

^1H NMR (300 MHz, $CDCl_3$) of **1o**

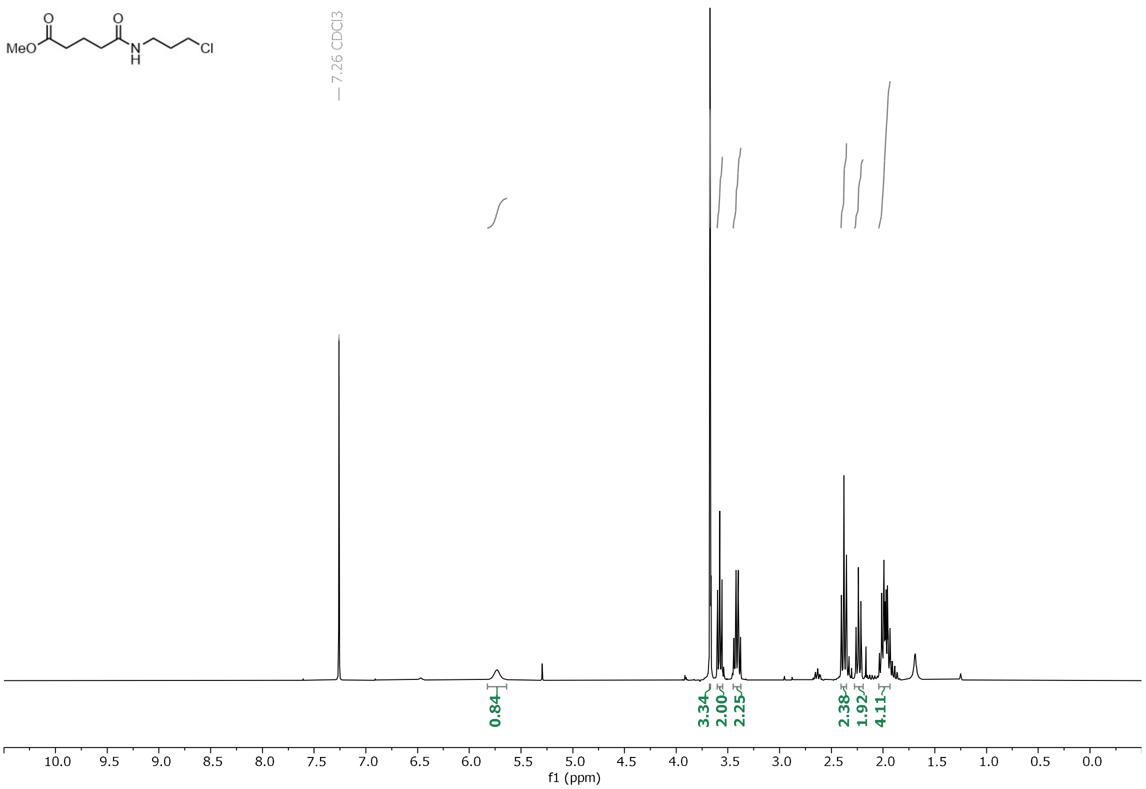

^{13}C NMR (101 MHz, $CDCl_3$) of **1o**

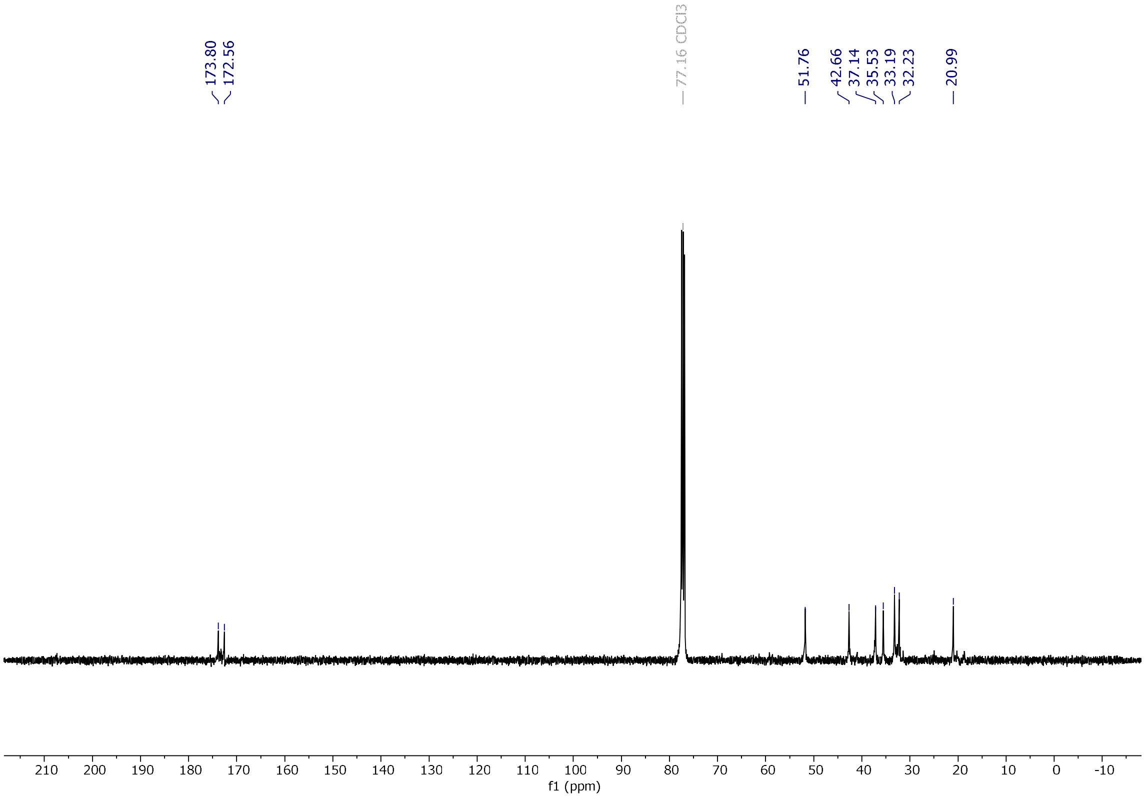

^{1}H NMR (300 MHz, $CDCl_3$) of **1r**

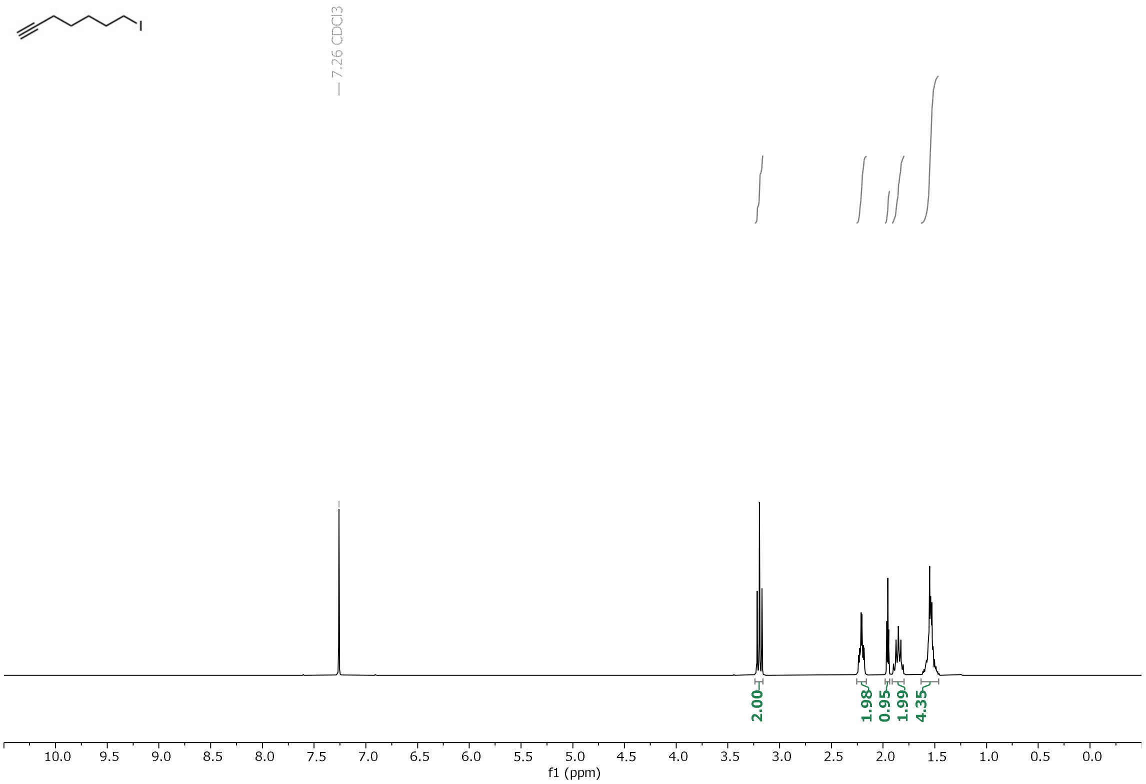

^{1}H NMR (300 MHz, $CDCl_3$) of **1s**

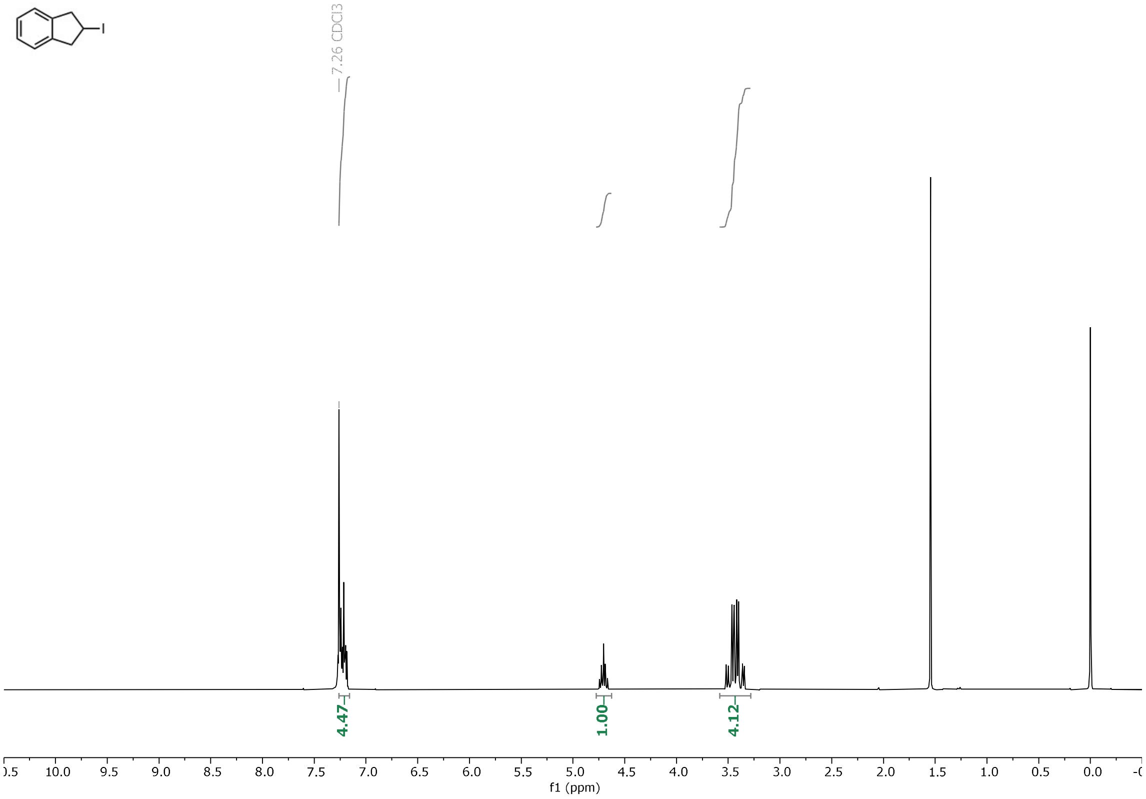

14.NMR spectra of products

^{1}H NMR (300 MHz, $CDCl_3$) of **2**

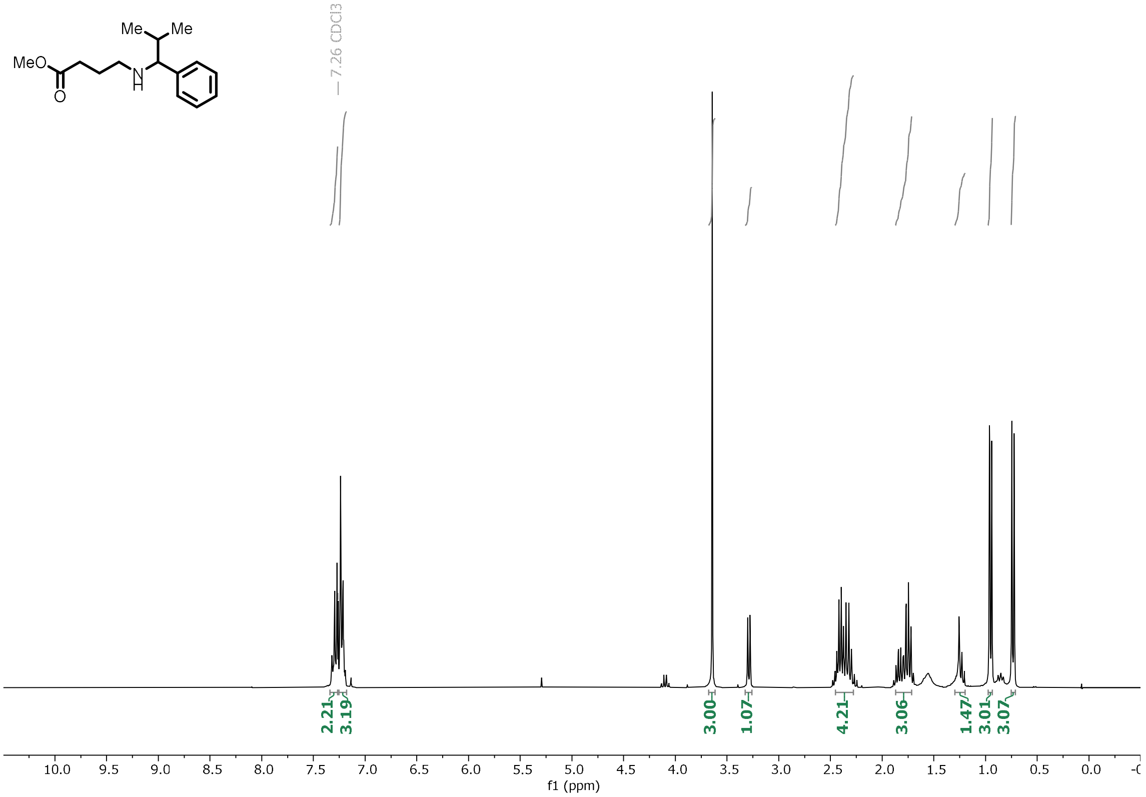

^{13}C NMR (75 MHz, $CDCl_3$) of **2**

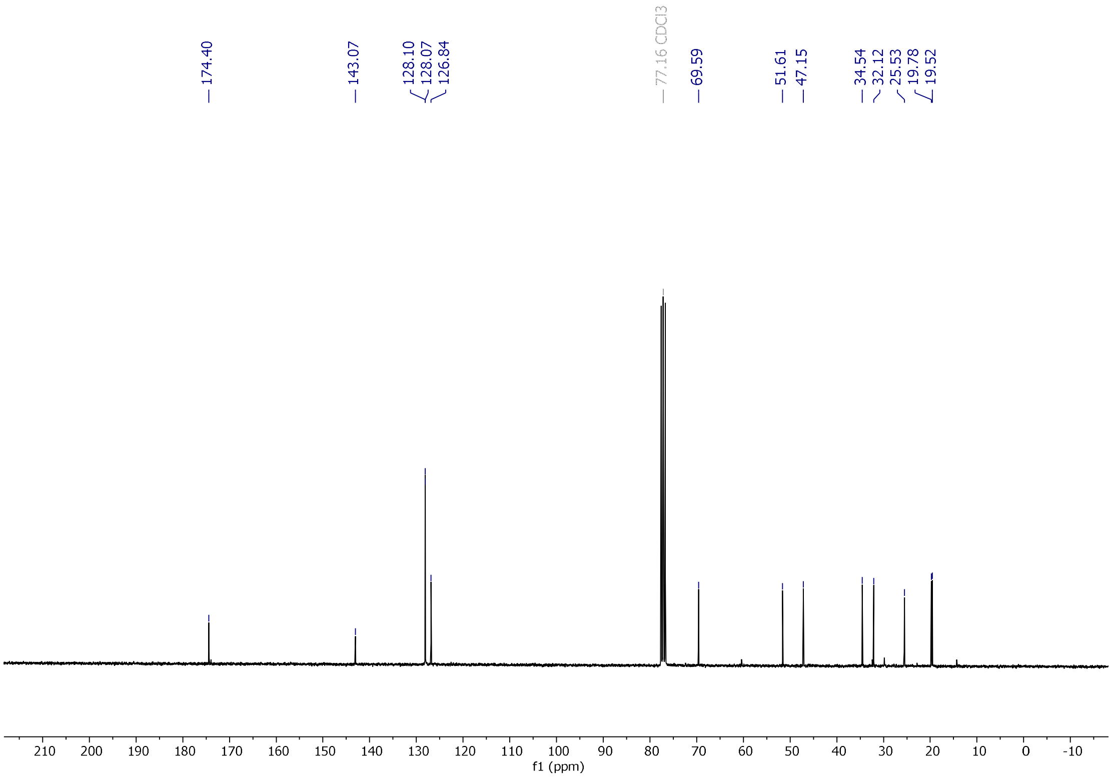

^{1}H NMR (400 MHz, $CDCl_3$) of **4**

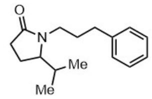

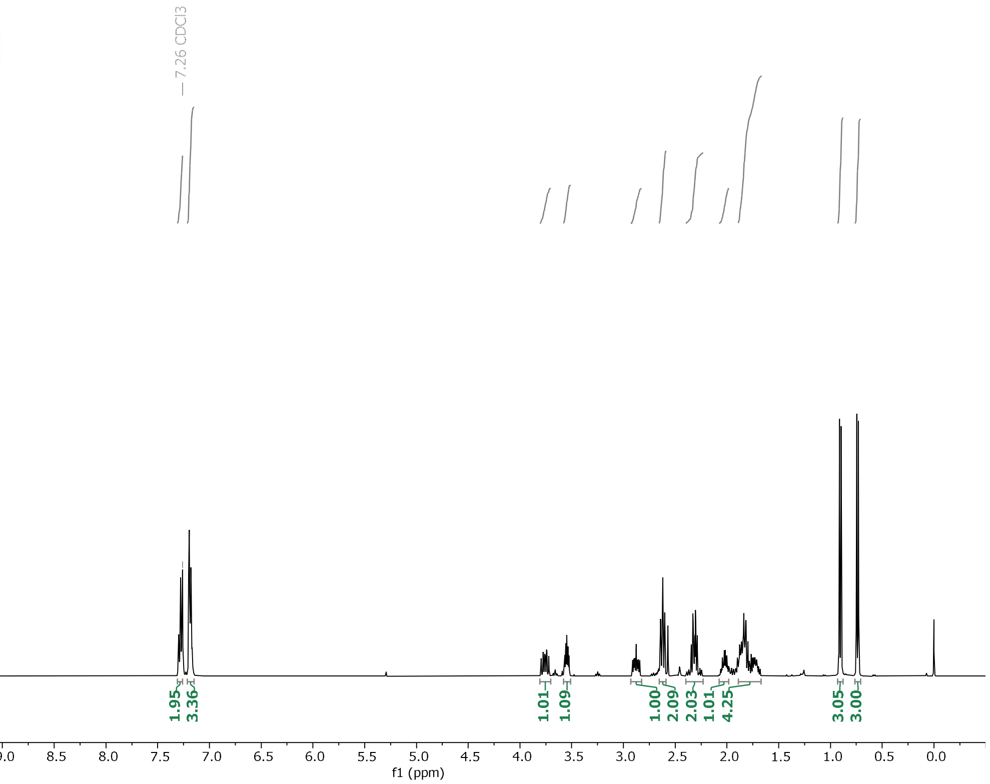

^{13}C NMR (101 MHz, $CDCl_3$) of **4**

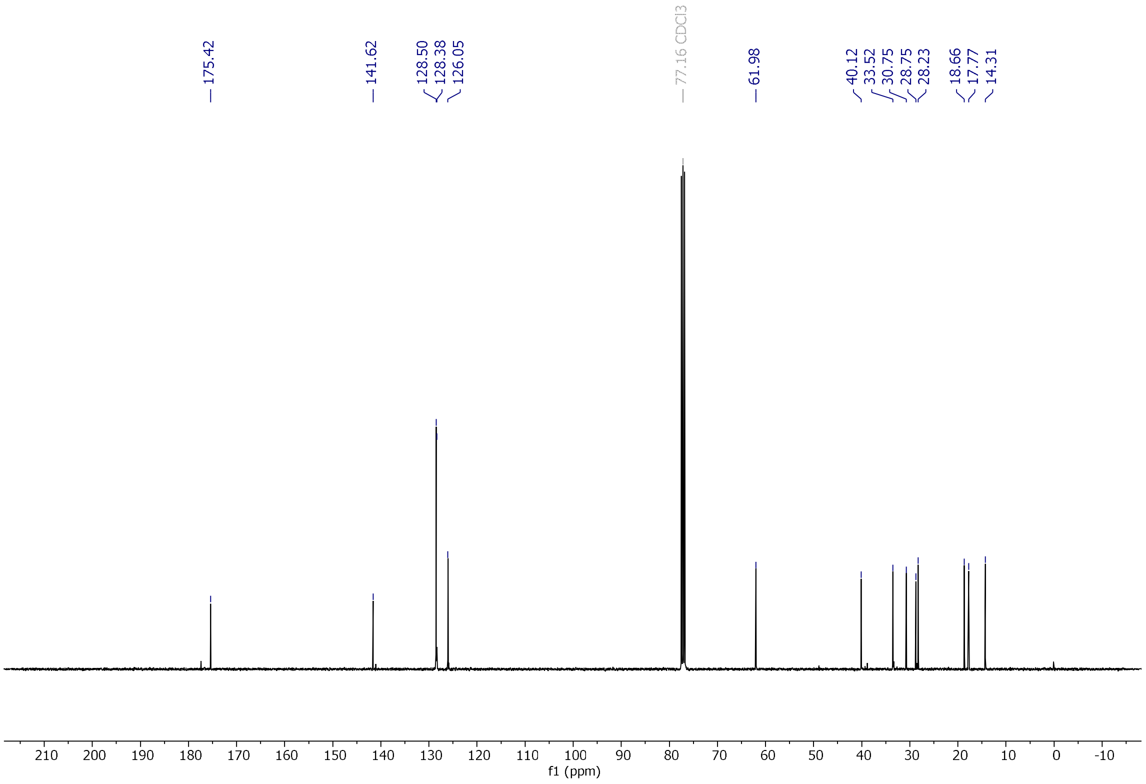

^{1}H NMR (400 MHz, $CDCl_3$) of **5**

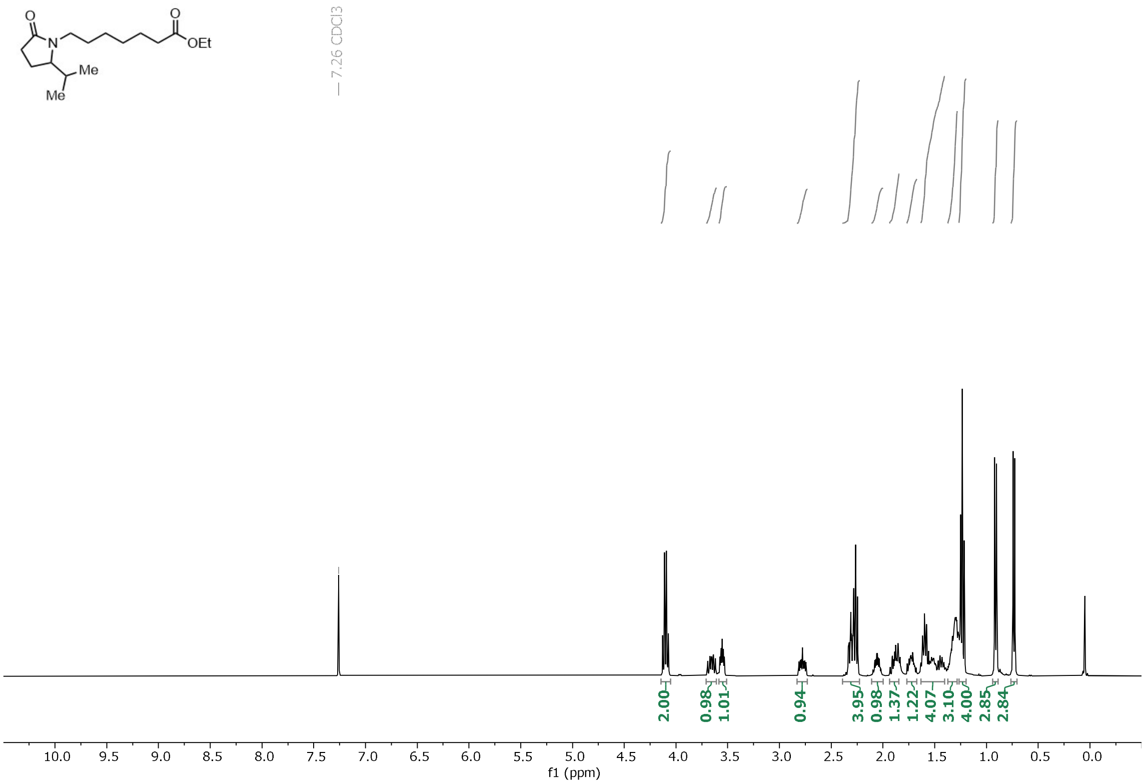

^{13}C NMR (101 MHz, $CDCl_3$) of **5**

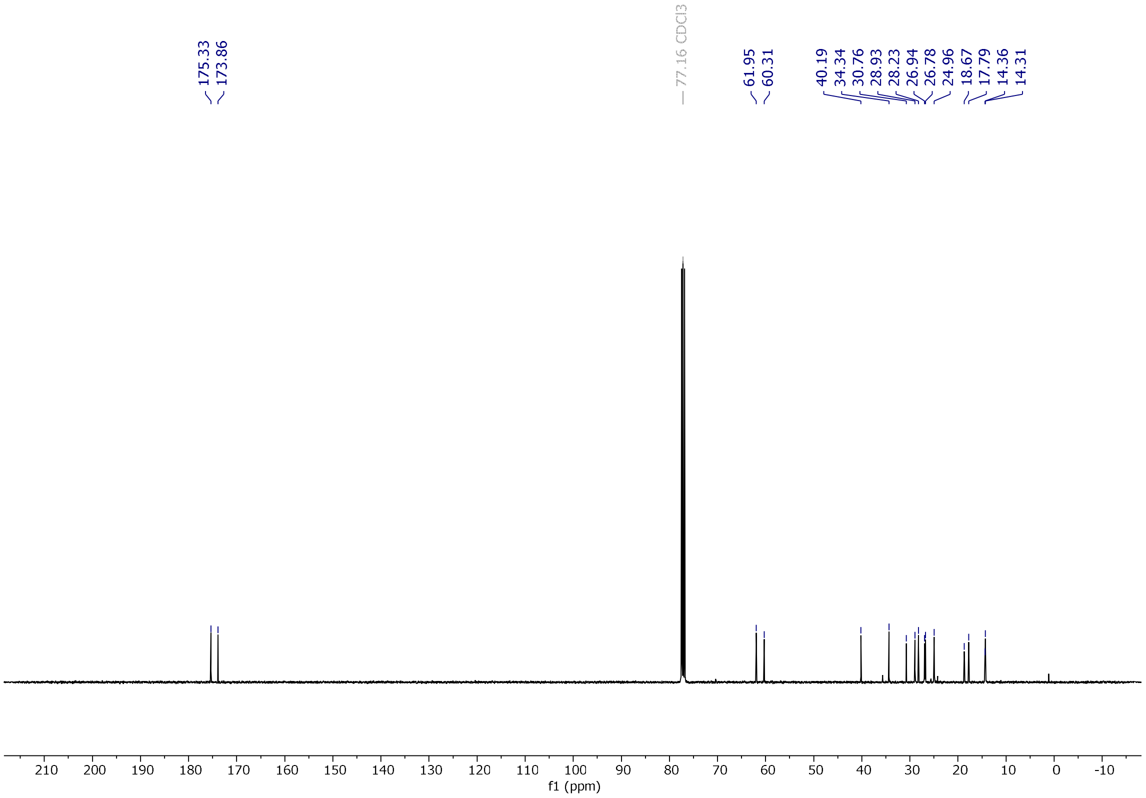

^{1}H NMR (300 MHz, $CDCl_3$) of **6**

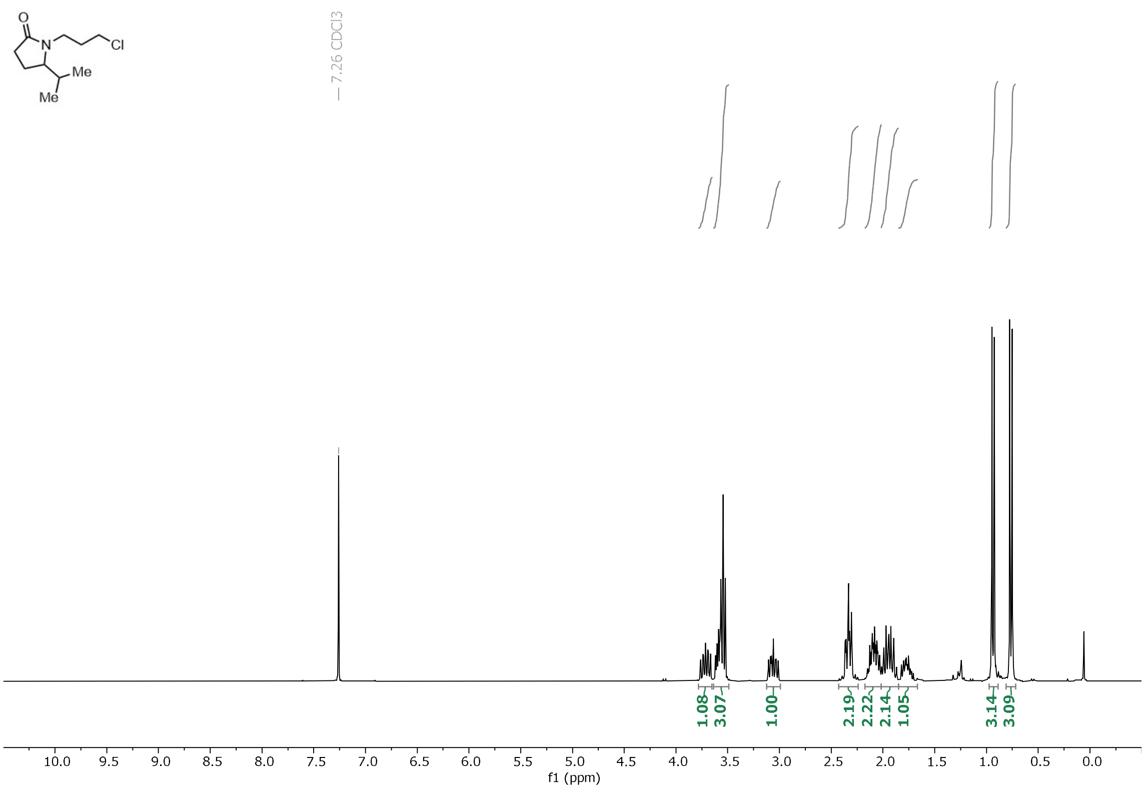

^{13}C NMR (101 MHz, $CDCl_3$) of **6**

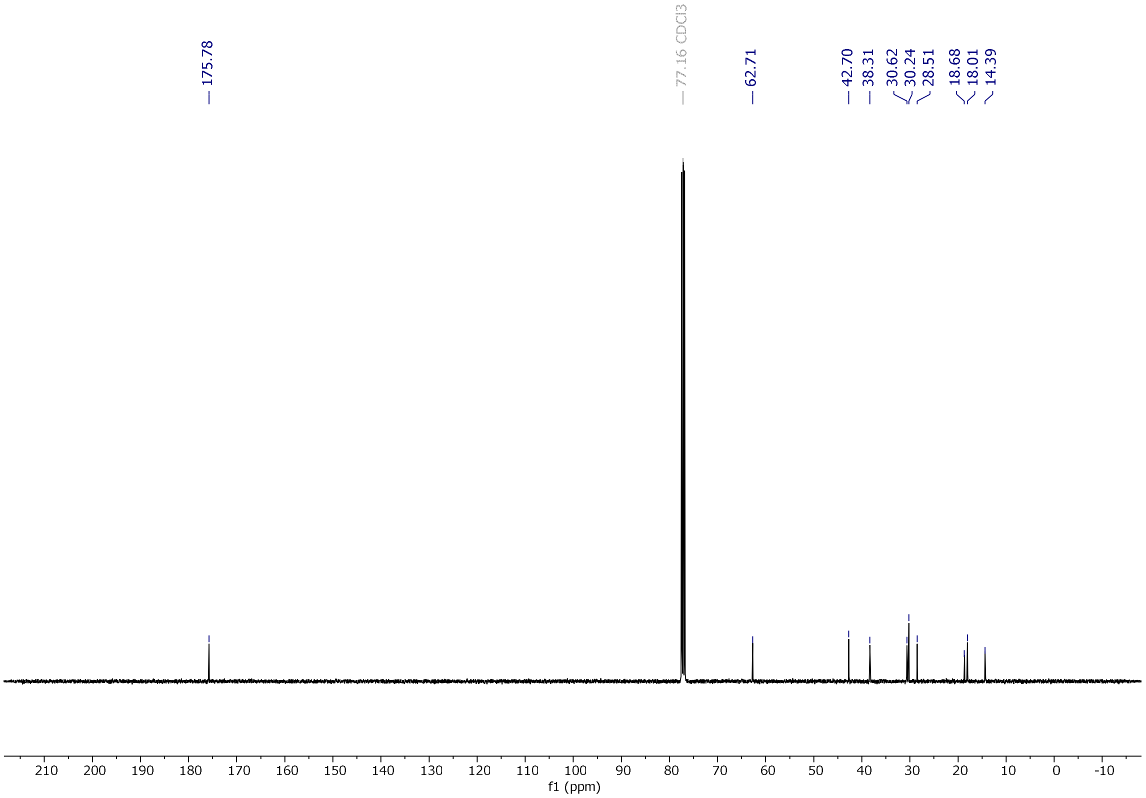

^{1}H NMR (400 MHz, $CDCl_3$) of **7**

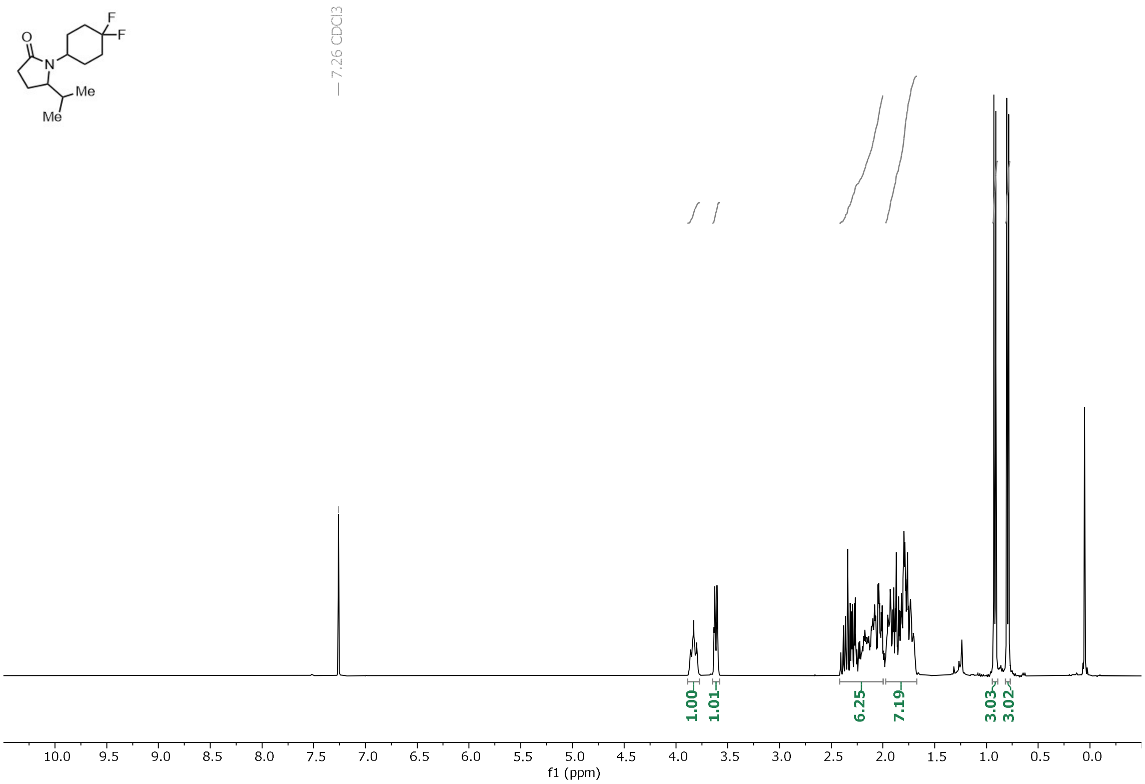

^{13}C NMR (101 MHz, $CDCl_3$) of **7**

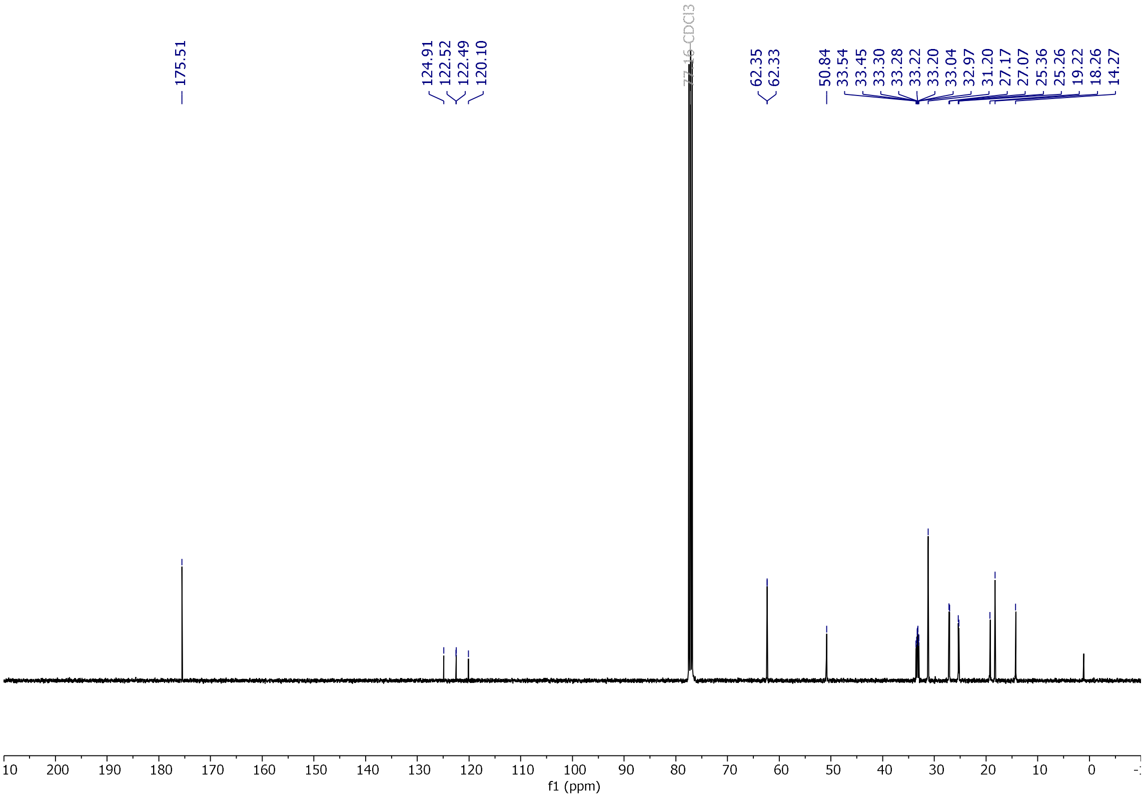

^{19}F NMR (282 MHz, $CDCl_3$) of **7**

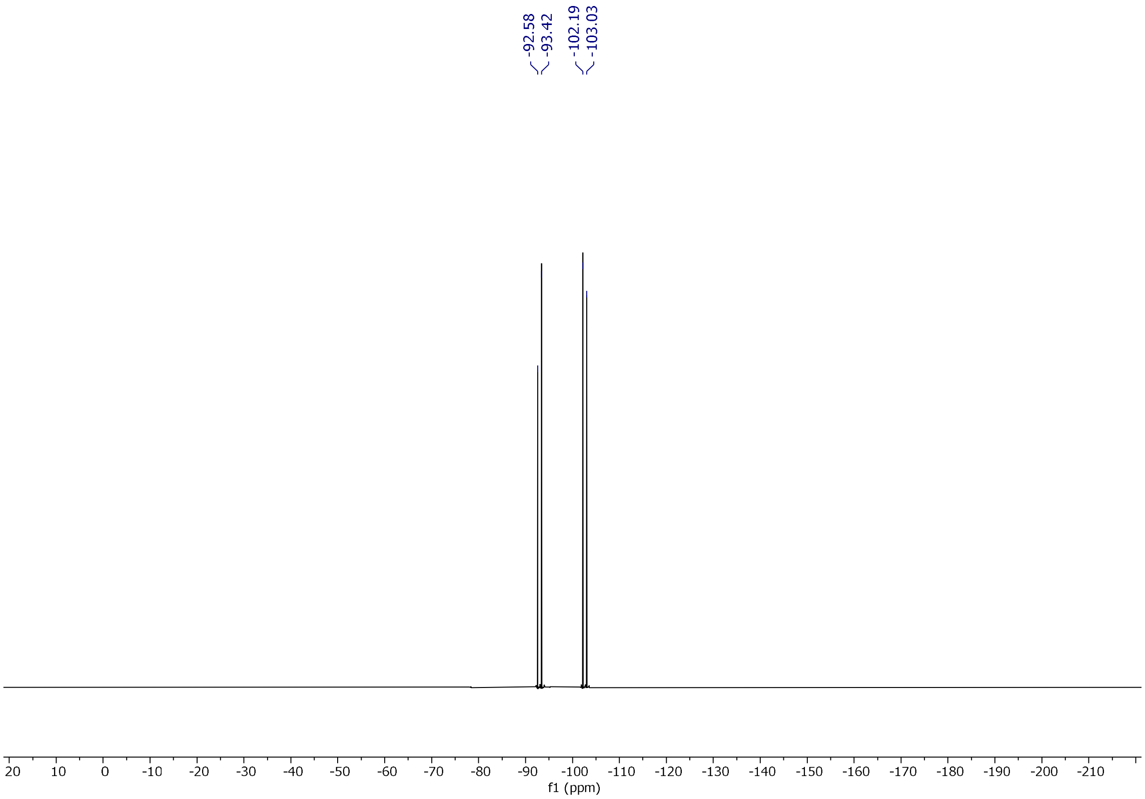

^{1}H NMR (400 MHz, $CDCl_3$) of **8**

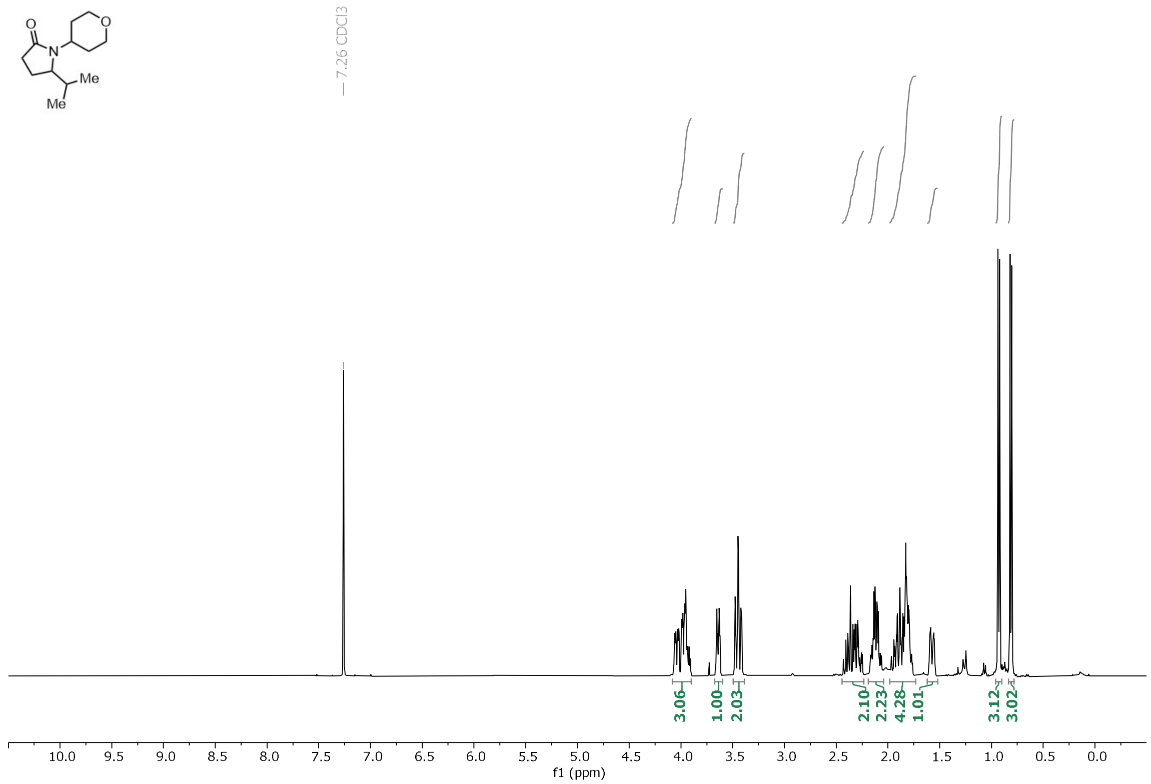

^{13}C NMR (101 MHz, $CDCl_3$) of **8**

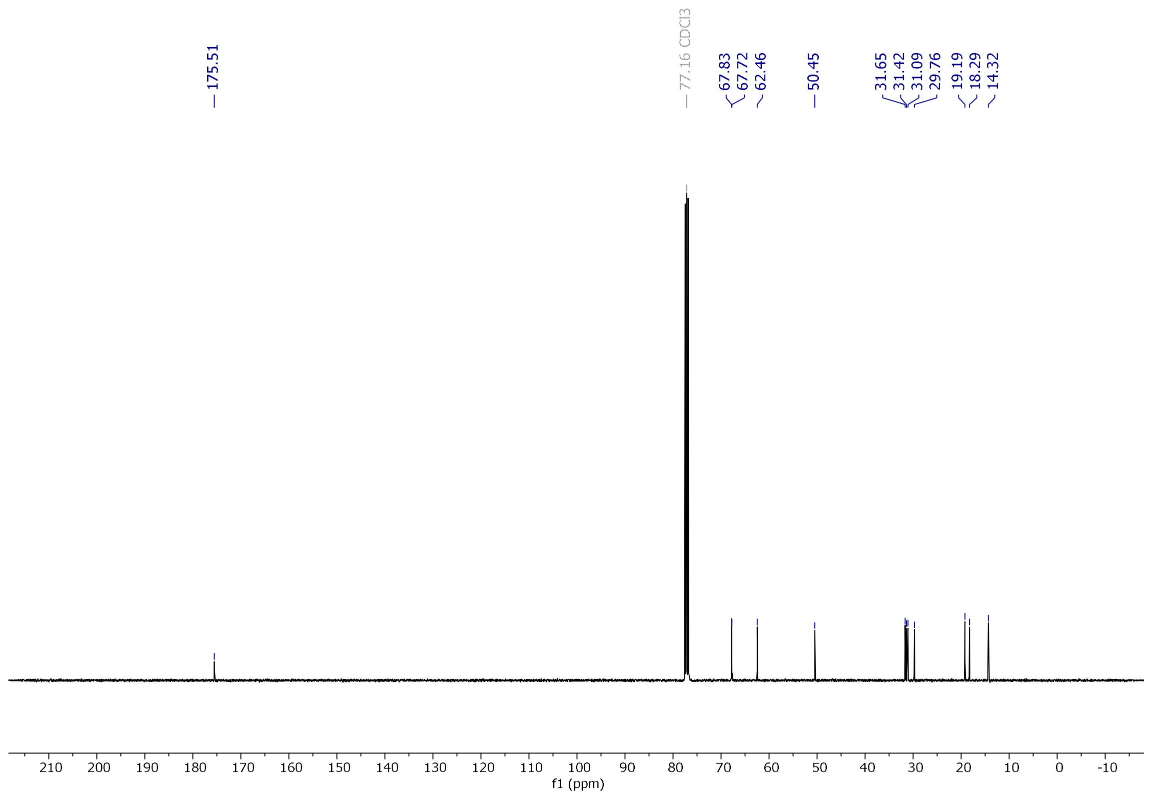

^{1}H NMR (400 MHz, $CDCl_3$) of **9**

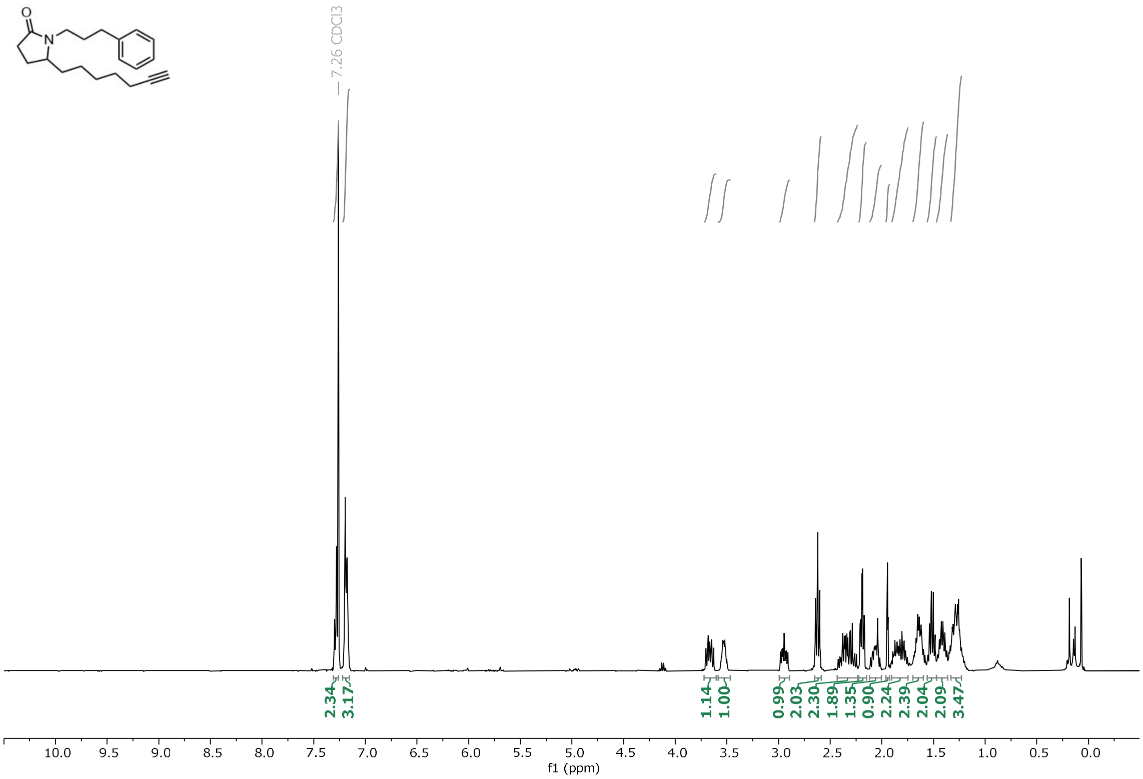

^{13}C NMR (101 MHz, $CDCl_3$) of **9**

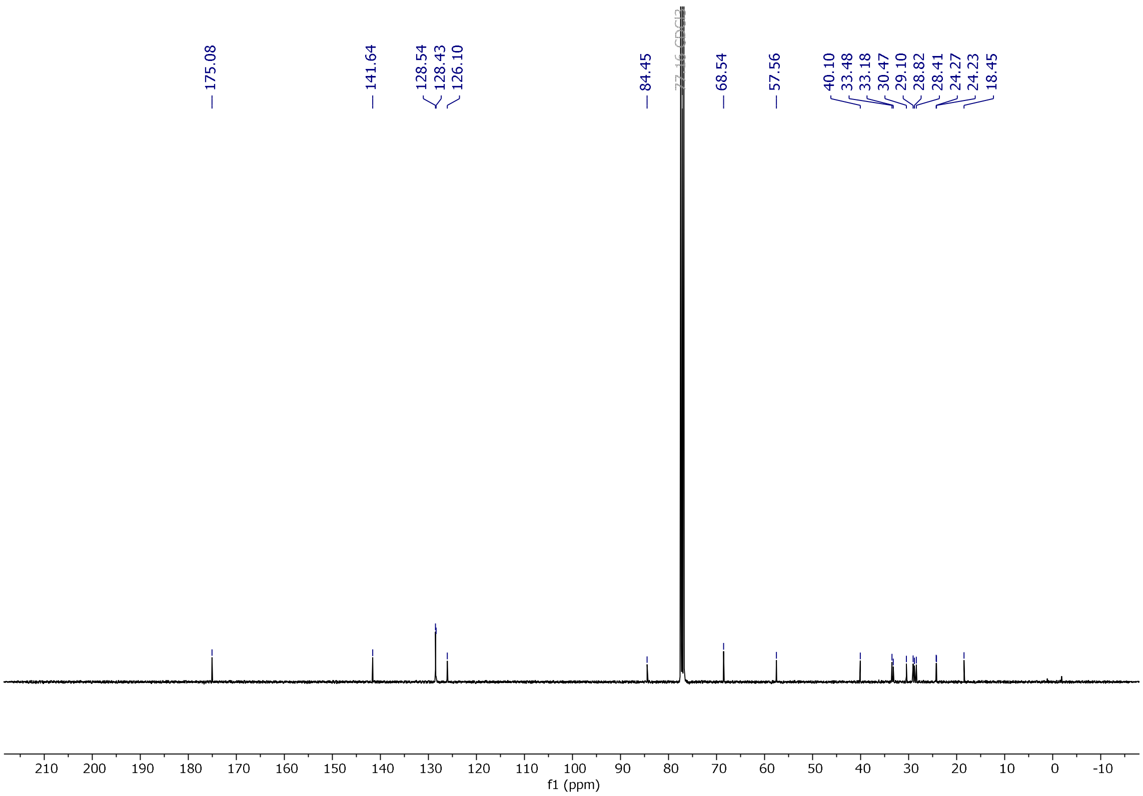

^{1}H NMR (400 MHz, $CDCl_3$) of **10**

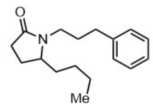

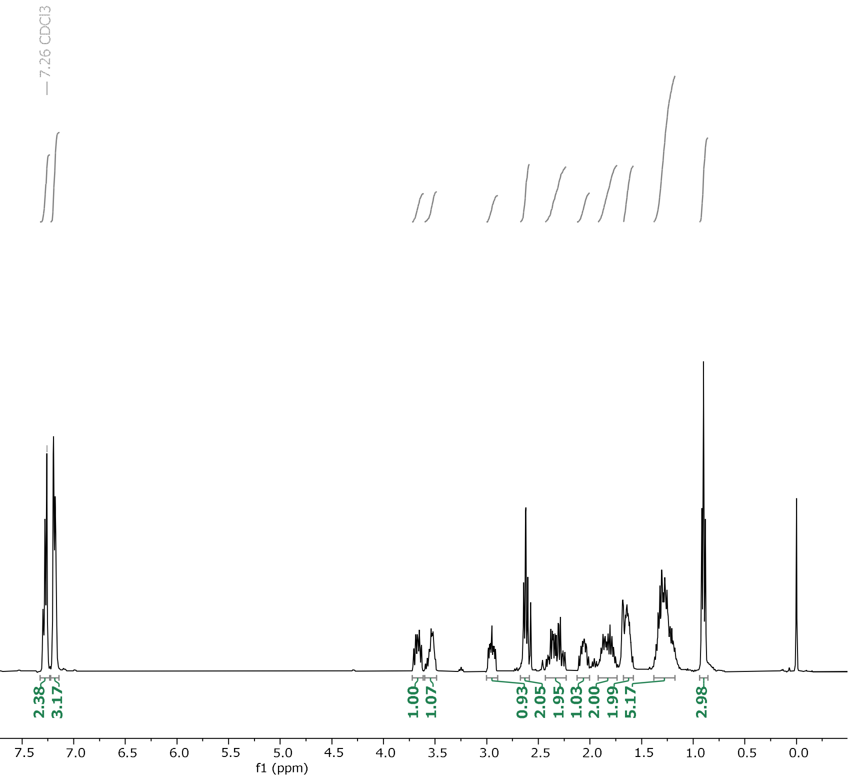

^{13}C NMR (101 MHz, $CDCl_3$) of **10**

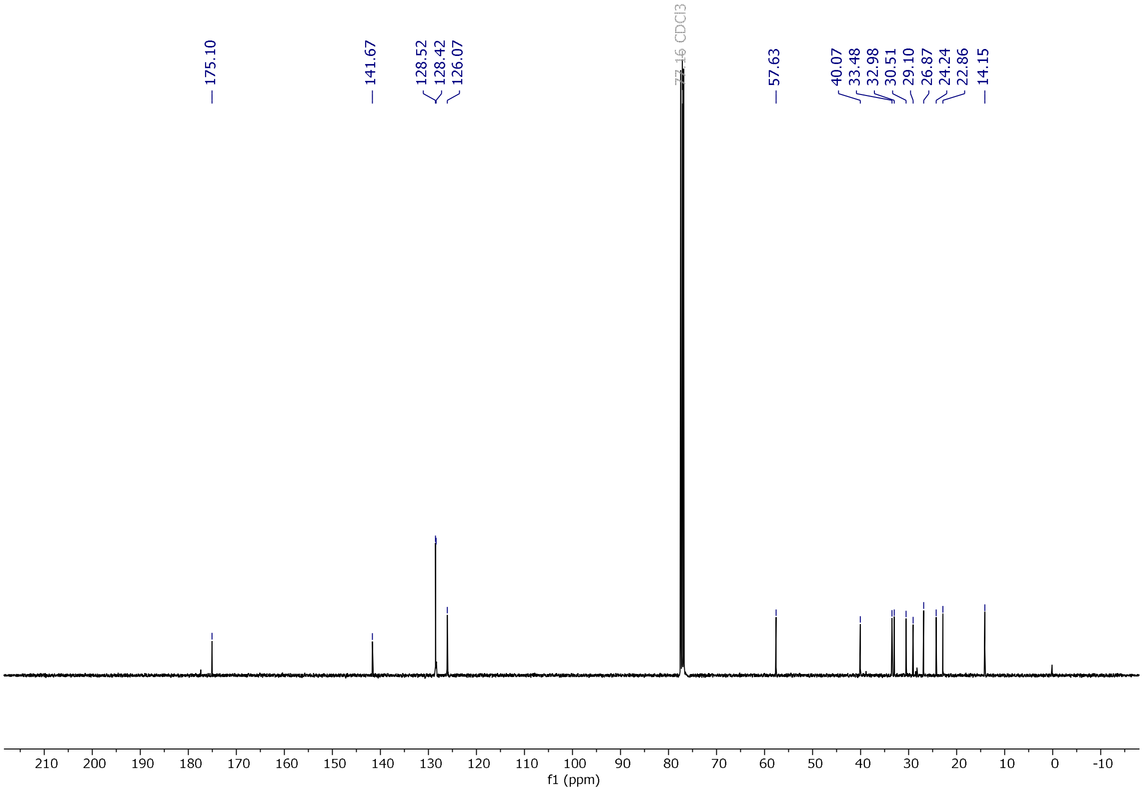

^{1}H NMR (400 MHz, $CDCl_3$) of **11**

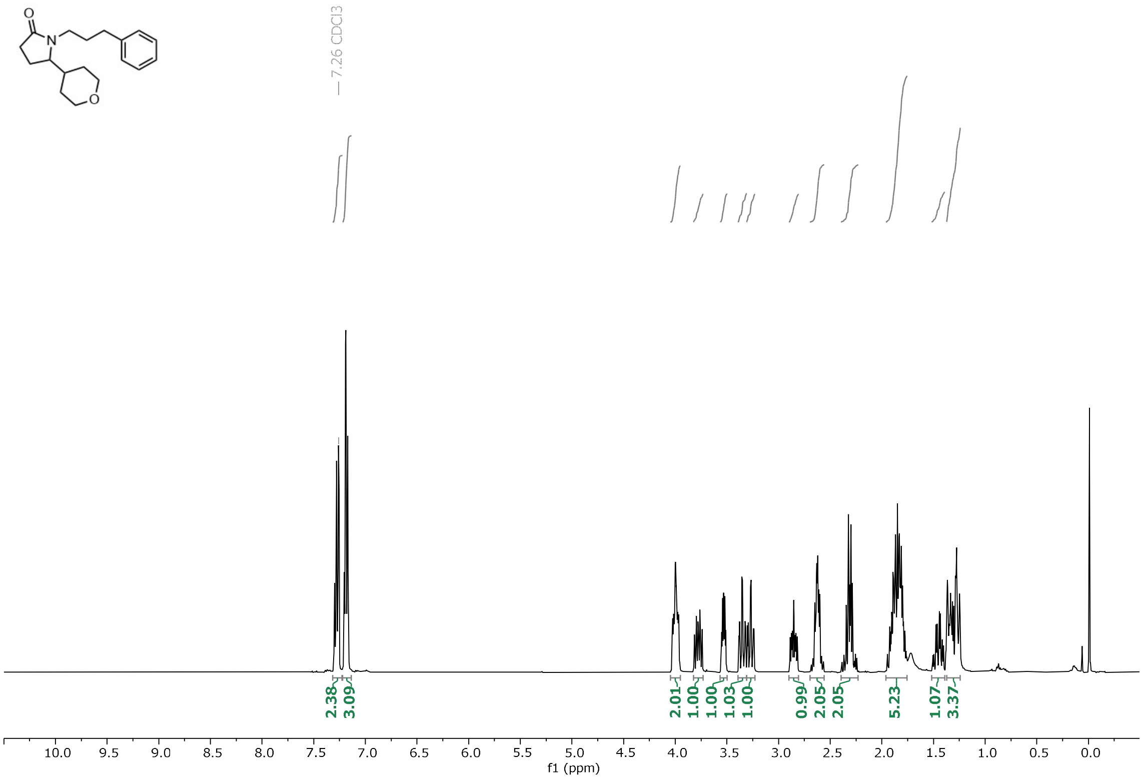

^{13}C NMR (101 MHz, $CDCl_3$) of **11**

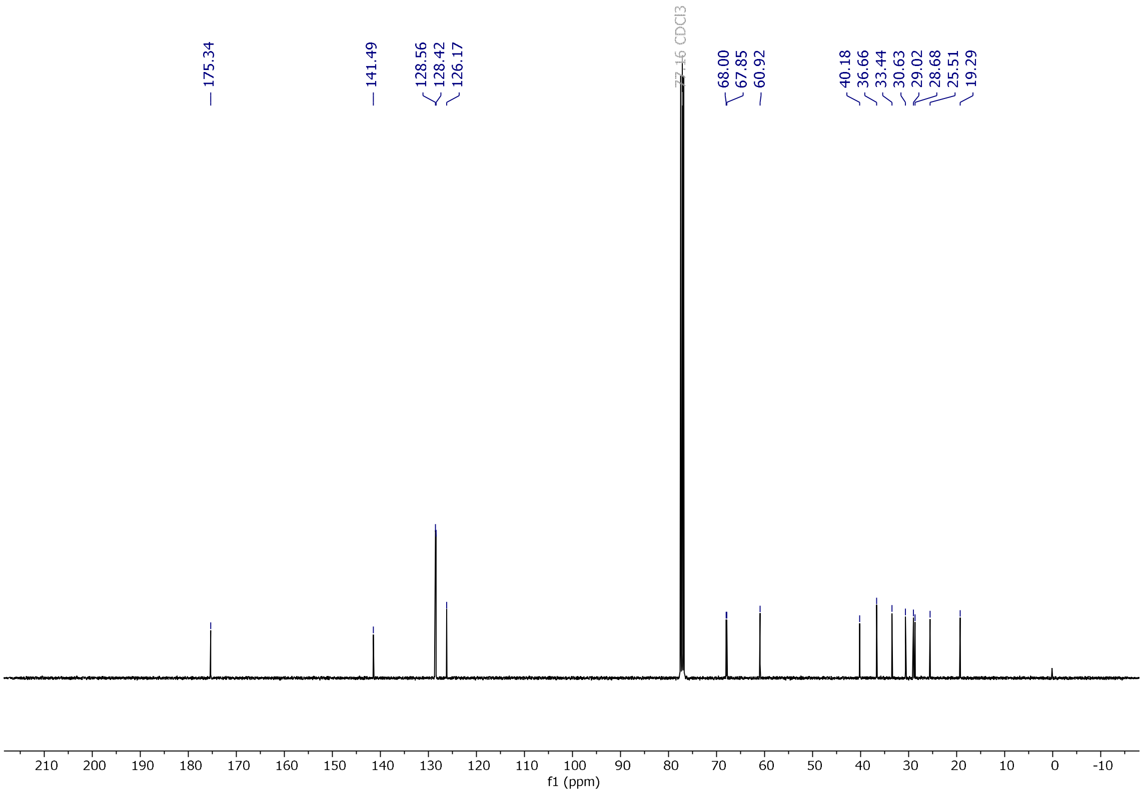

^{1}H NMR (300 MHz, $CDCl_3$) of **12**

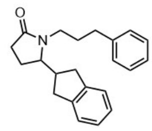

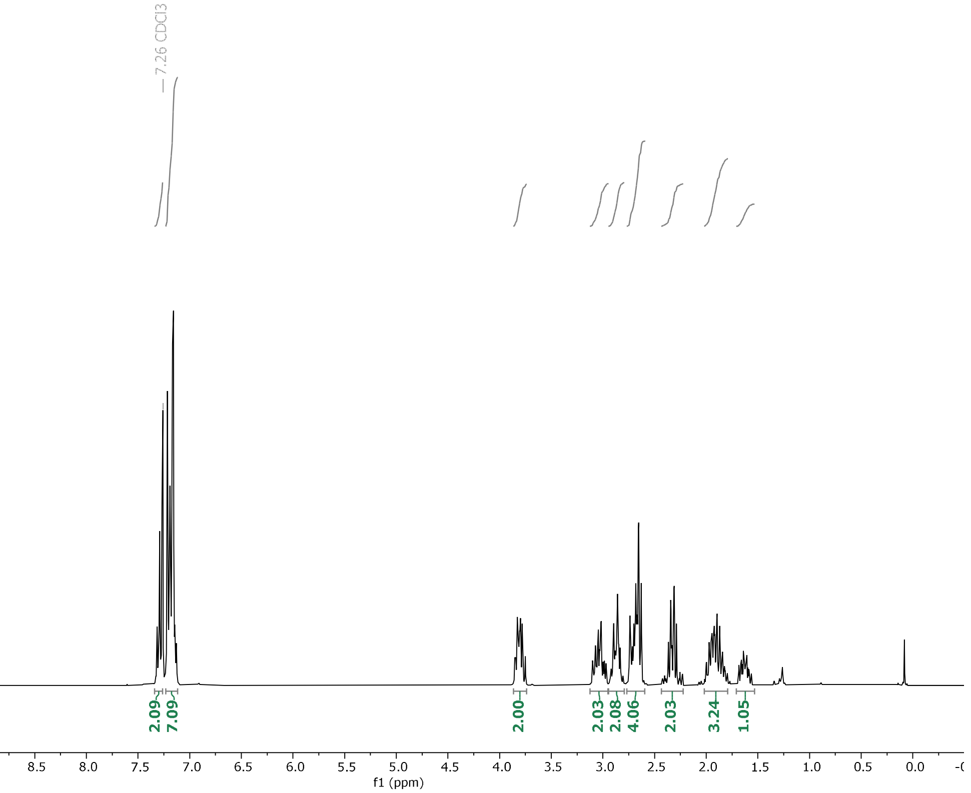

^{13}C NMR (101 MHz, $CDCl_3$) of **12**

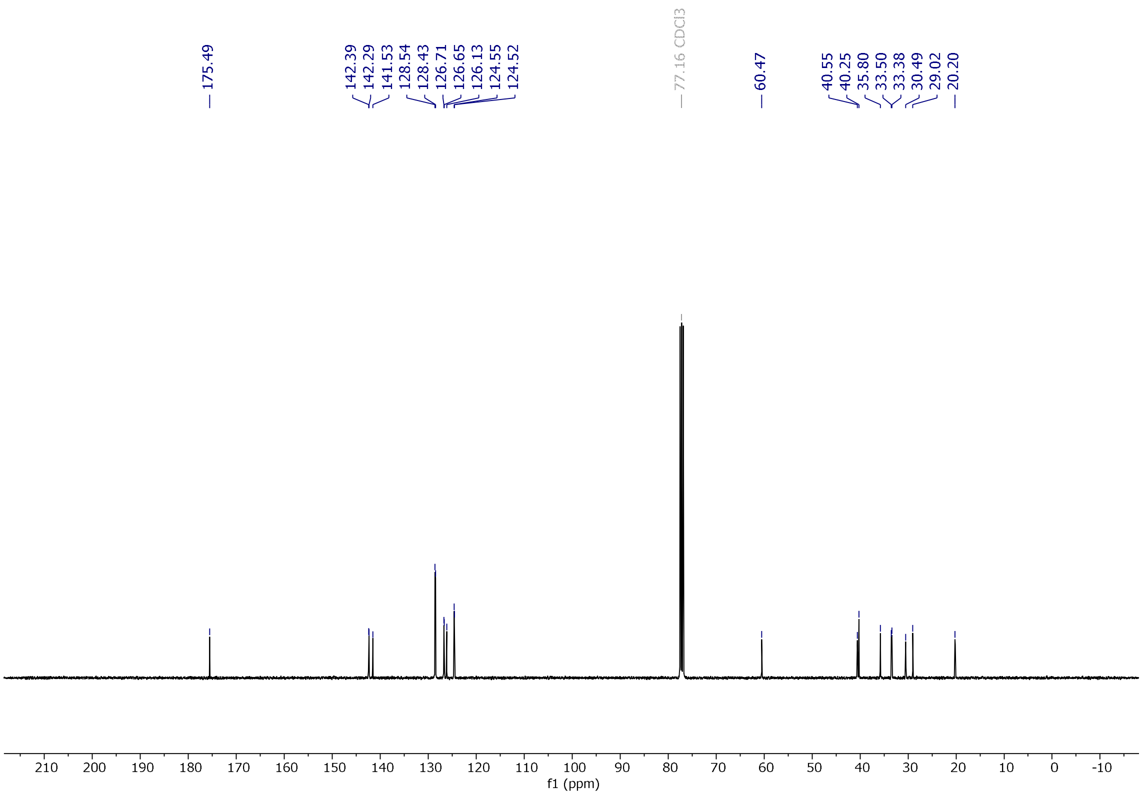

^{1}H NMR (300 MHz, $CDCl_3$) of **13**

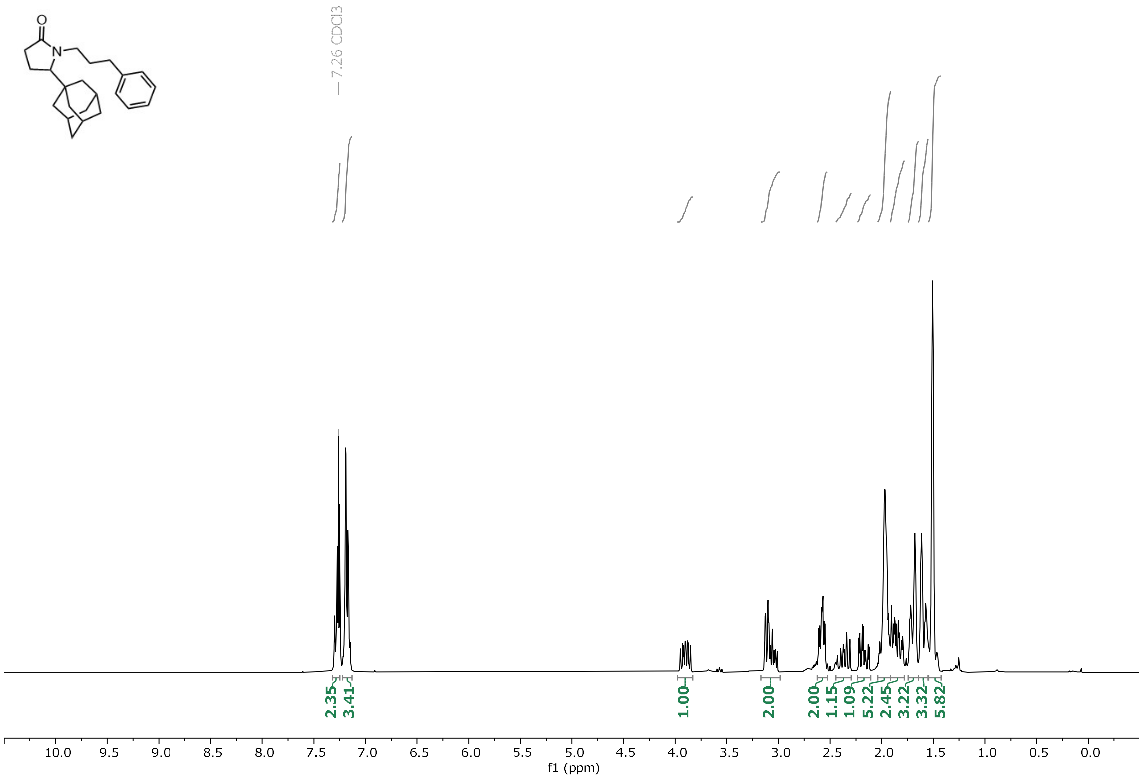

^{13}C NMR (101 MHz, $CDCl_3$) of **13**

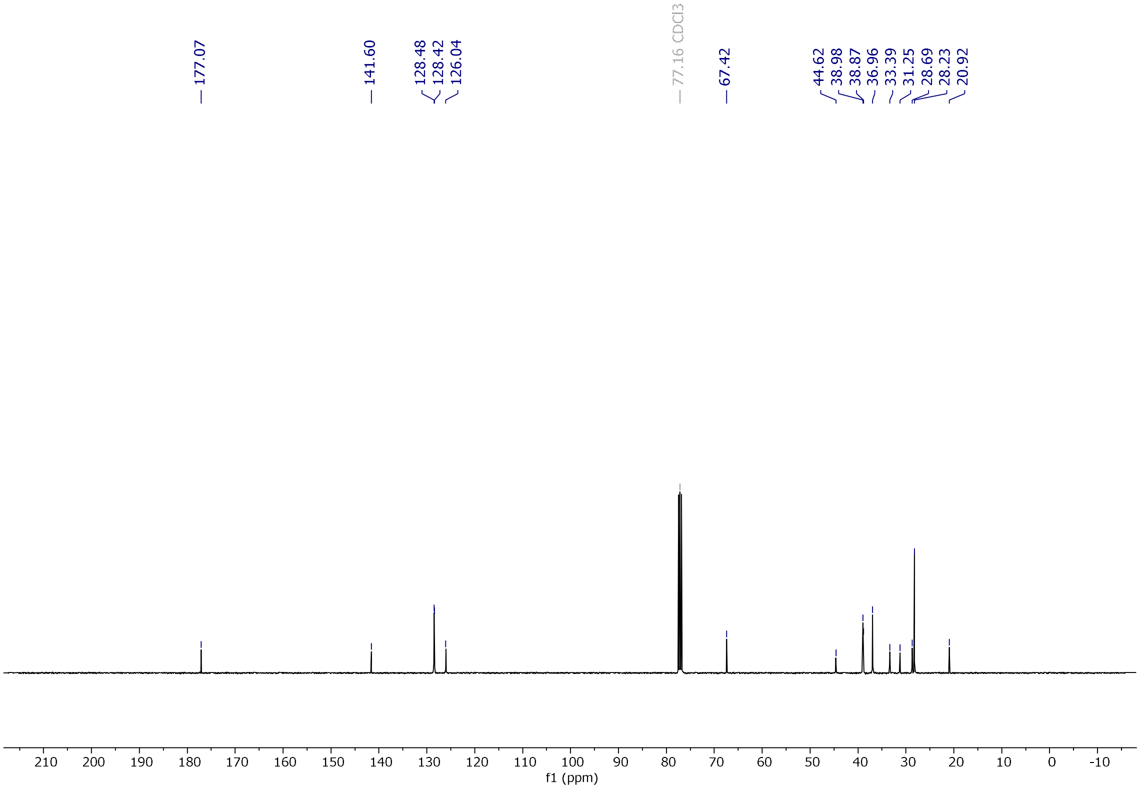

^{1}H NMR (400 MHz, $CDCl_3$) of **3**

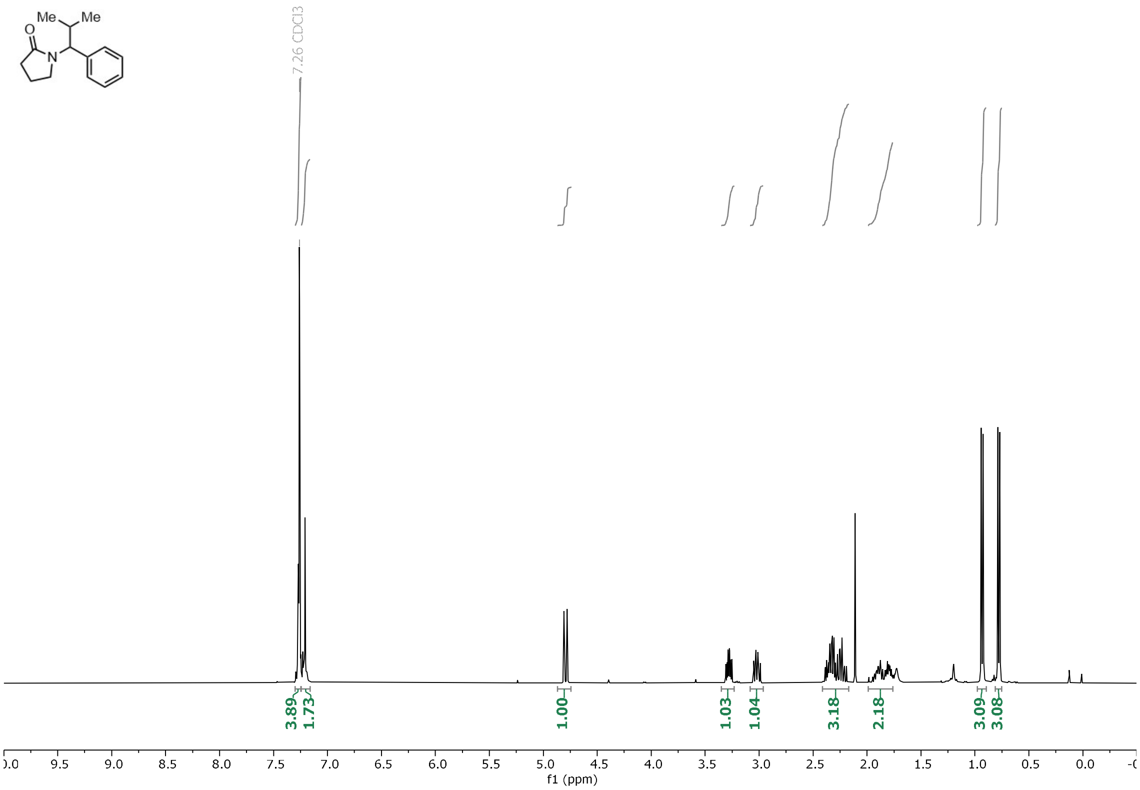

^{13}C NMR (101 MHz, $CDCl_3$) of **3**

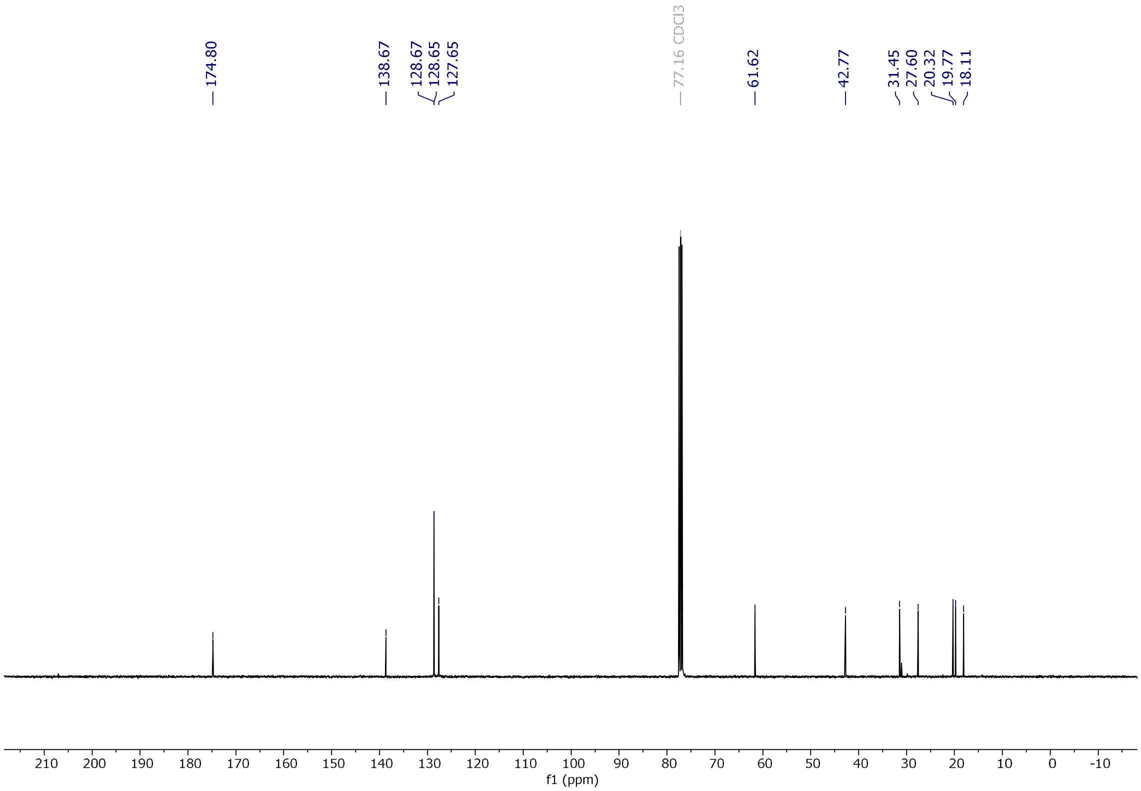

^{1}H NMR (400 MHz, $CDCl_3$) of **14**

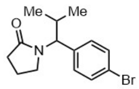

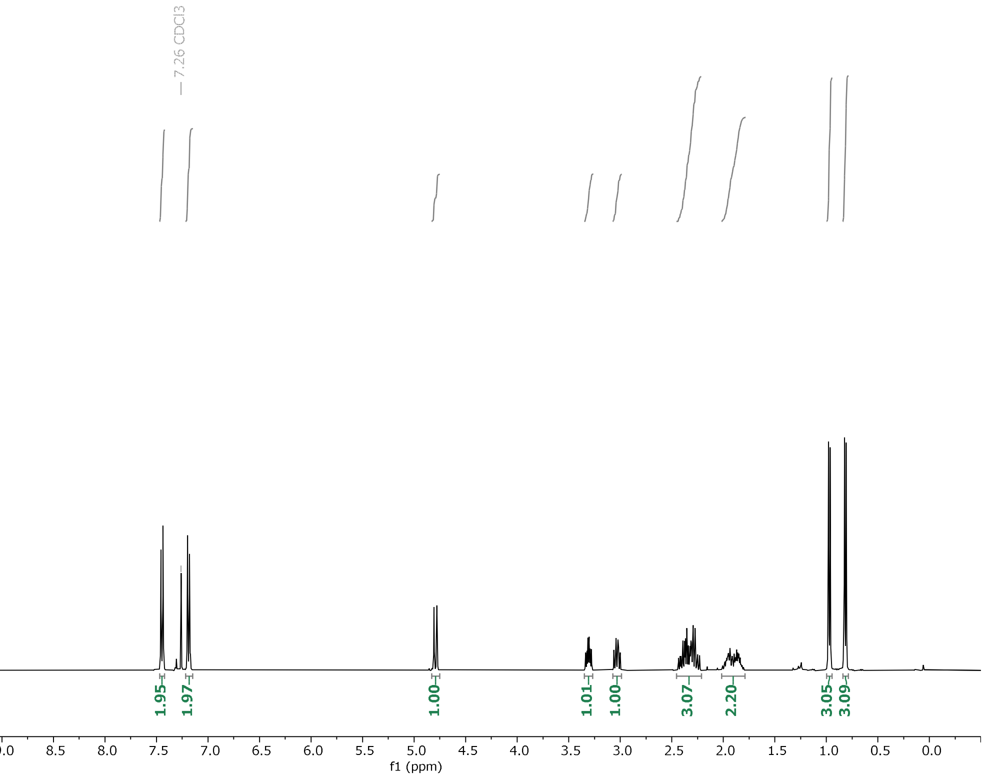

^{13}C NMR (101 MHz, $CDCl_3$) of **14**

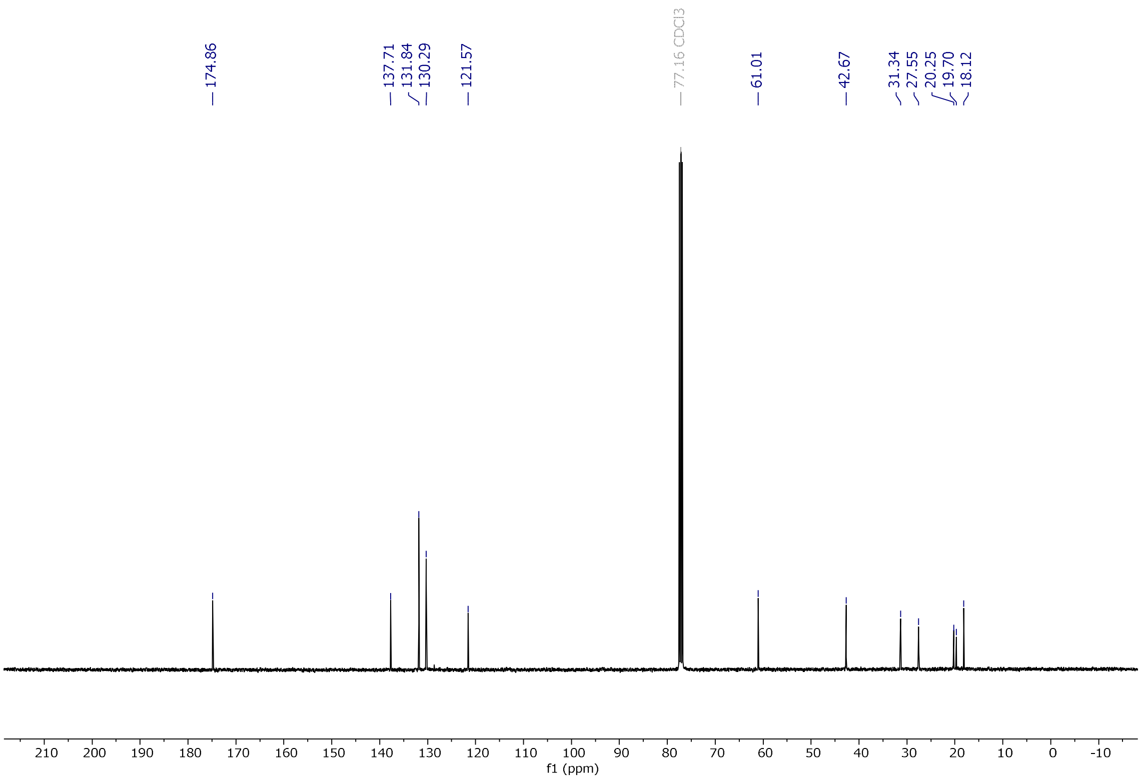

^{1}H NMR (300 MHz, $CDCl_3$) of **15**

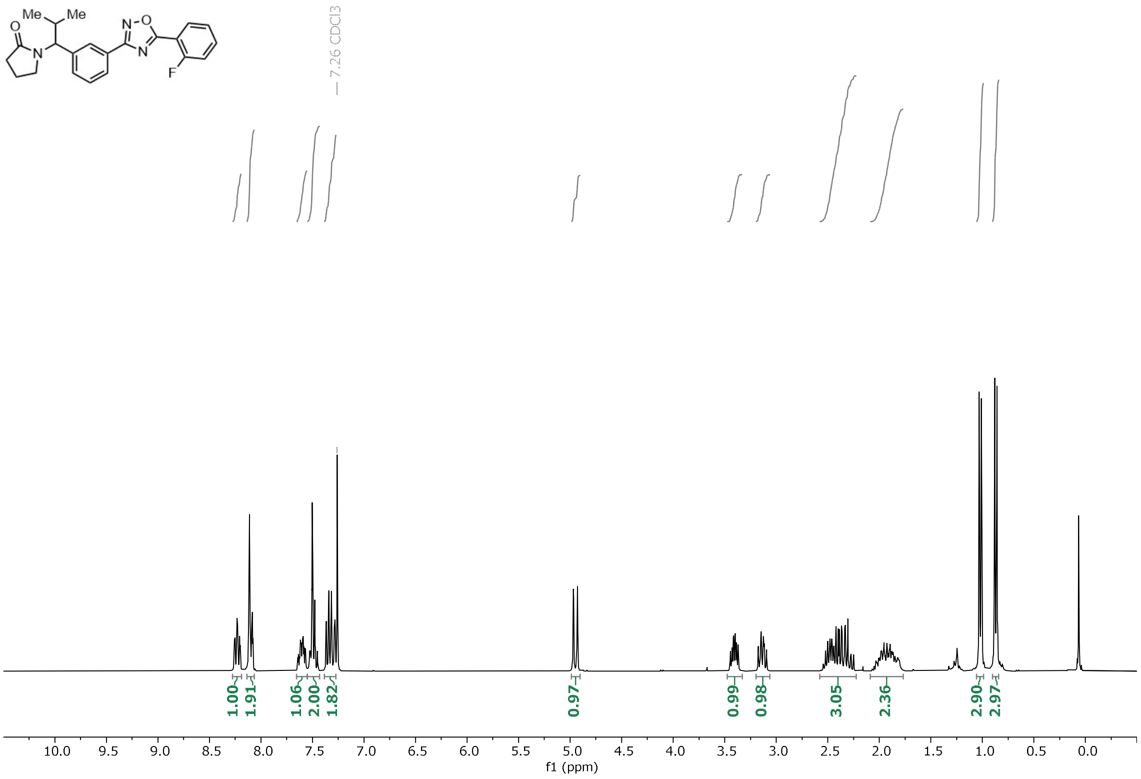

^{13}C NMR (101 MHz, $CDCl_3$) of **15**

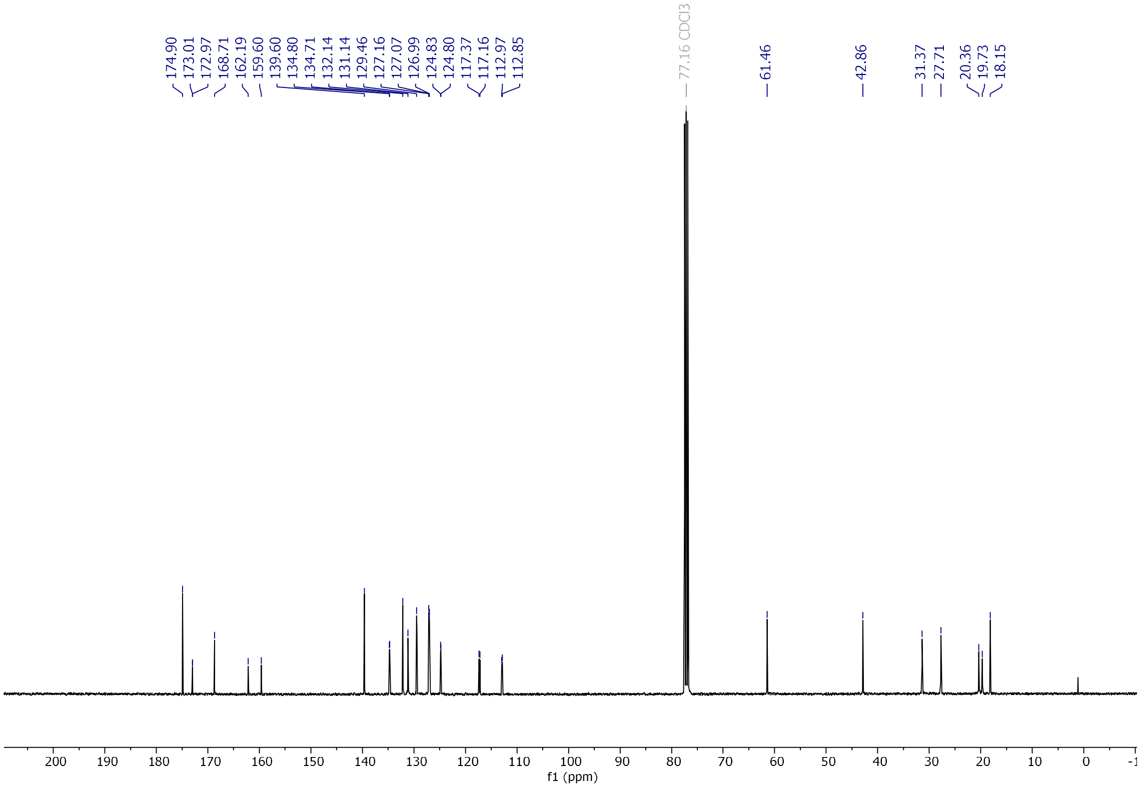

^{19}F NMR (282 MHz, $CDCl_3$) of **15**

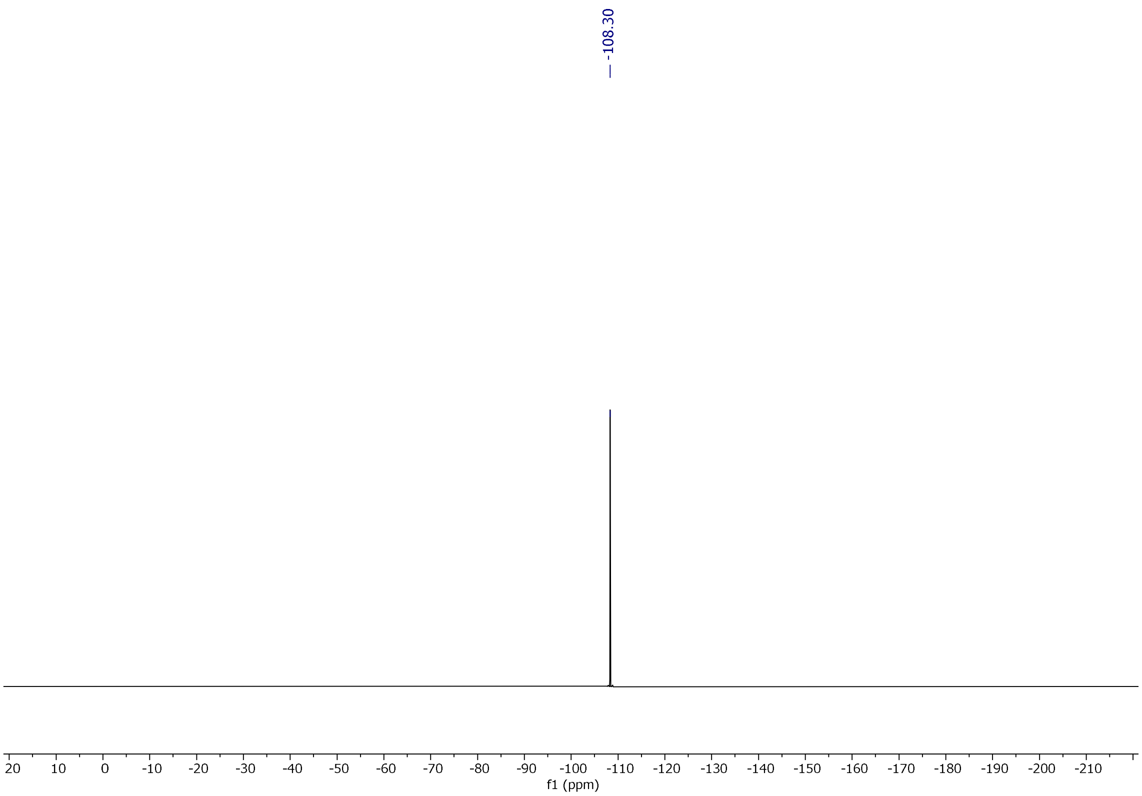
-108.30
20
10
0
-10
-20
-30
-40
-50
-60
-70
-80
-90
-100
-110
-120
-130
-140
-150
-160
-170
-180
-190
-200
-210
f1 (ppm)

^{1}H NMR (300 MHz, $CDCl_3$) of **16**

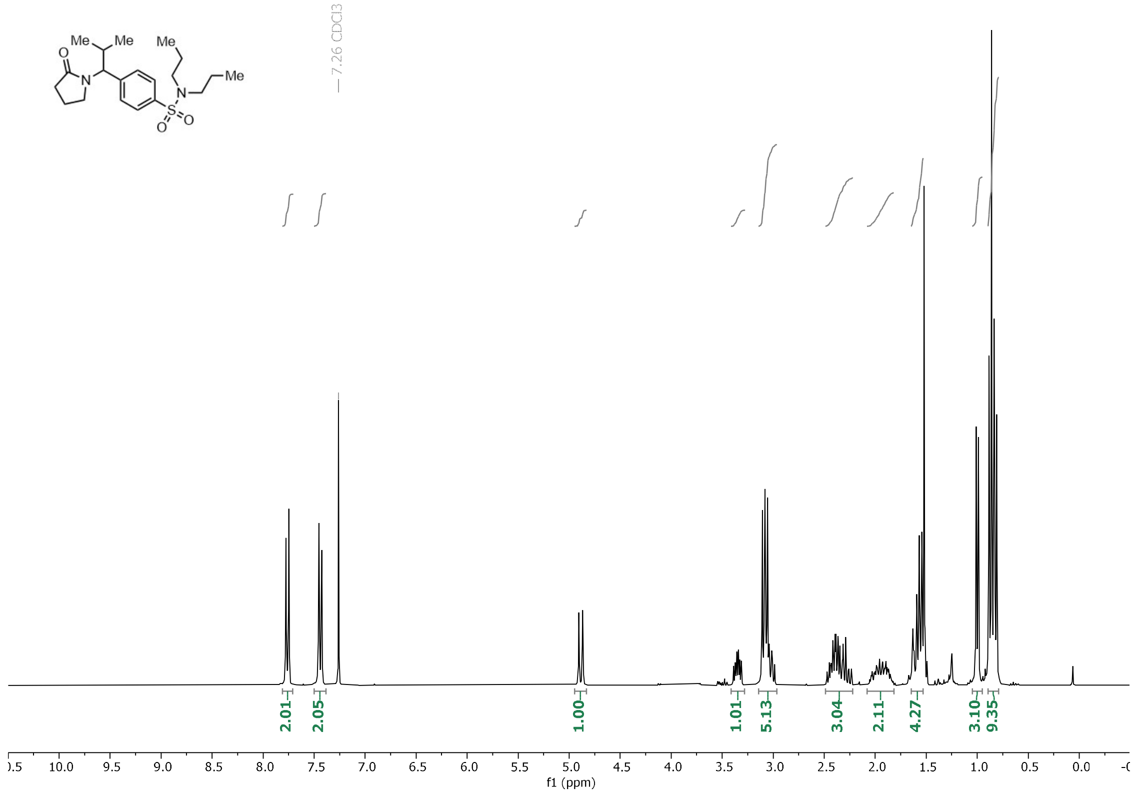

^{13}C NMR (101 MHz, $CDCl_3$) of **16**

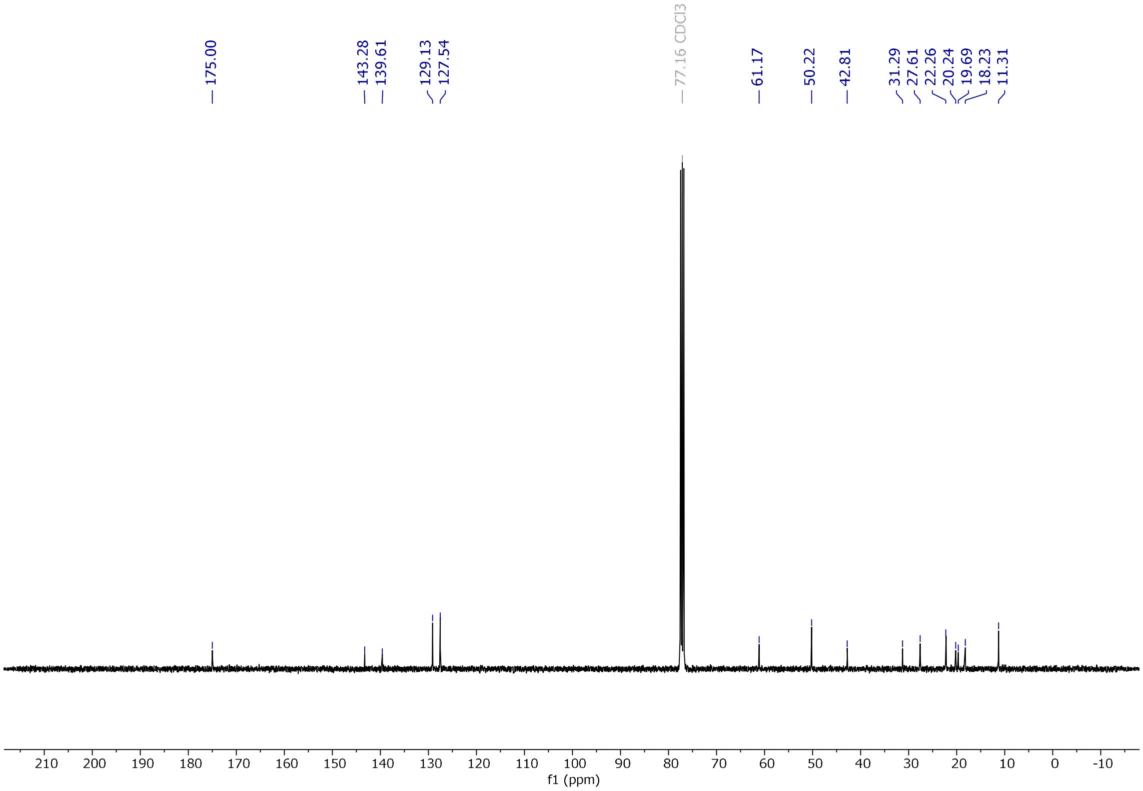

^{1}H NMR (300 MHz, $CDCl_3$) of **17**

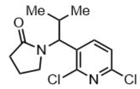

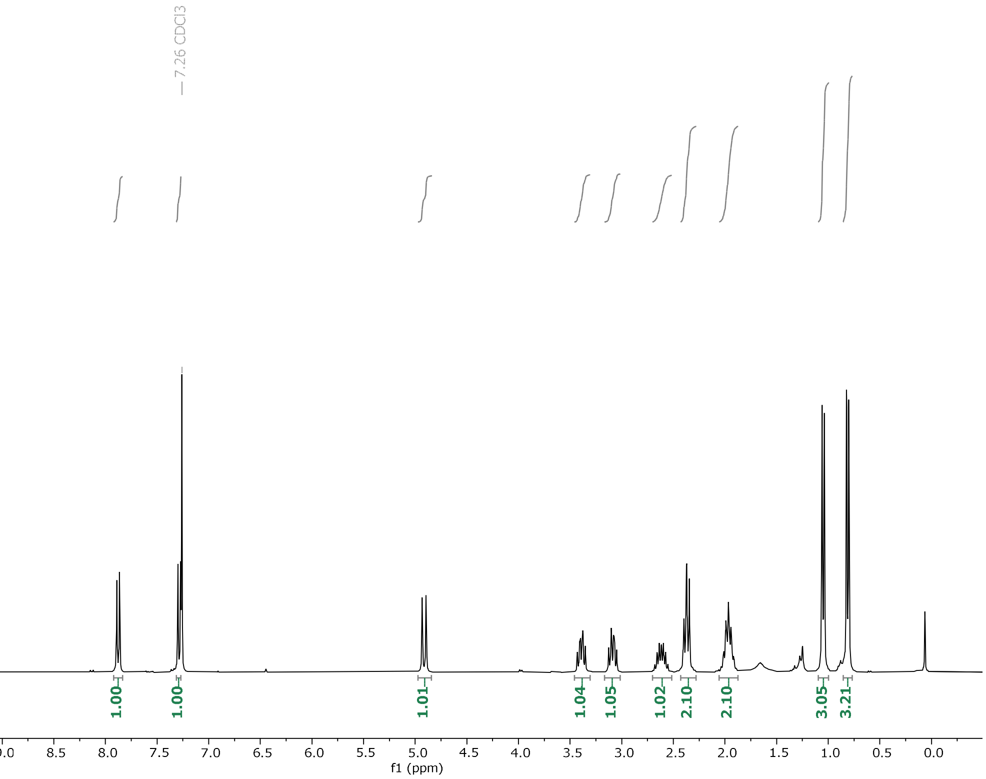

^{13}C NMR (101 MHz, $CDCl_3$) of **17**

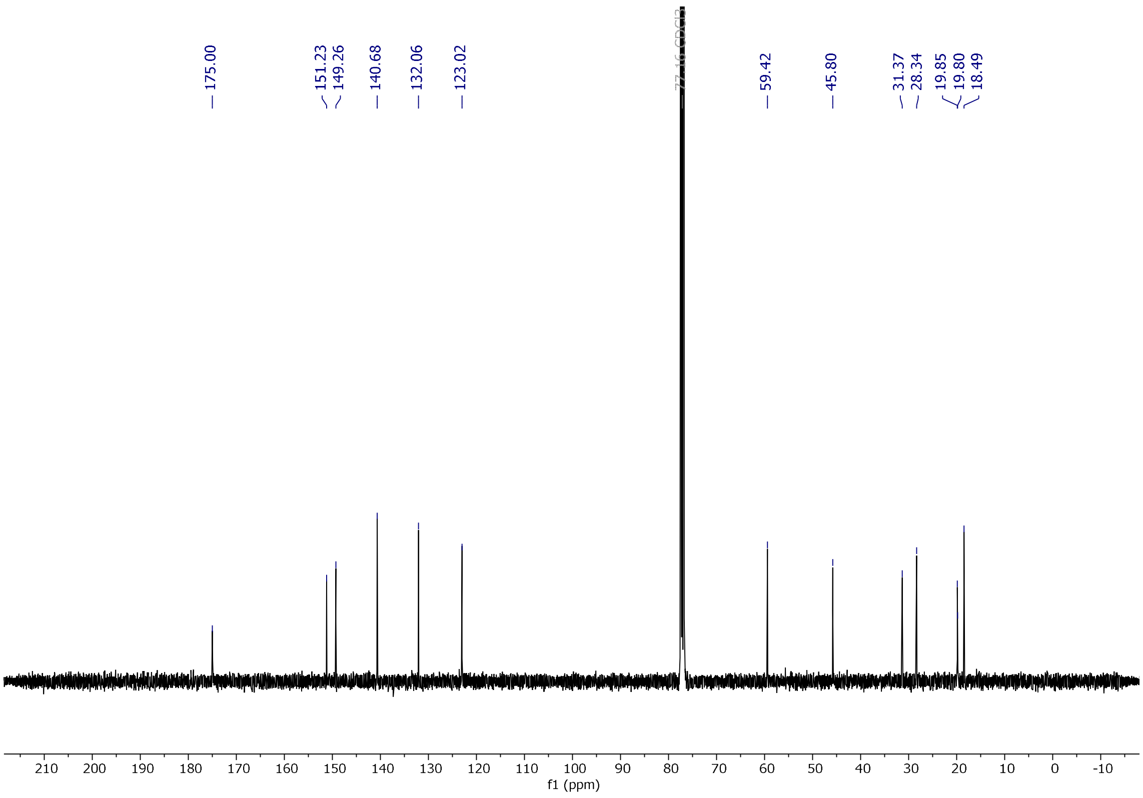

^{1}H NMR (400 MHz, $CDCl_3$) of **18**

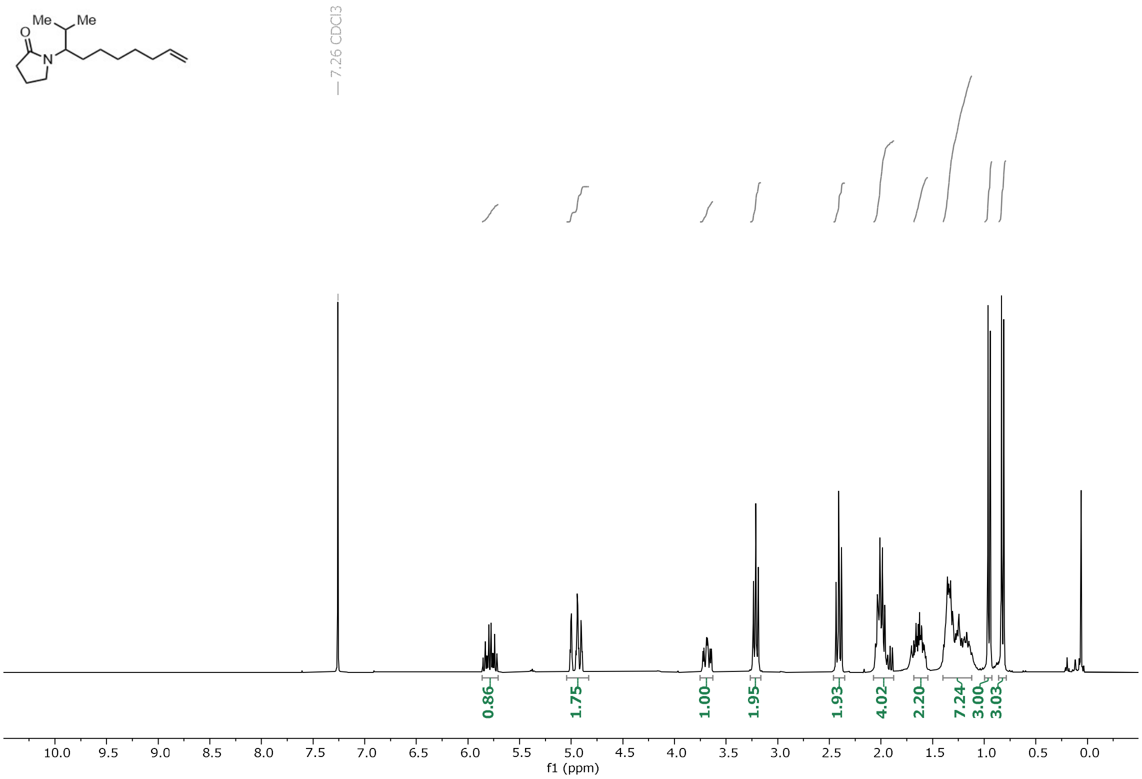

^{13}C NMR (101 MHz, $CDCl_3$) of **18**

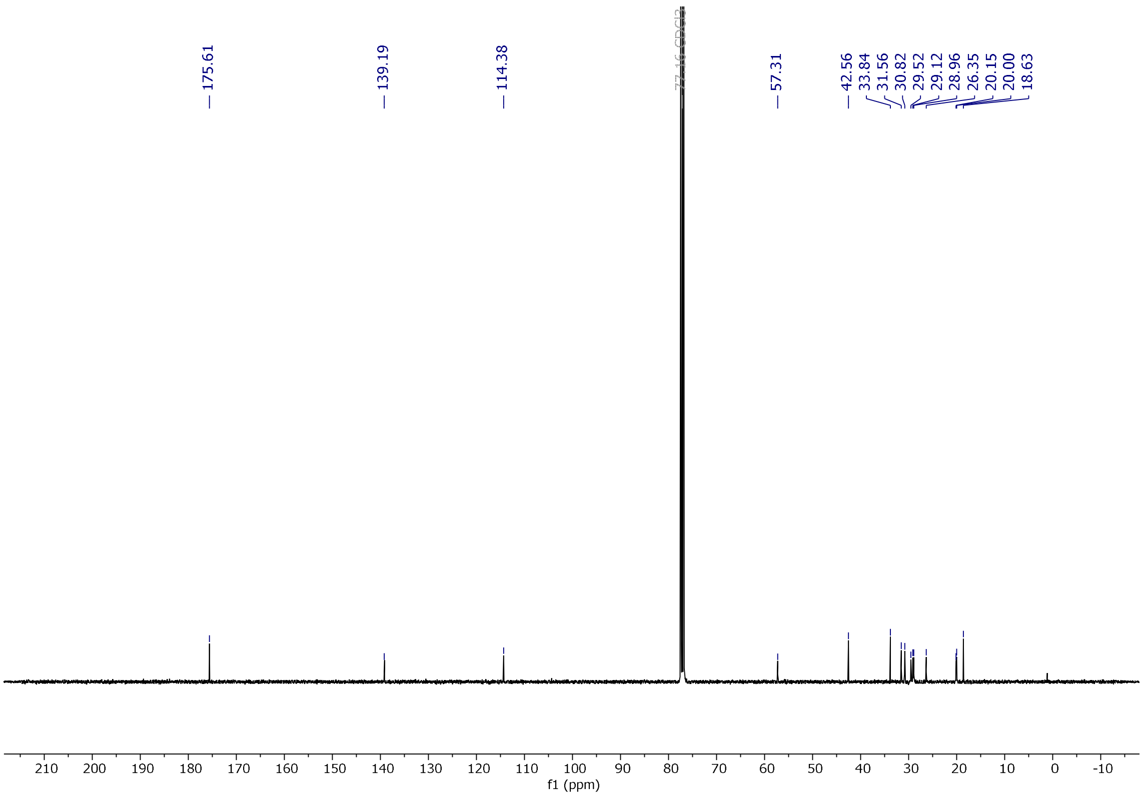

^{1}H NMR (400 MHz, $CDCl_3$) of **19**

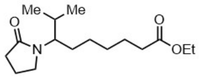

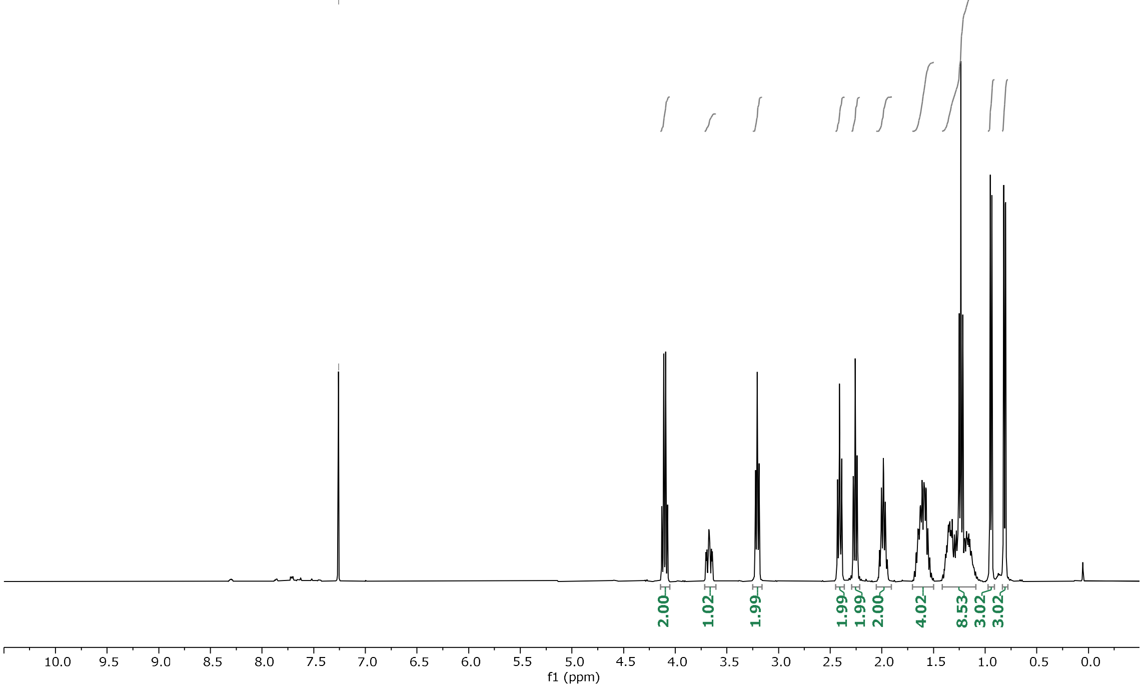

^{13}C NMR (101 MHz, $CDCl_3$) of **19**

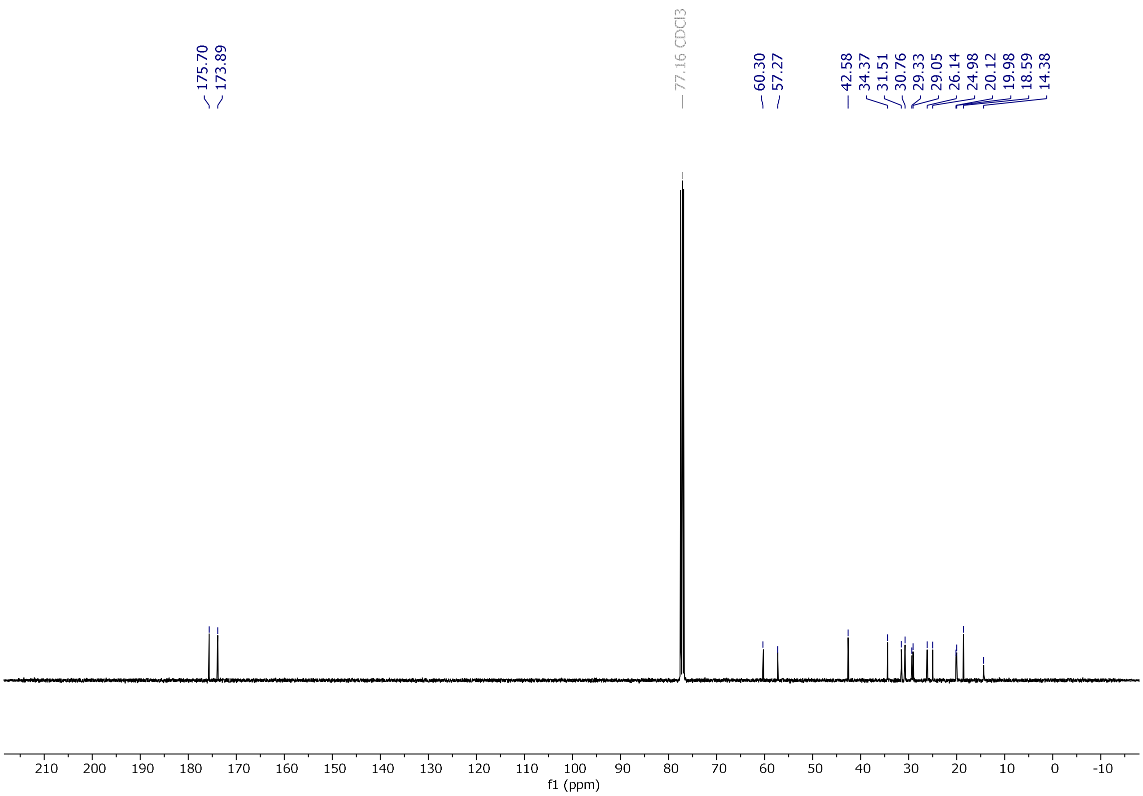

^{1}H NMR (400 MHz, $CDCl_3$) of **20**

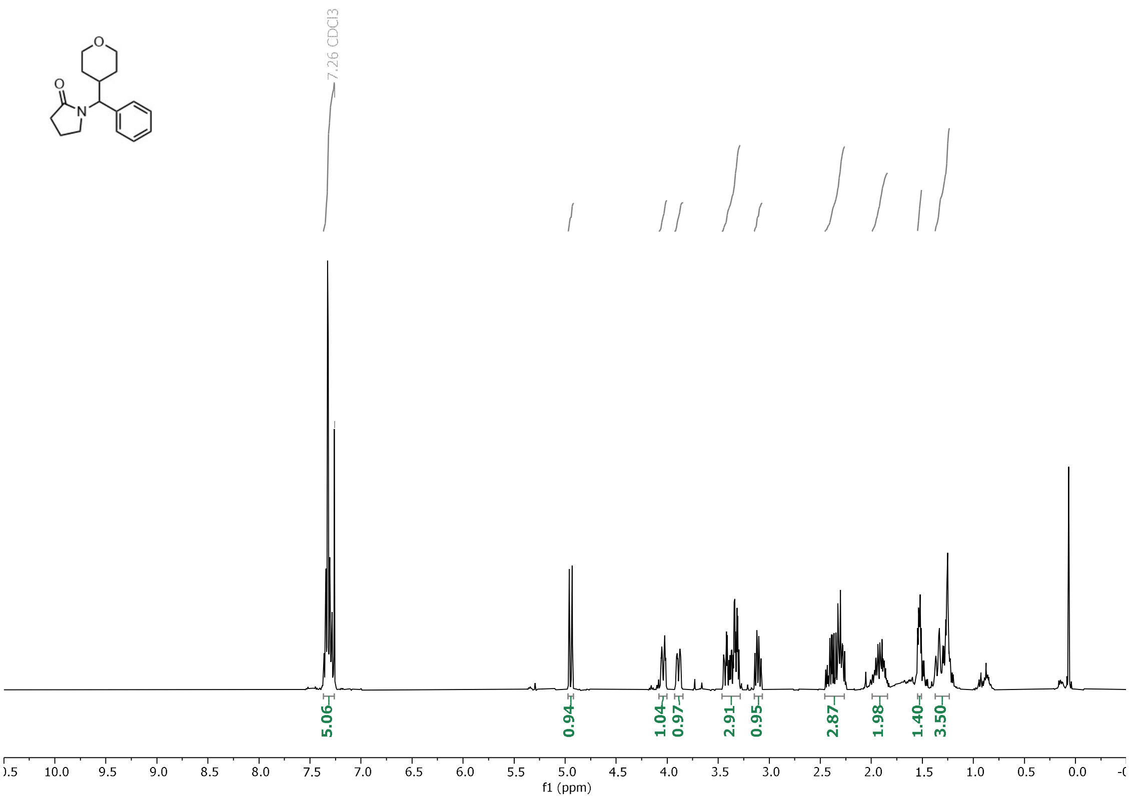

^{13}C NMR (101 MHz, $CDCl_3$) of **20**

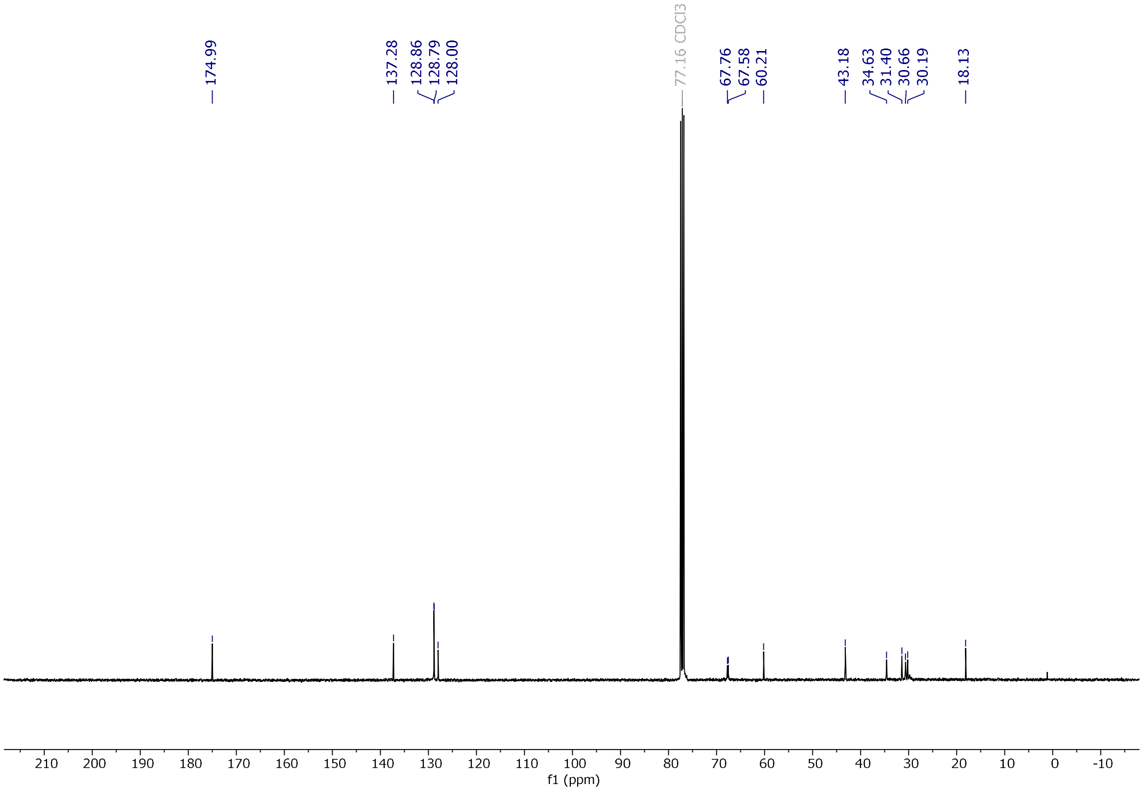

^{1}H NMR (400 MHz, $CDCl_3$) of **21**

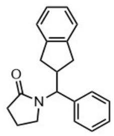

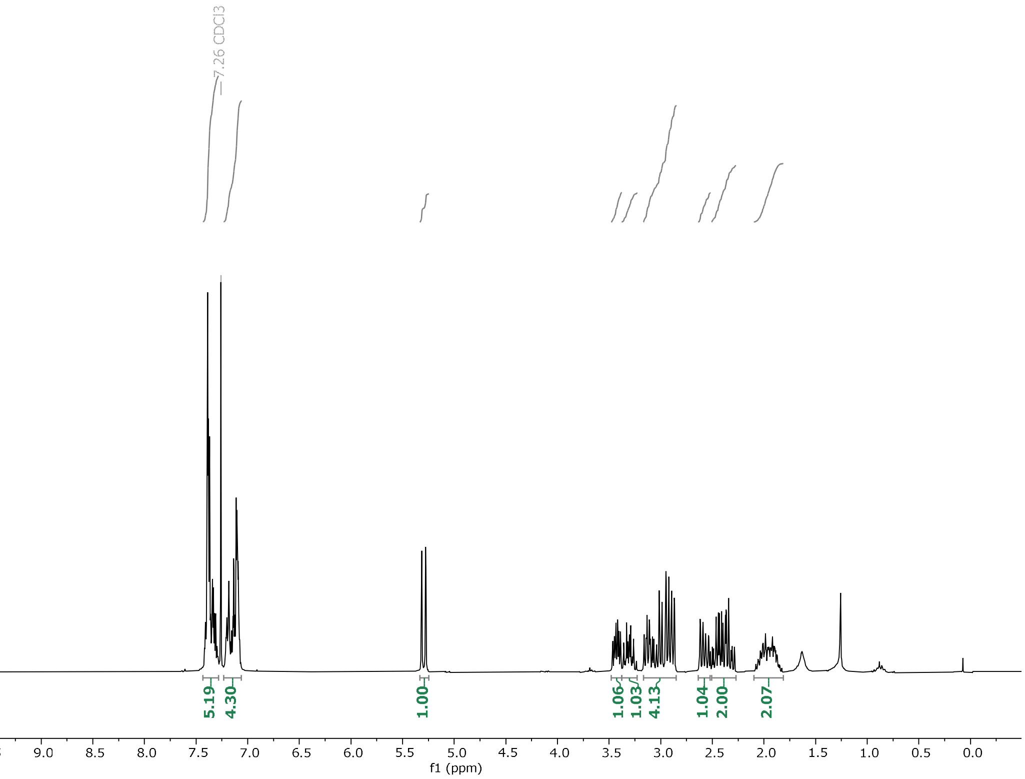

^{13}C NMR (101 MHz, $CDCl_3$) of **21**

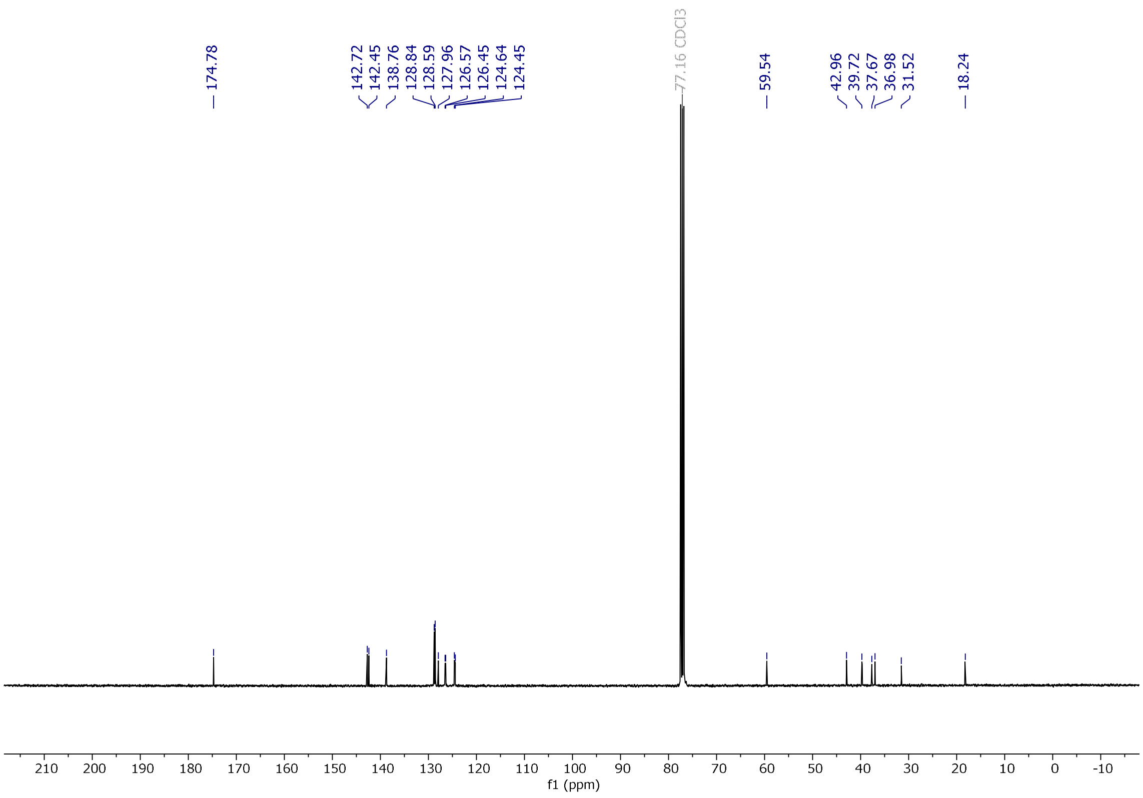

^{1}H NMR (400 MHz, $CDCl_3$) of **22**

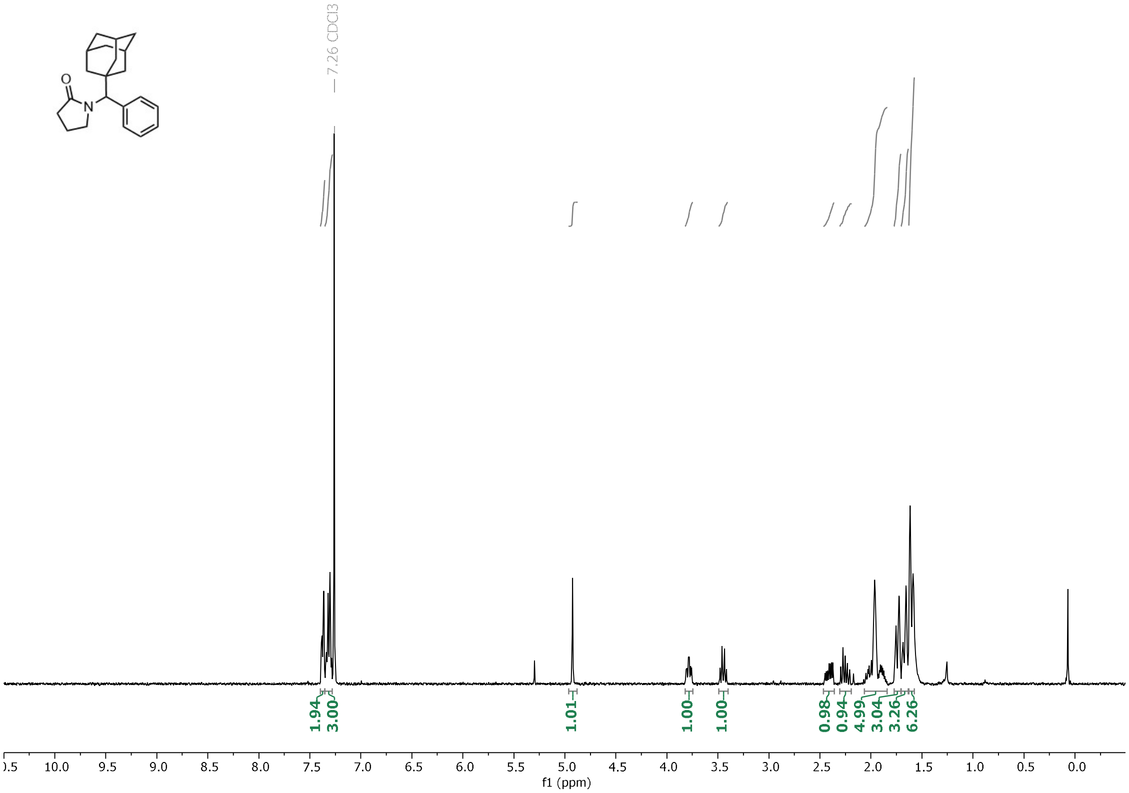

^{13}C NMR (101 MHz, $CDCl_3$) of **22**

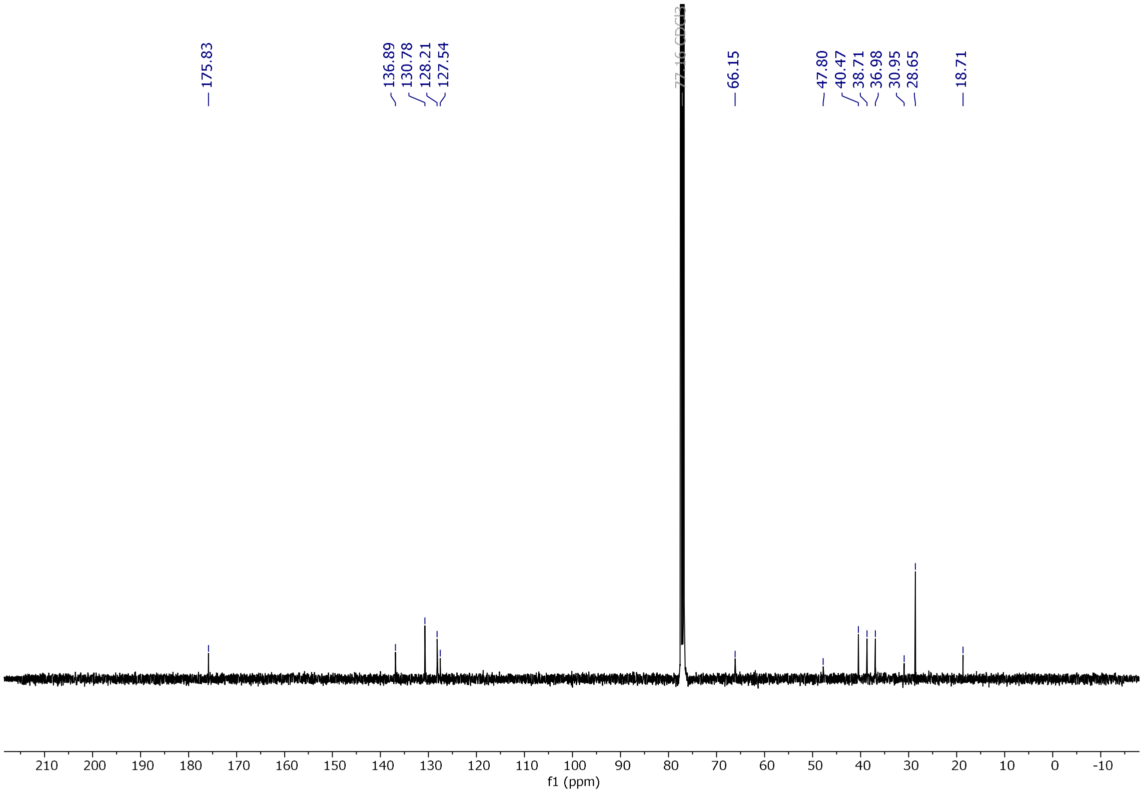

^{1}H NMR (400 MHz, $CDCl_3$) of **23**

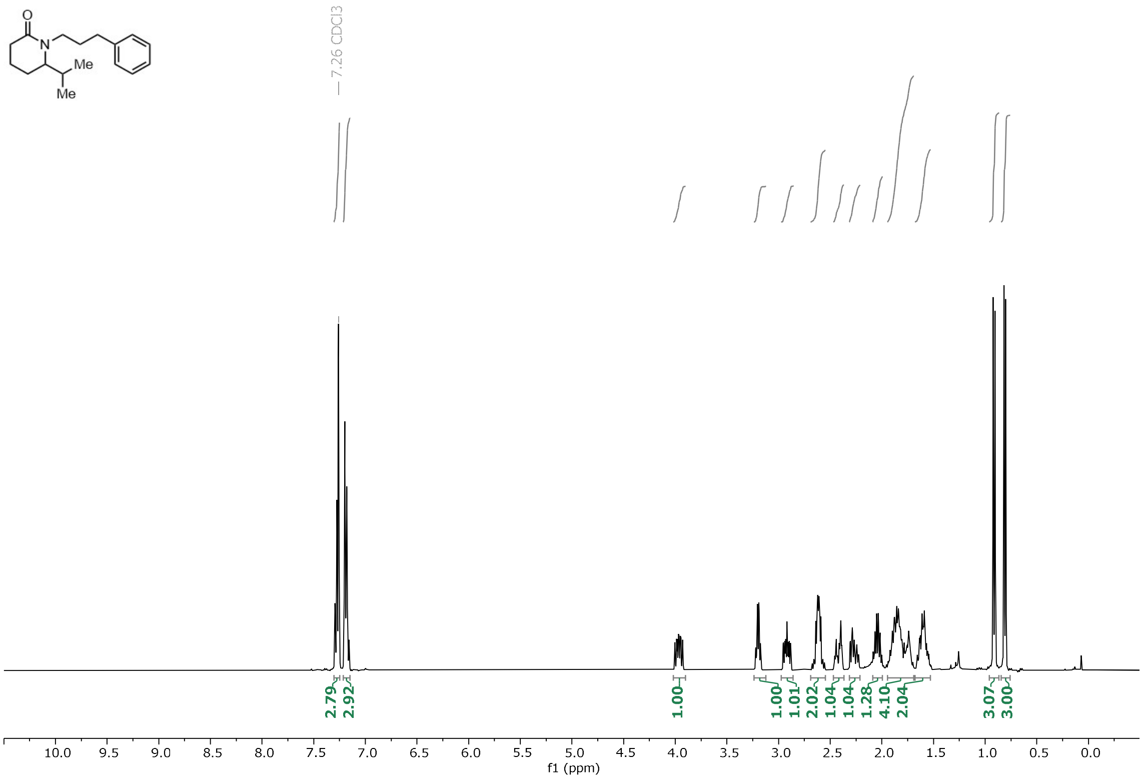

^{13}C NMR (101 MHz, $CDCl_3$) of **23**

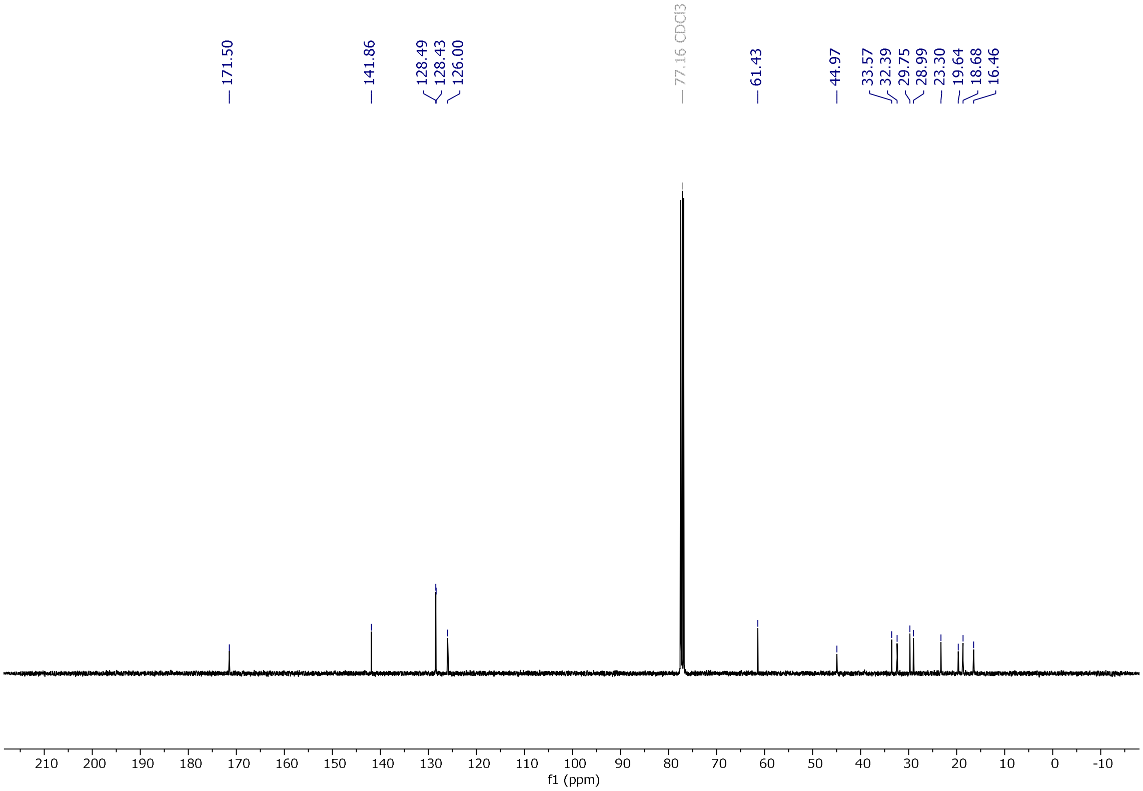

^{1}H NMR (400 MHz, $CDCl_3$) of **24**

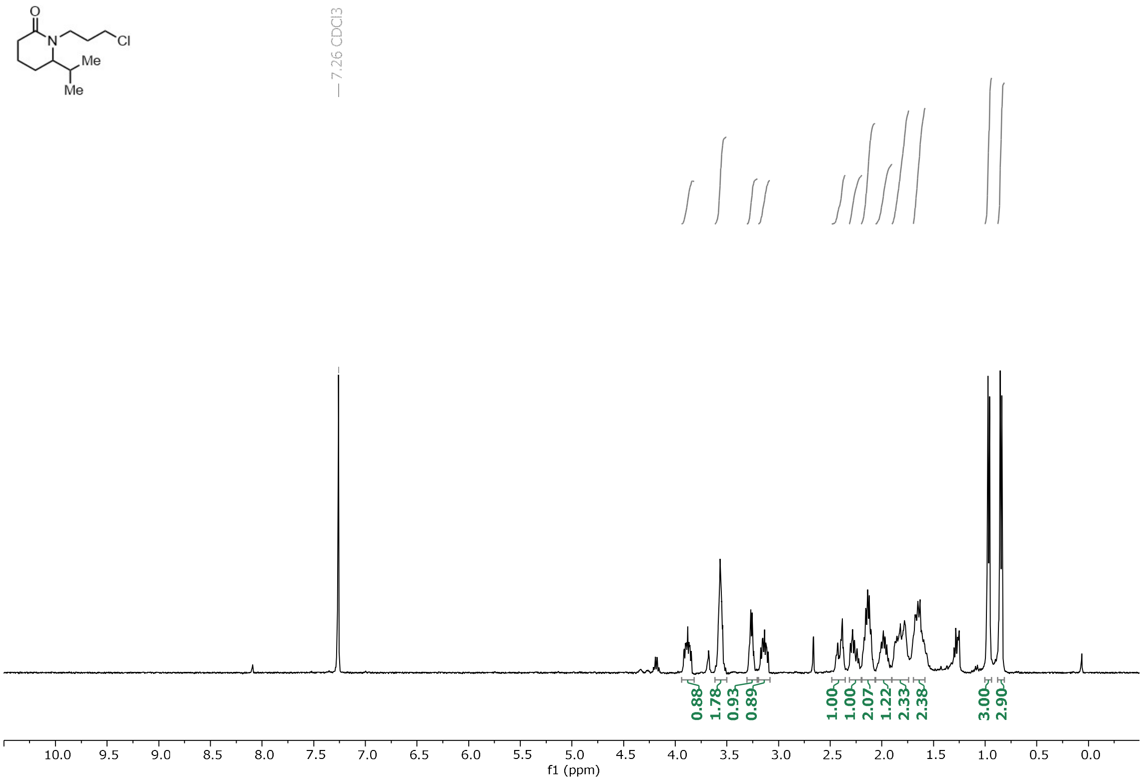

^{13}C NMR (101 MHz, $CDCl_3$) of **24**

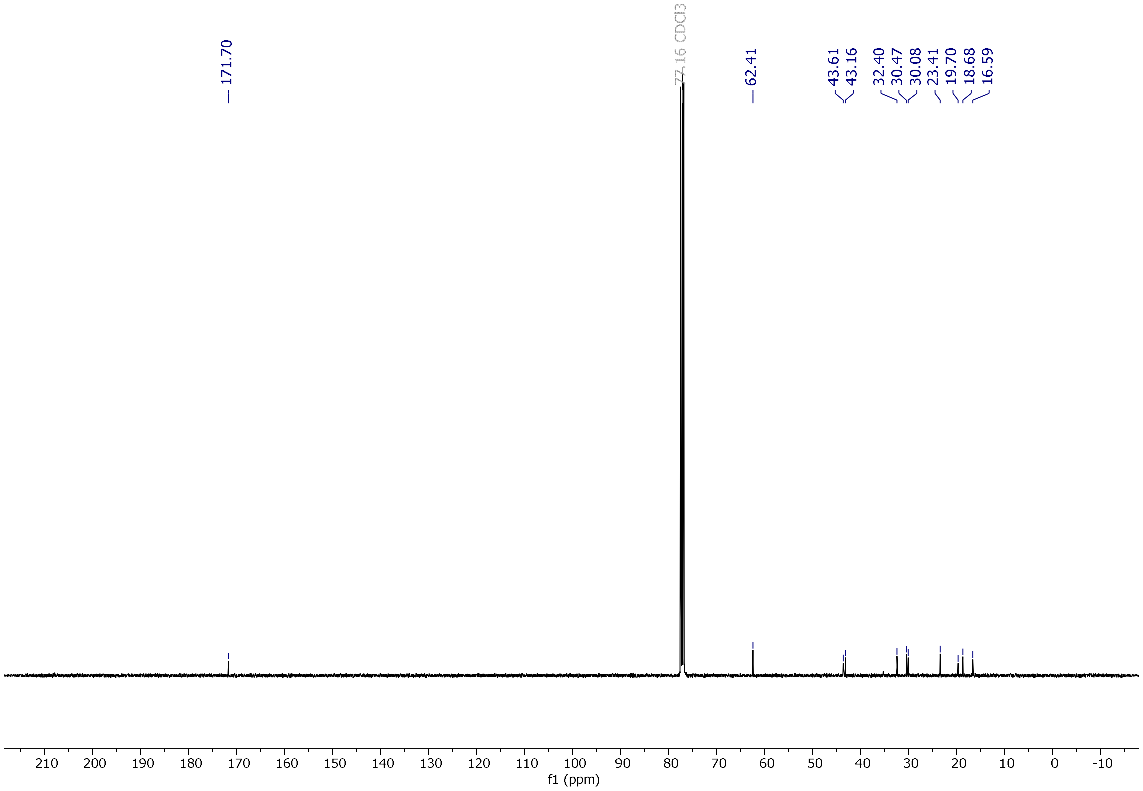

^{1}H NMR (300 MHz, CDCl$_3$) of **25**

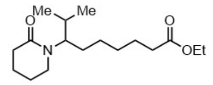

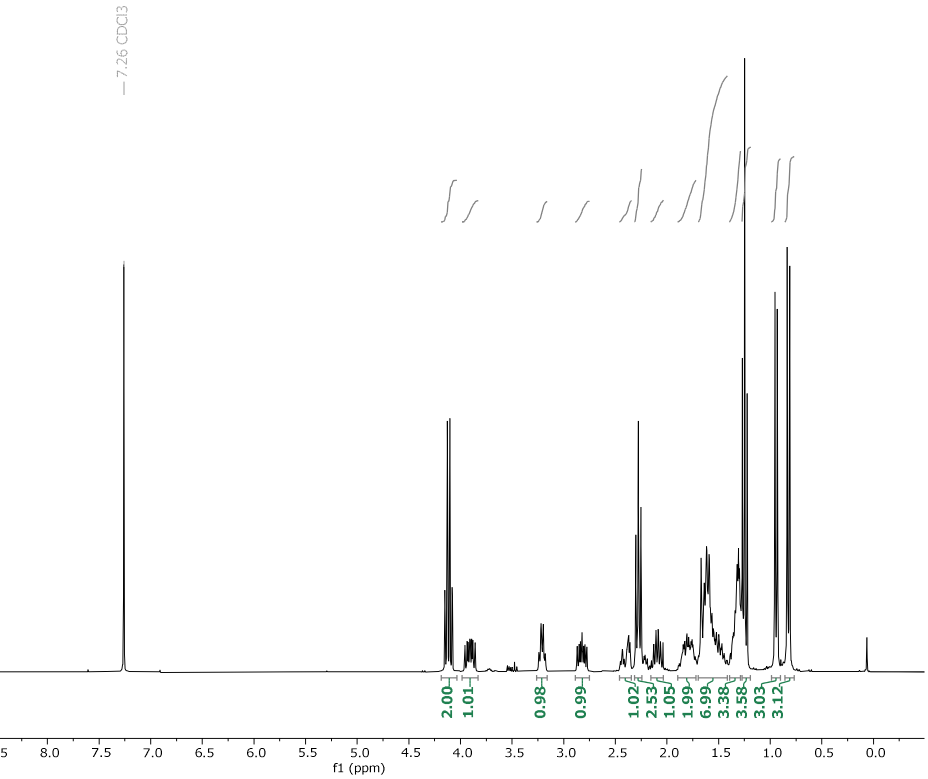

^{13}C NMR (101 MHz, CDCl$_3$) of **25**

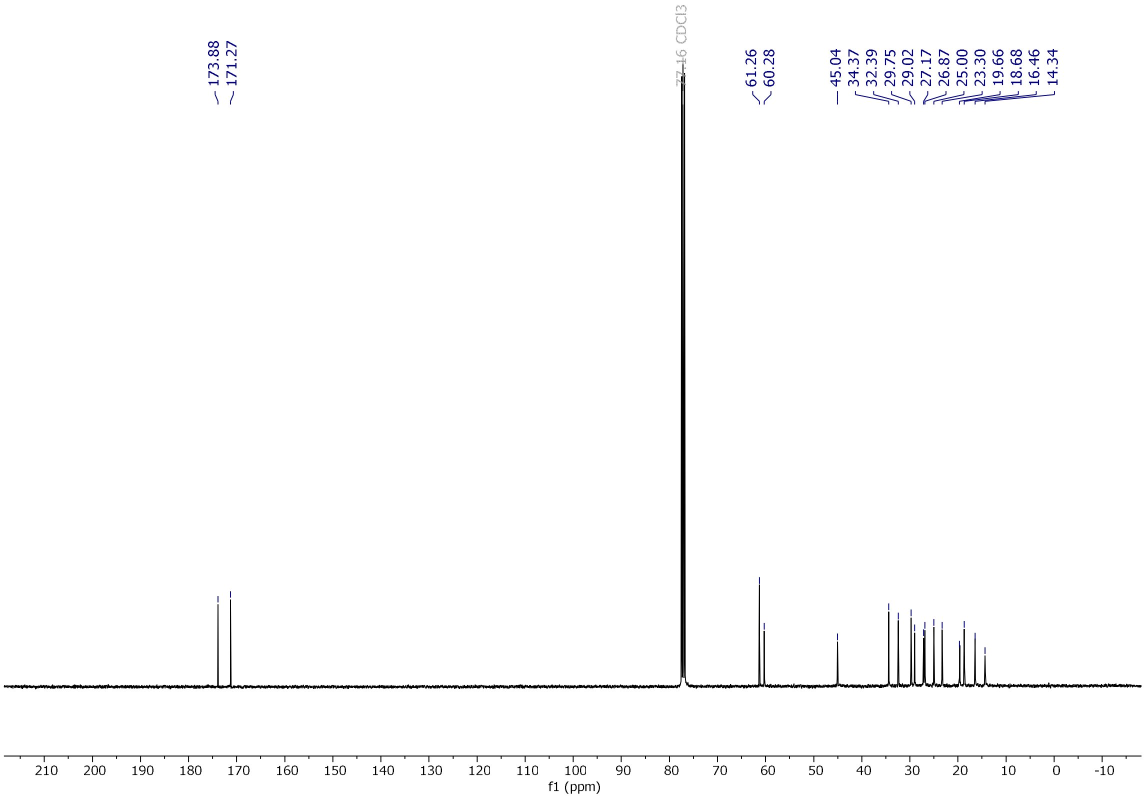

Supplement: Supplementary file 1 [file au5c00884_si_001.pdf]
